# Synthesis of highly functionalized thiazolo[3,2-*a*]pyridine derivatives *via* a five-component cascade reaction based on nitroketene *N*,*S*-acetal†

**DOI:** 10.1039/d0ra03910a

**Published:** 2020-08-21

**Authors:** Zohreh Sahhaf Razavi, Mohammad Bayat, Hajar Hosseini

**Affiliations:** Department of Chemistry, Faculty of Science, Imam Khomeini International University Qazvin Iran bayat_mo@yahoo.com m.bayat@sci.ikiu.ac.ir

## Abstract

A highly efficient and straightforward synthesis of N-fused heterocyclic compounds including 5-amino-7-(aryl)-8-nitro-*N*'-(1-(aryl)ethylidene)-3,7-dihydro-2*H*-thiazolo[3,2-*a*]pyridine-6-carbohydrazide derivatives is successfully achieved *via* a five-component cascade reaction utilizing cyanoacetohydrazide, various acetophenones, aromatic aldehydes, 1,1-bis(methylthio)-2-nitroethylene and cysteamine hydrochloride in ethanol at reflux conditions. The new approach involves domino *N*,*S*-acetal formation, Knoevenagel condensation, Michael reaction, imine–enamine tautomerization and *N*-cyclization sequences. The prominent advantages of this protocol include: facility of operation, available and economical starting materials, no need for toxic solvents, high yields and tolerance of a wide variety of functional groups.

## Abstract 2

Easy construction of novel and highly substituted thiazolo[3,2-*a*]pyridine hydrazone compounds using keten *N*,*S*-acetals.

## Graphical abstract

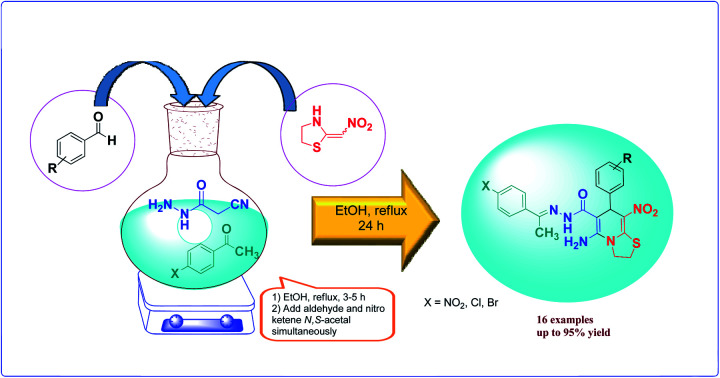

## Introduction

**Fig. 1:**
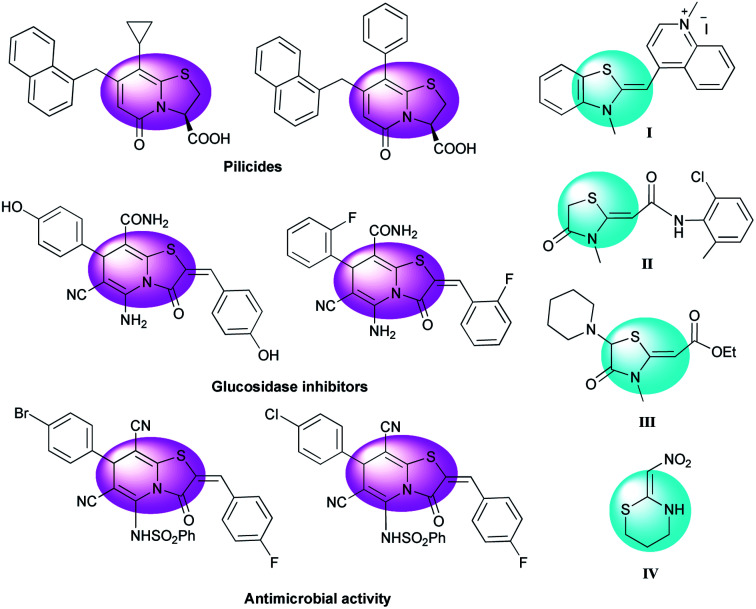
Selected examples of thiazolo[3,2-*a*]pyridines with biological and pharmacological activities and examples of drugs, insecticides and probes having the ketene *N*,*S*-acetal structure.

The thiazolopyridine moiety is found in a wide spectrum of biologically active compounds. Thiazolo[3,2-*a*]pyridines are an important category with notable antibacterial and antifungal activity^1^ and other considerable bioactivities including as a beta-amyloid production inhibitor,^2^ potent CDK2-cyclin A inhibitor,^3^ potential uterus stimulant,^4^ coronary dilator, antihypertensives, and muscle relaxant.^5^ Also they are useful for chemotherapy of various cancers, such as leukemia, lung cancer, and melanoma.^6–8^ Some biologically active compounds with this nucleus are presented in Fig. 1.^9–11^

Obviously, the synthesis of new classes of thiazolo[3,2-*a*]pyridines may give a library of compounds as possible candidates for various biological activities.

Cyclic ketene *N*,*S*-acetal structures are as such used as drugs for the treatment of hypertension diseases and usually employed as probes for nucleic acids to study the interaction between G4 (G-quadruplex) and its ligands (Fig. 1, I–III).^12^ It's interesting that the cyclic nitroketene *N*,*S*-acetal nithiazine IV was the first reported compound of neonicotinoid insecticides^13^ and is widely used as a common insecticide around the world (Fig. 1). Synthetically, the cyclic nitroketene *N*,*S*-acetals have a rigid structure and act as Michael donor 1,3-*N*,*C* di-nucleophiles for the generation of nitrogen-containing heterocyclic compounds. The ethylene motif has a polarized push–pull type of alkene, therefore the one end expands an electrophilic character, whereas the other end develops a nucleophilic character. This feature of nitroketene *N*,*S*-acetals make them highly useful to apply in the Michael addition, annulation and multicomponent reactions.^14^ Today, multicomponent reactions (MCRs) have become a prominent strategy and are selected over stepwise synthesis due to the following reasons: reduced synthetic time, labor and cost, minimal utilization of toxic and harmful chemicals, simple workup of products, high yields, straight forward and simplicity of experimental procedures and economic viability; therefore, MCRs are a powerful approach to promotion of green chemistry by reducing the formation of large quantities of waste.^15–19^

**Scheme 1:**
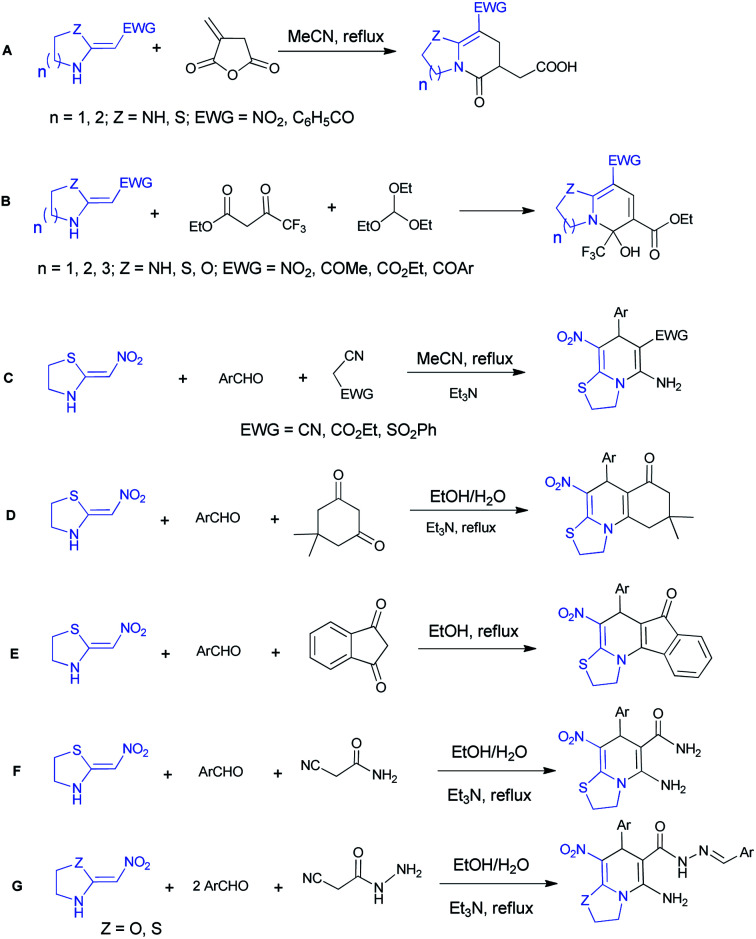
Summary of previous works of thiazolo[3,2-*a*]pyridine synthesis.

The five-membered cyclic nitroketene *N*,*S*-acetal and commercially available six-membered nithiazine have been remarkably explored in the literature and their reactions with different Michael acceptors are most expected. Here we report the some synthesis of thiazolo[3,2-*a*]pyridine compounds performed with cyclic ketene *N*,*S*-acetals (Scheme 1). In 2005, Chakrabarti *et al.* described the reactions between diverse cyclic *N*,*S*- and *N*,*N*-ketene acetals and itaconic anhydride (A).^20^ In 2010, Yan *et al.* reported one-pot synthesis of functionalized bicyclic pyridines under solvent- and catalyst-free conditions with triethoxymethane, ethyl 4,4,4-trifluoro-3-oxobutanoate and various ketene aminals (B).^21^ In 2011, Altug *et al.* developed the synthesis of thiazolo[3,2-*a*]pyridines *via* a one-pot reaction between 2-(nitromethylene)thiazolidine, aromatic aldehydes and ethyl 2-cyanoacetate, malononitrile or 2-(phenylsulfonyl)acetonitrile (C).^22^

In 2018, our research group synthesized fused thiazolo[3,2-*a*]pyridines utilizing the five-membered cyclic nitroketene *N*,*S*-acetal, dimedone and different aromatic aldehydes (D).^23^ Also we reported the synthesis of indenone-fused thiazolo[3,2-*a*]pyridines *via* a one-pot reaction between 2-(nitromethylene)thiazolidine, aromatic aldehydes and 1,3-indandione (E).^24^ In addition, we were able to produce the desired products using cyanoacetamide, aromatic aldehydes and 2-(nitromethylene)thiazolidine (F).^25^ Moreover, in 2018, the reaction of cyanoacetohydrazide with aromatic aldehydes and 2-(nitromethylene)thiazolidine/oxazolidine resulted in functionalized thiazolo/oxazolo pyridine derivatives (G).^26^

Following our efforts to synthesize the new heterocyclic compounds using cyanoacetohydrazide and based on previous works, we designed new reactions utilizing 2-(nitromethylene)thiazolidine as heterocyclic ketene aminal. In this article we report an efficient synthesis of highly functionalized 2*H*-thiazolo[3,2-*a*]pyridine-6-carbohydrazide compounds *via* a one-pot five-component domino reaction. To the best of our knowledge, there is no report on the synthesis of these structures.

## Results and discussion

**Scheme 2:**
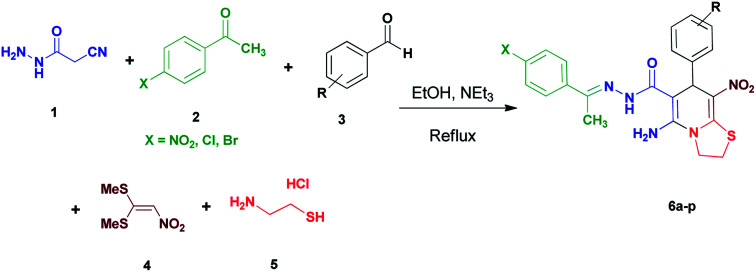
Synthetic scheme for the generation of products 6a–p.

We have developed an efficient synthesis of new functionalized thiazolo[3,2-*a*]pyridine structures 6 by using of cyanoacetohydrazide 1, acetophenone derivatives 2, aromatic aldehydes 3, 1,1-bis(methylthio)-2-nitroethene 4 and cysteamine hydrochloride 5 in the presence of triethylamine in ethanol at reflux conditions (Scheme 2).

### Optimization of the conditions

Initially, cyanoacetohydrazide 1, 4-chloroacetophenone 2, 4-chlorobenzaldehyde 3, 1,1-bis(methylthio)-2-nitroethene 4 and cysteamine hydrochloride 5 were used as model substrates to achieve the best yield.

In general, due to the variable reactivity of cyanoacetohydrazide (based on its specific structure) and on the other hand due to the five-component nature of the defined reactions, great efforts were made to obtain the desired products with high purity. At first ethanol was examined and the experimental results showed when ethanol was used as solvent with triethylamine at reflux conditions, the yield of desired product 6a was 93% (Table 1, entry 1). It should be noted that the catalyst used (NEt_3_) is not working on the rate-limiting step. To prepare 2-(nitromethylene)thiazolidine solution (from 1,1-bis(methylthio)-2-nitroethene and cysteamine hydrochloride, which is mentioned in the Experimental section), it is necessary to use triethylamine to separate cysteamine from its salt.^23,27^ No reaction will occur without the use of triethylamine (entry 4). The use of other catalysts is related to the whole reaction.

**Table 1:** Optimization conditions for the formation of 6a^a^

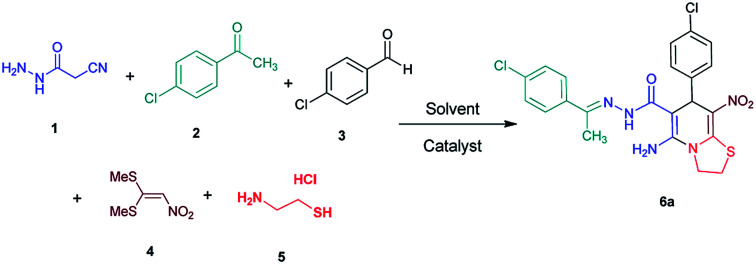					
Entry	Solvent	Catalyst (mol%)	Time (h)	Temp (°C)	Yield (%)
1	EtOH	NEt_3_	24	78	93
2	EtOH	Piperidine	24	78	80
3	EtOH	AcOH	24	78	No reaction
4	EtOH	—	24	78	No reaction
5	H_2_O	NEt_3_	24	100	No reaction
6	H_2_O/EtOH (1 : 1, v/v)	NEt_3_	24	78	40
7	CH_3_CN	NEt_3_	24	82	No reaction
8	CHCl_3_	NEt_3_	24	61	No reaction
9	MeOH	NEt_3_	24	65	No reaction
10	DMF	NEt_3_	24	153	No reaction

a Reagents and conditions: cyanoacetohydrazide (1 mmol), 4-chloroacetophenone (1 mmol), 4-chlorobenzaldehyde (1 mmol), 1,1-bis(methylthio)-2-nitroethene (1 mmol), cysteamine hydrochloride (1 mmol), solvent (20 mL), catalyst (1 mmol).

In order to increase the reaction rate, two types of catalysts were used. With piperidine, the reaction efficiency decreased slightly (entry 2) and with acetic acid, the product did not form (entry 3). According to the investigations, it was determined that in basic and acidic medium, other products are formed (two-, three- and four-component products). The percentage of each was different for various derivatives. In general, it was found that the slightest change in the reaction conditions (even in ethanol amount) leads to a decrease in the efficiency of the desired product or often its non-formation. In addition, we observed the formation of a four-component by-product in two cases, which are described in the general procedure section. However, we also studied the effect of other solvents. The use of water or acetonitrile did not result in the desired product (entry 5 and 7), and when the mixture of water and ethanol was used (overall 1 : 1, v/v), the efficiency decreased (entry 6). With chloroform, methanol and DMF, in reflux conditions the desired products were not formed (entry 8, 9 and 10).

With information obtained from optimization conditions table, we could synthesize target compounds (*E*)-5-amino-7-(aryl)-8-nitro-*N*'-(1-(aryl)ethylidene)-3,7-dihydro-2*H*-thiazolo[3,2-*a*]pyridine-6-carbohydrazide 6a–p in good to high yields (70–95%) using cyanoacetohydrazide 1, acetophenone derivatives 2, various aromatic aldehydes 3, 1,1-bis(methylthio)-2-nitroethene 4 and cysteamine hydrochloride 5 as starting materials (Scheme 2).

The reactions were completed after 24 h to afford the corresponding heterocyclic structures. The results are summarized in Table 2.

**Table 2:** Compounds 6a–p^a^

Entry	Aromatic aldehyde	Acetophenone derivative	Product	Yield (%)	Mp (°C)
1	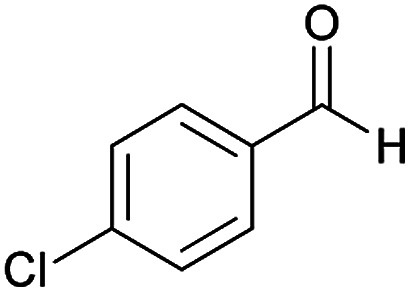	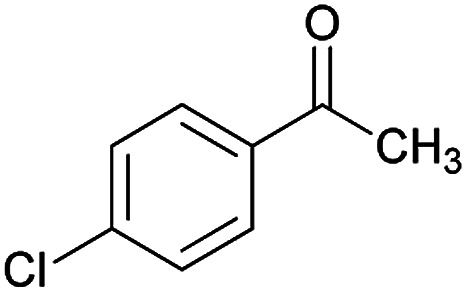	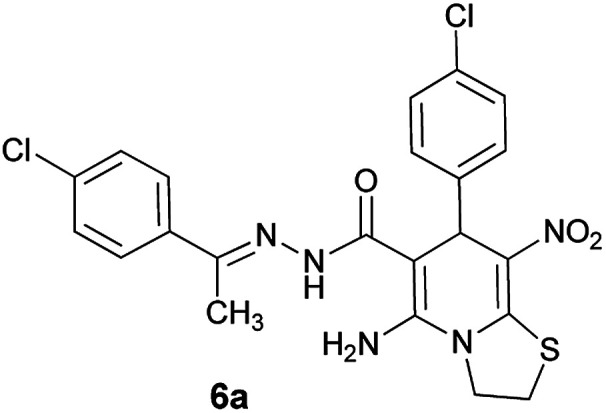	93	252–254
2	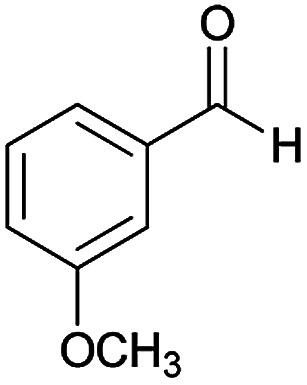	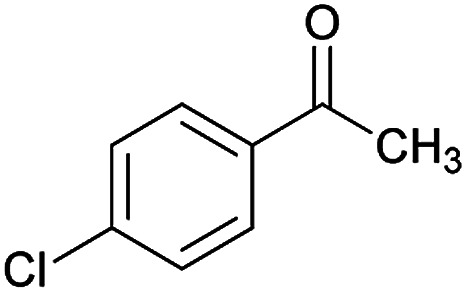	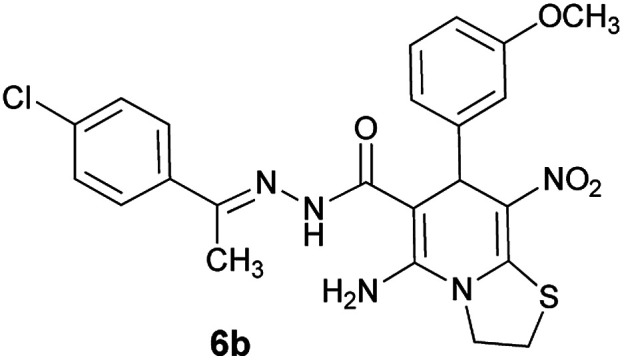	76	230–233
3	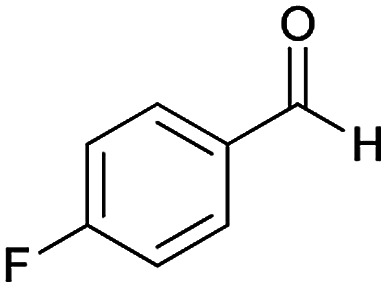	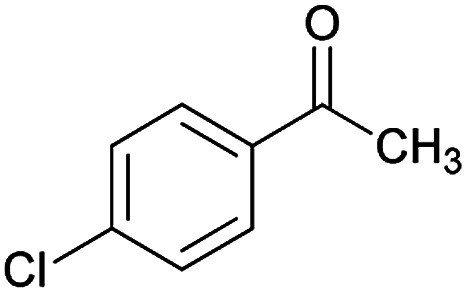	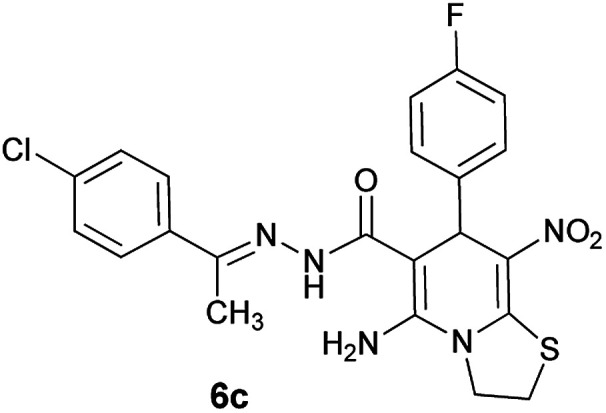	86	239–241
4	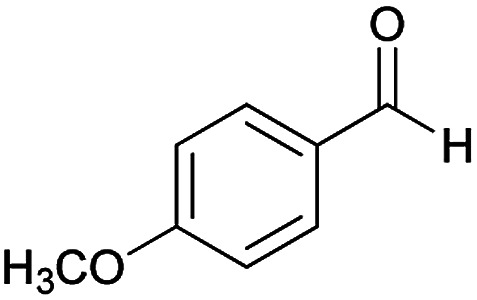	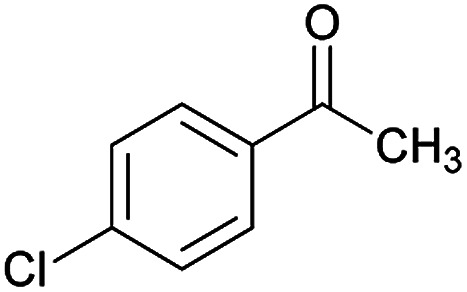	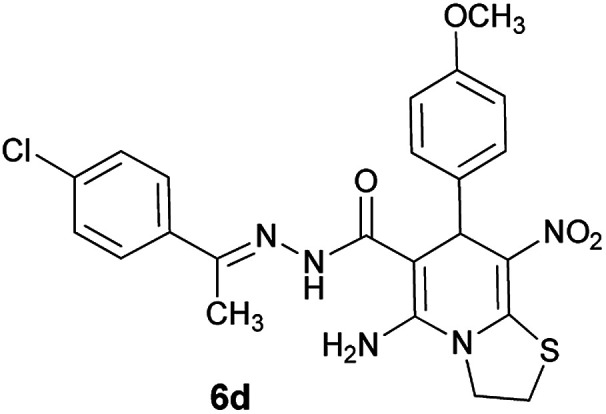	80	222–224
5	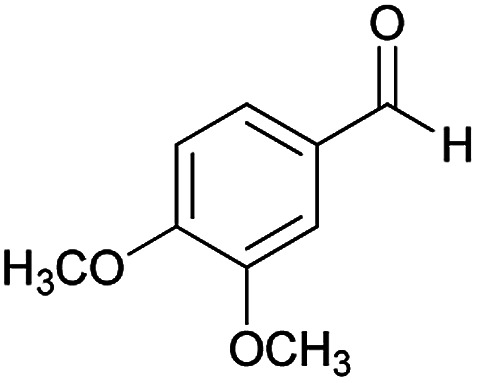	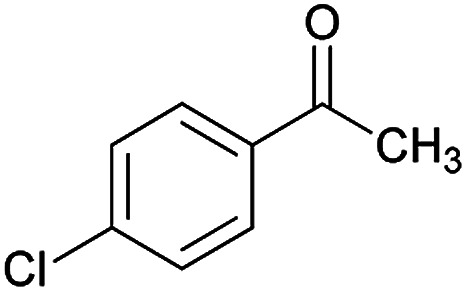	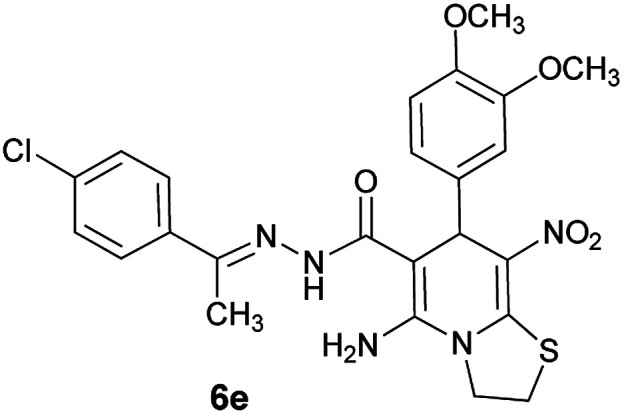	75	217–219
6	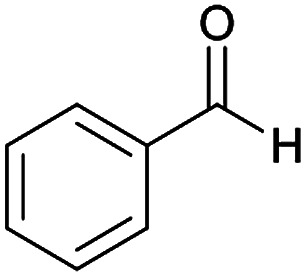	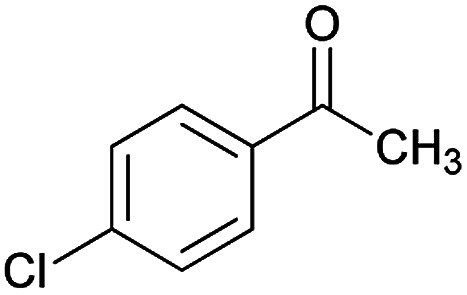	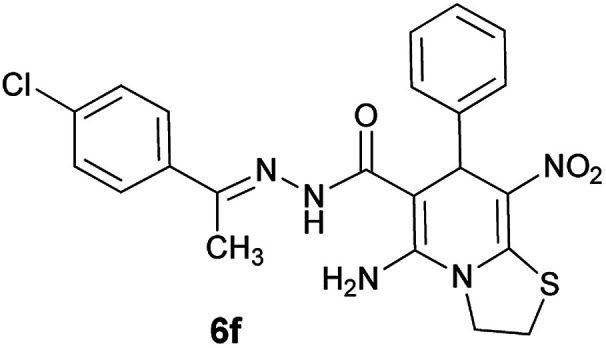	84	248–250
7	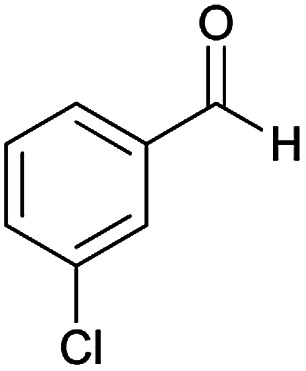	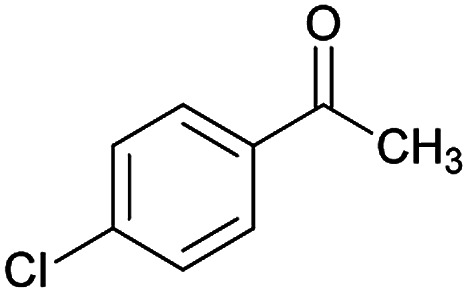	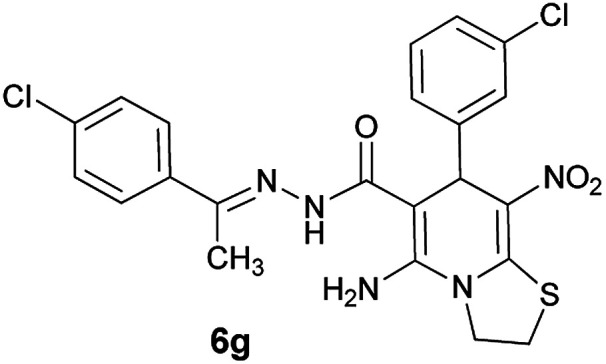	82	237–240
8	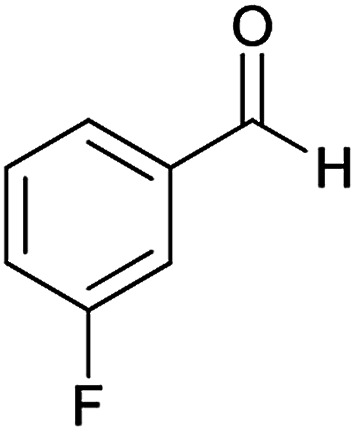	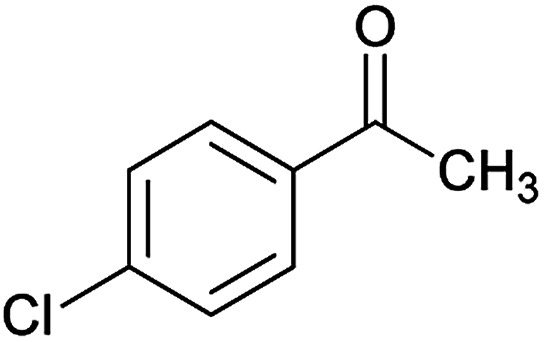	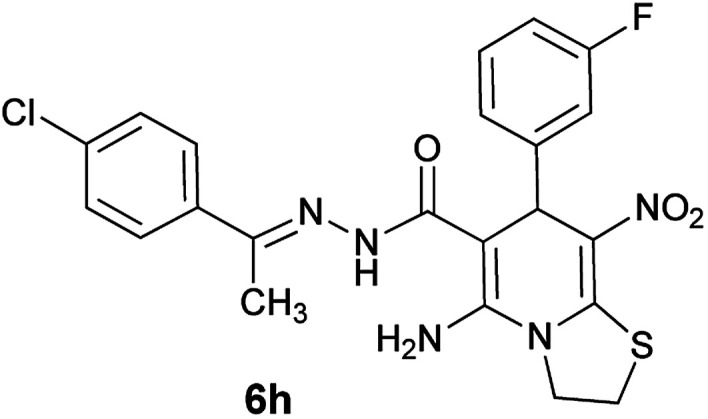	78	225–227
9	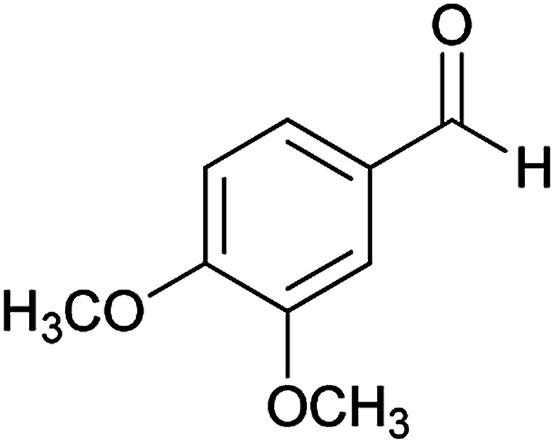	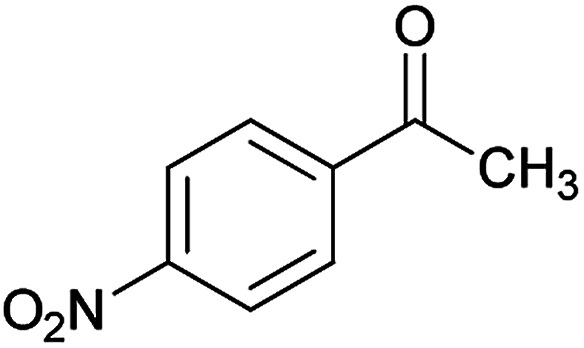	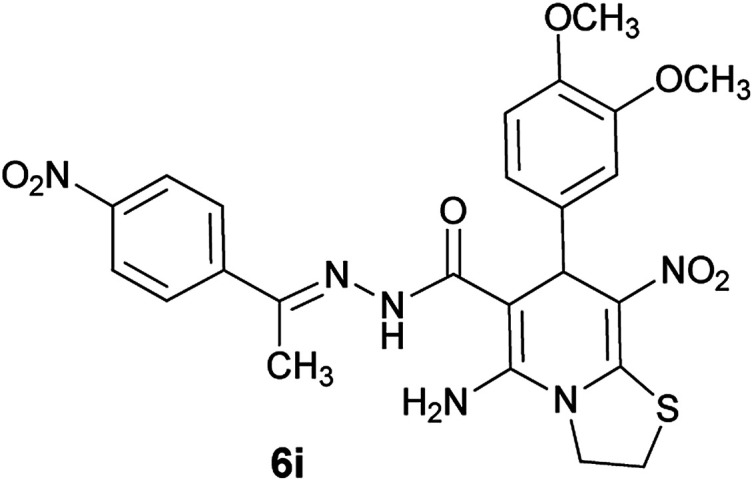	80	203–205
10	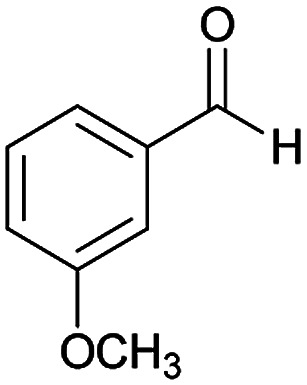	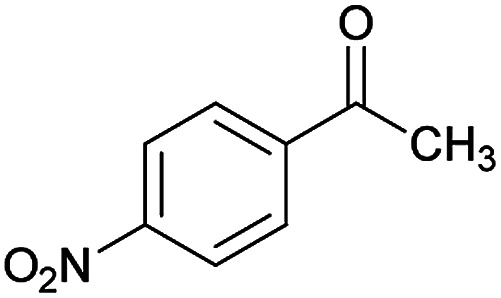	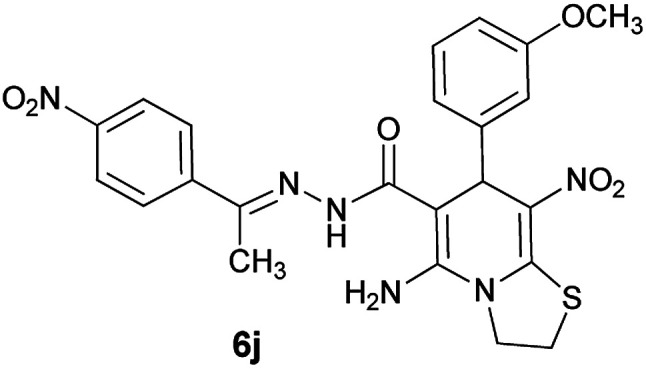	78	234–236
11	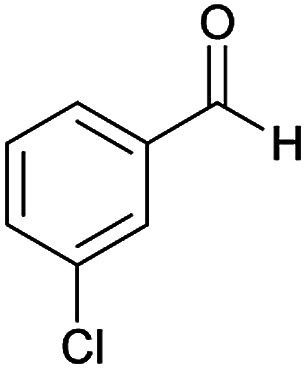	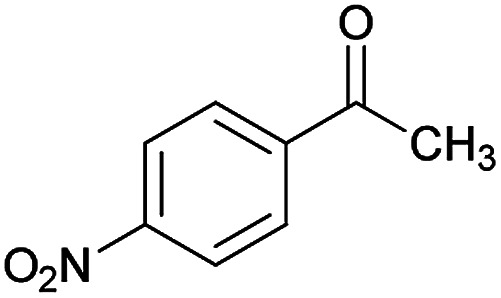	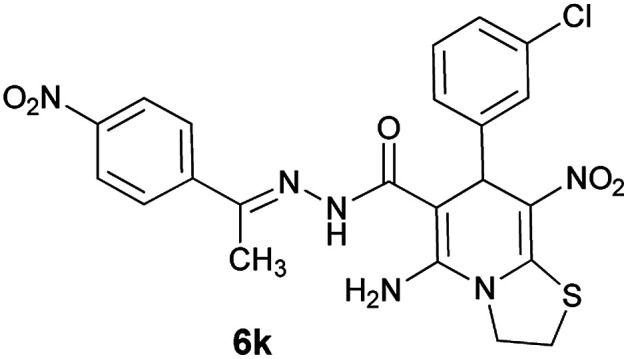	90	218–220
12	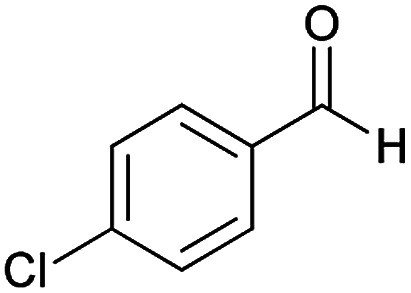	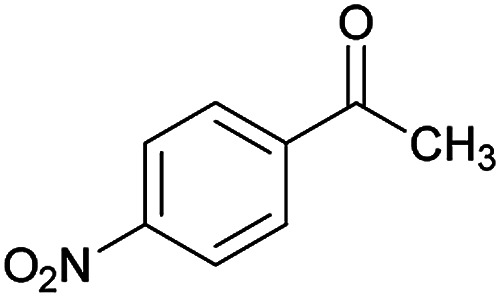	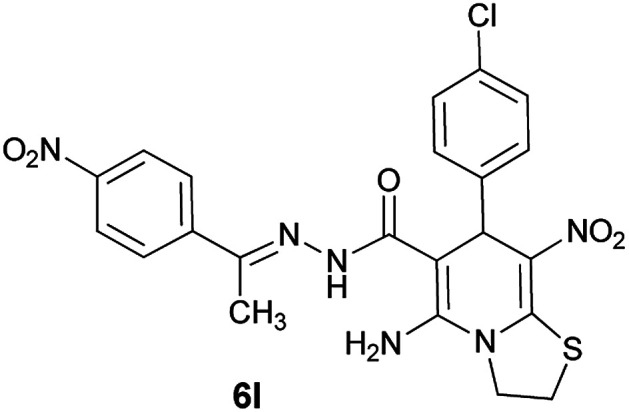	95	244–246
13	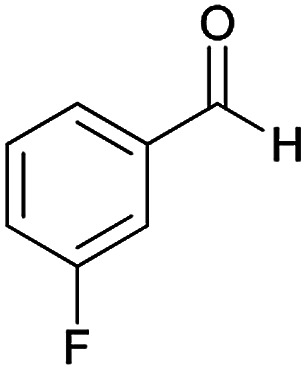	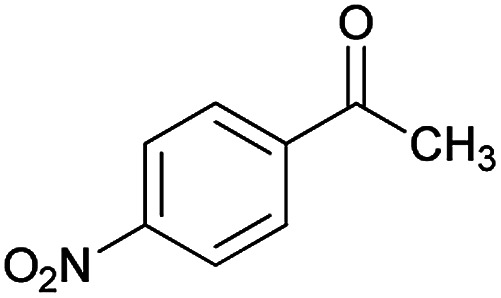	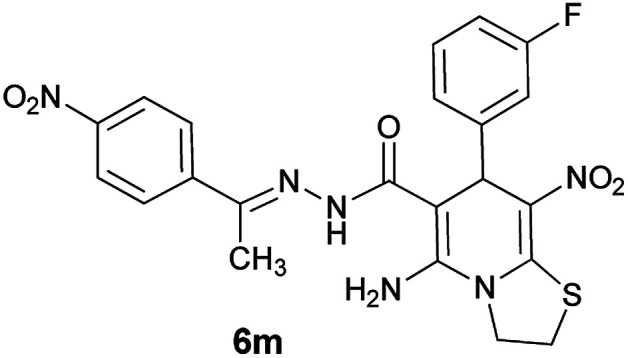	85	242–245
14	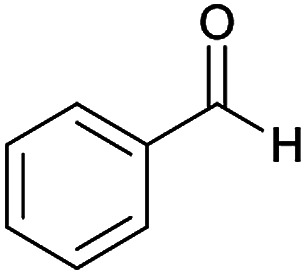	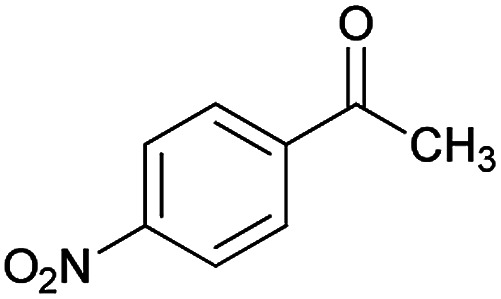	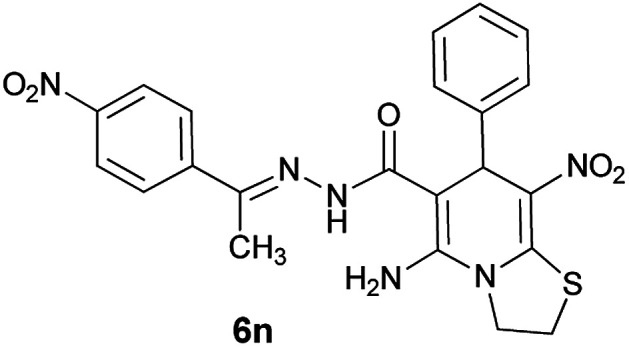	87	244–246
15	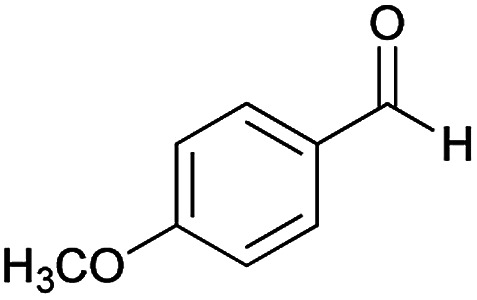	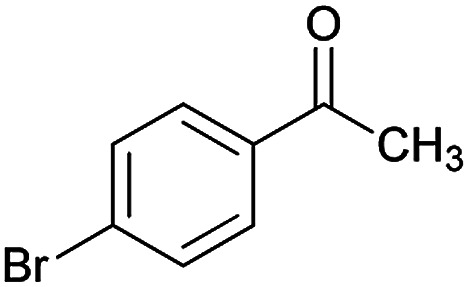	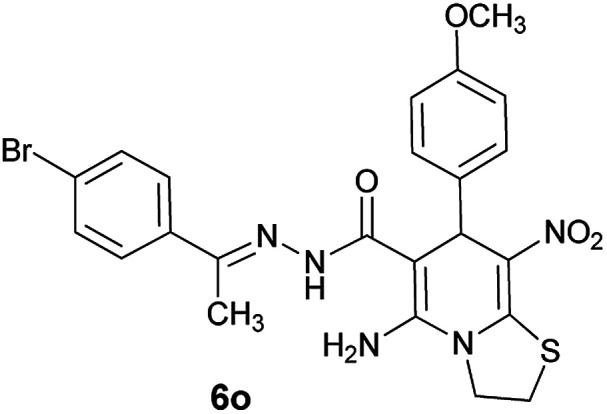	75	253–255
16	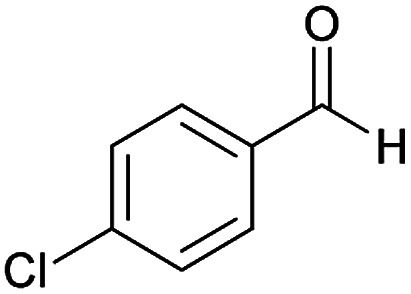	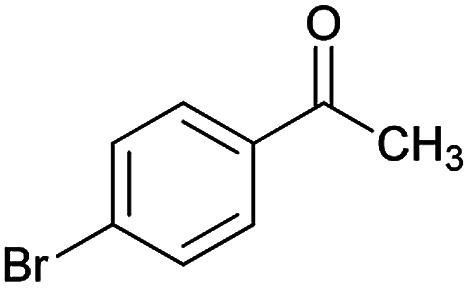	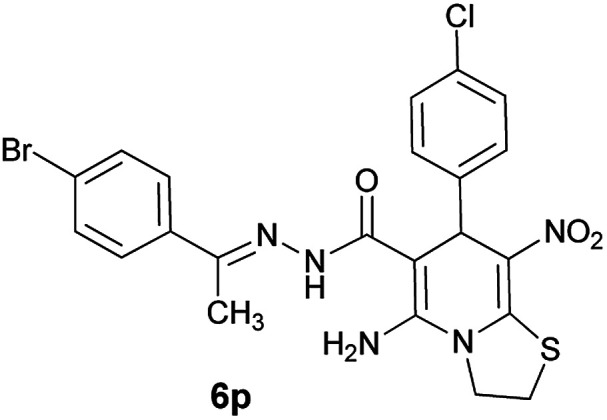	70	262–264

a The reactions were performed using cyanoacetohydrazide (1 mmol), acetophenone derivatives (1 mmol), aromatic aldehydes (1 mmol), 1,1-bis(methylthio)-2-nitroethene, (1 mmol), cysteamine hydrochloride (1 mmol), triethylamine (1 mmol), EtOH (20 mL).

### Scope and limitations

This reaction was performed with *ortho* derivatives of benzaldehyde (2-chloro, 2-hydroxy and 2-nitro) under the same conditions, which did not result in the product probably due to steric effects. Also the use of acetophenone and 4-methoxyacetophenone did not lead to the favorable products. The reaction was also used with aliphatic ketones instead of acetophenone derivatives and aliphatic aldehydes instead of aromatic aldehydes which resulted in no product formation.

It was found that the major by-product of this reaction is a four-component structure that was previously synthesized using two equivalents of aldehyde^26^ which will prevent its formation by performing the correct reaction steps (see Experimental section).

### Structure determination

The structures of all new compounds 6a–p were supported by means of IR, ^1^H NMR, ^13^C NMR spectroscopic and mass spectrometric data (see the ESI†).

**Fig. 2:**
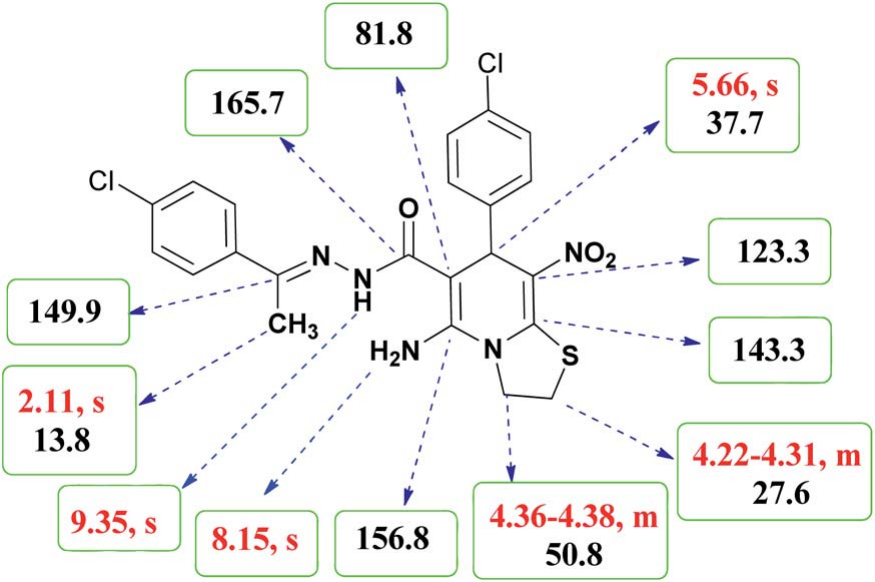
^1^H and ^13^C NMR chemical shifts of 6a.

As a representative case the key signals of ^1^H and ^13^C NMR chemical shifts of (*E*)-5-amino-7-(4-chlorophenyl)-*N*'-(1-(4-chlorophenyl)ethylidene)-8-nitro-3,7-dihydro-2*H*-thiazolo[3,2-*a*]pyridine-6-carbohydrazide 6a are presented in Fig. 2.

The ^1^H NMR spectrum of 6a showed NH group at *δ* 9.35 ppm. The NH_2_ group appeared at *δ* 8.15 ppm. The proton of CH at pyridine ring was seen at *δ* 5.66 ppm. Four protons of two methylene groups appeared at *δ* 4.22 to 4.38 ppm as two multiplets. The signal at *δ* 2.11 ppm was related to methyl group.

The ^1^H-decoupled ^13^C NMR spectrum of 6a indicated 18 distinct resonances in accordance to desired structure. The characteristic signals of four aliphatic carbons (CH_3_, CH and two CH_2_ groups) were seen at *δ* 13.8, 37.7, 27.6 and 50.8 ppm respectively. Characteristic signal at *δ* 81.8 ppm was related to C=*C*–CO. The carbonyl group appeared at *δ* 165.7 ppm (Fig. 2).

The IR spectrum of 6a showed absorption bands at 3141 and 3284 cm^−1^ due to NH and NH_2_ groups, strong absorption of carbonyl group at 1626 and C–N band at 1237 cm^−1^. Two absorption bands due to nitro group appeared at 1514 and 1302 cm^−1^.

### Mechanism

**Scheme 3:**
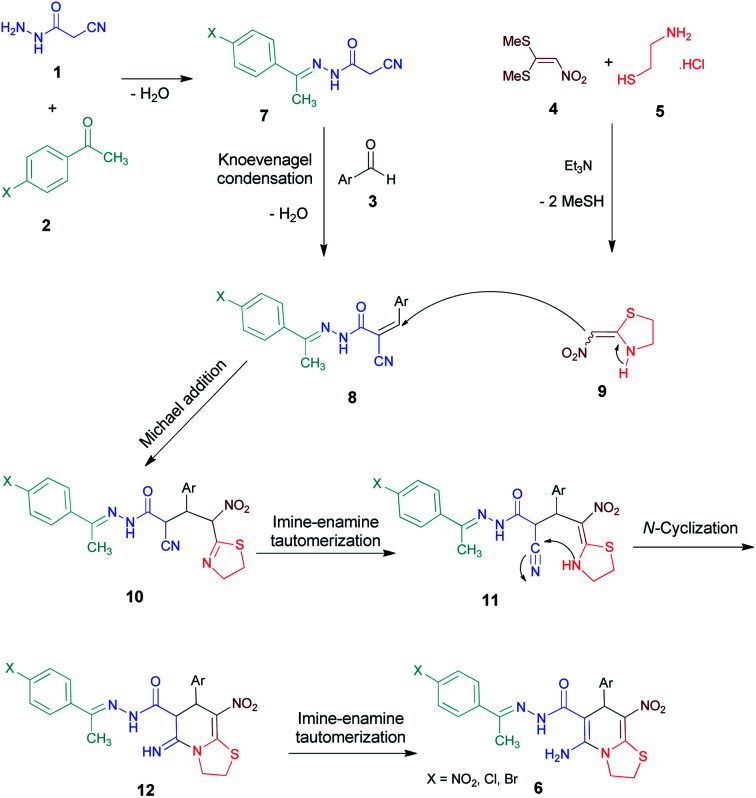
Proposed mechanism for the formation of products 6.

A general plausible mechanism for the formation of thiazolo[3,2-*a*]pyridine carbohydrazides is shown in Scheme 3. The condensation of cyanoacetohydrazide 1 with acetophenone 2 leads to the hydrazide-hydrazone structures 7. On the basis of well-established chemistry of 1,1-bis(methylthio)-2-nitroethene, on the other hand, addition of cysteamine hydrochloride 5 to 1,1-bis(methylthio)-2-nitroethene 4 leads to the formation of ketene *N*,*S*-acetal 9.^23,27^ The formation of β-nitrothiazolidine 9 occurs in the presence of an equivalent amount of triethylamine base for releasing cysteamine salt. Further, with adding aldehyde 3, the Knoevenagel condensation affords intermediate 8. Then, Michael addition of nitroenamine 9 to adduct 8 leads to the intermediate 10, which undergoes successive imine–enamine tautomerization followed by an intramolecular cyclization *via* nucleophilic addition of –NH to nitrile group. Finally, imine–enamine tautomerization leads to thiazolo[3,2-*a*]pyridine products 6 (Scheme 3).

## Conclusion

A green and efficient approach to easy synthesis of novel and highly substituted fused 1,4-dihydropyridines, 5-amino-7-(aryl)-8-nitro-*N*'-(1-(aryl)ethylidene)-3,7-dihydro-2*H*-thiazolo[3,2-*a*]pyridine-6-carbohydrazides, has been developed based on a one-pot five-component condensation *via* annulation of cyclic nitroketene *N*,*S*-acetal, β-nitrothiazolidine, and a three-component product of cyanoacetohydrazide, acetophenone derivatives and different aromatic aldehydes. The reactions are completed within 24 h in EtOH at reflux conditions. The present synthesis shows significant properties such as high regioselectivity, cascade one-pot methodology, high yields, simple purification of products, and high atom economy.

## Experimental

### Materials

All commercially available reagents and other solvents were purchased from Aldrich and Merck chemical Co. and used without further purification. The NMR spectra were recorded with a Bruker DRX-300 AVANCE instrument (300 MHz for ^1^H and 75.4 MHz for ^13^C) with DMSO-*d*_6_ as solvent. Chemical shifts are given in ppm (*δ*) relative to internal TMS, and coupling constant (*J*) are reported in Hertz (Hz). Melting points were measured with an electrothermal 9100 apparatus. Mass spectra were recorded with an Agilent 5975C VL MSD with Triple-Axis detector operating at an ionization potential of 70 eV. IR spectra were measured with Bruker Tensor 27 spectrometer. Elemental analyses for C, H and N were performed using a PerkinElmer 2004 series [II] CHN elemental analyzer.

### General procedure of the synthesis of 5-amino-7-(aryl)-8-nitro-*N*'-(1-(aryl)ethylidene)-3,7-dihydro-2*H*-thiazolo[3,2-*a*]pyridine-6-carbohydrazide derivatives

A mixture of cysteamine hydrochloride (0.113 g, 1 mmol), 1,1-bis(methylthio)-2-nitroethylene (0.165 g, 1 mmol), Et_3_N (140 μL, 1 mmol) and 10 mL EtOH in a 50 mL flask was refluxed for 5 hours. In another 50 mL flask the stoichiometric mixture of cyanoacetohydrazide (1 mmol, 0.099 g) and acetophenone derivative (1 mmol) in EtOH (10 mL) was refluxed for 3–5 hours depending on the type of acetophenone. After these times, TLC shows the consumption of the starting components. Then, aromatic aldehyde (1 mmol) and the first solution (HKA), were added to the second mixture simultaneously. The progress of the reaction was monitored by TLC using ethyl acetate/*n*-hexane (1 : 1). After completion of the reaction (24 hours), without the need for chromatography or recrystallization, the precipitated product was collected by filtration and washed with warm ethanol to give the pure products 6a–p in excellent yield.

To achieve the pure products, it was necessary to complete the reaction of cyanoacetohydrazide and acetophenone derivatives in ethanol at reflux conditions in sufficient time (3 hours for 4-nitroacetophenone and 5 hours for 4-chloro and 4-bromoacetophenone), then with no need for product separation, nitroenamine solution and aromatic aldehyde were added to two-component hydrazone mixture at the same time. We found two distinct cases (6g and 6h) that led to a mixture of two products: the desired product and the product without participation of acetophenone derivative.^26^

#### (*E*)-5-Amino-7-(4-chlorophenyl)-*N*'-(1-(4-chlorophenyl)ethylidene)-8-nitro-3,7-dihydro-2*H*-thiazolo[3,2-*a*]pyridine-6-carbohydrazide (6a)

Yellow solid; yield: 0.468 g (93%); mp: 252–254 °C; IR (KBr) (*ν*_max_/cm^−1^): 3284, 3141, 1626, 1514, 1452, 1302, 1237, 1131, 850, 756; ^1^H NMR (300 MHz, DMSO): *δ* 2.11 (3H, s, CH_3_), 4.22–4.31 (2H, m, CH_2_), 4.36–4.38 (2H, m, CH_2_), 5.66 (1H, s, CH), 7.32–7.39 (4H, m, ArH), 7.43 (2H, d, *J* = 8.4 Hz, ArH), 7.75 (2H, d, *J* = 8.4 Hz, ArH), 8.15 (2H, s, NH_2_), 9.35 (1H, s, NH); ^13^C{^1^H} NMR (125.6 MHz, DMSO): *δ* 13.8 (CH_3_), 27.6 (CH_2_S), 37.7 (CH), 50.8 (CH_2_N), 81.8 (C=**C**–CO), 123.3 (C–NO_2_), 124.3, 127.7, 128.1, 128.2, 129.7, 131.3, 133.5, 137.1 (Ar), 143.3 (C=**C**–S), 149.9 (C=N), 156.8 (C–NH_2_), 165.7 (C=O); anal. calcd for C_22_H_19_Cl_2_N_5_O_3_S: C, 52.39; H, 3.80; N, 13.88. Found: C, 52.7; H, 3.5; N, 13.7.

#### (*E*)-5-Amino-*N*'-(1-(4-chlorophenyl)ethylidene)-7-(3-methoxyphenyl)-8-nitro-3,7-dihydro-2*H*-thiazolo[3,2-*a*]pyridine-6-carbohydrazide (6b)

Yellow solid; yield: 0.379 g (76%); mp: 230–233 °C; IR (KBr) (*ν*_max_/cm^−1^): 3486, 3400, 3327, 2909, 1658, 1519, 1458, 1375, 1254, 785; ^1^H NMR (300 MHz, DMSO): *δ* 2.10 (3H, s, CH_3_), 3.69 (3H, s, OCH_3_), 4.21–4.30 (2H, m, CH_2_), 4.38–4.41 (2H, m, CH_2_), 5.57 (1H, s, CH), 6.77 (1H, d, *J* = 7.8 Hz, ArH), 6.91–6.94 (2H, m, ArH), 7.20 (1H, t, *J* = 7.5 Hz, ArH), 7.43 (2H, d, *J* = 8.4 Hz, ArH), 7.75 (2H, d, *J* = 8.4 Hz, ArH), 8.14 (2H, s, NH_2_), 9.22 (1H, s, NH); ^13^C{^1^H} NMR (125.6 MHz, DMSO): *δ* 13.6 (CH_3_), 27.6 (CH_2_S), 38.2 (CH), 50.7 (CH_2_N), 54.9 (OCH_3_), 82.0 (C=**C**–CO), 111.4, 114.4, 120.0 (Ar), 123.5 (C–NO_2_), 127.7, 128.2, 129.4, 133.5, 137.1 (Ar), 145.8 (C=**C**–S), 149.0 (Ar), 150.0 (C=N), 156.5 (C–NH_2_), 159.0 (C_Ar_–OMe), 165.3 (C=O); anal. calcd for C_23_H_22_ClN_5_O_4_S: C, 55.25; H, 4.44; N, 14.01. Found: C, 55.6; H, 4.7; N, 14.3.

#### (*E*)-5-Amino-*N*'-(1-(4-chlorophenyl)ethylidene)-7-(4-fluorophenyl)-8-nitro-3,7-dihydro-2*H*-thiazolo[3,2-*a*]pyridine-6-carbohydrazide (6c)

Dark yellow solid; yield: 0.418 g (86%); mp: 239–241 °C; ^1^H NMR (300 MHz, DMSO): *δ* 2.08 (3H, s, CH_3_), 4.23–4.27 (2H, m, CH_2_), 4.35–4.39 (2H, m, CH_2_), 5.63 (1H, s, CH), 7.06–7.12 (2H, m, ArH), 7.39–7.43 (4H, m, ArH), 7.74 (2H, d, *J* = 8.4 Hz, ArH), 8.14 (2H, s, NH_2_), 9.30 (1H, s, NH); ^13^C{^1^H} NMR (125.6 MHz, DMSO): *δ* 13.7 (CH_3_), 27.6 (CH_2_S), 37.5 (CH), 50.8 (CH_2_N), 82.1 (C=**C**–CO), 114.8, 114.9 (Ar), 123.4 (C–NO_2_), 127.7, 128.2, 129.7, 129.8, 133.5, 137.1, 149.7 (Ar), 140.6 (C=**C**–S), 149.9 (C=N), 156.6 (C–NH_2_), 159.9, 161.7 (Ar), 165.7 (C=O); anal. calcd for C_22_H_19_ClFN_5_O_3_S: C, 54.15; H, 3.92; N, 14.35. Found: C, 54.3; H, 3.6; N, 14.1.

#### (*E*)-5-Amino-*N*'-(1-(4-chlorophenyl)ethylidene)-7-(4-methoxyphenyl)-8-nitro-3,7-dihydro-2*H*-thiazolo[3,2-*a*]pyridine-6-carbohydrazide (6d)

Orange solid; yield: 0.399 g (80%); mp: 222–224 °C; IR (KBr) (*ν*_max_/cm^−1^): 3272, 3128, 1626, 1484, 1442, 1303, 1233, 1129, 1013, 825; ^1^H NMR (300 MHz, DMSO): *δ* 2.10 (3H, s, CH_3_), 3.68 (3H, s, OCH_3_), 4.22–4.28 (2H, m, CH_2_), 4.35–4.40 (2H, m, CH_2_), 5.51 (1H, s, CH), 6.83 (2H, d, *J* = 8.7 Hz, ArH), 7.28 (2H, d, *J* = 8.7 Hz, ArH), 7.43 (2H, d, *J* = 8.4 Hz, ArH), 7.75 (2H, d, *J* = 8.4 Hz, ArH), 8.12 (2H, s, NH_2_), 9.17 (1H, s, NH); ^13^C{^1^H} NMR (75.4 MHz, DMSO): *δ* 13.7 (CH_3_), 26.9 (CH_2_S), 38.9 (CH), 50.8 (CH_2_N), 55.0 (OCH_3_), 82.4 (C=**C**–CO), 113.7 (Ar), 124.0 (C–NO_2_), 127.8, 128.3, 129.0, 133.7, 136.3, 136.8 (Ar), 141.5 (C=**C**–S), 149.0 (C=N), 150.0 (C–NH_2_), 158.2 (C_Ar_–OMe), 165.6 (C=O); *m*/*z* (%) = 474 (0.1), 439,^2^ 353,^2^ 305,^18^ 288 (100), 257 (94), 218,^17^ 186,^24^ 167 (31), 138 (79), 117,^10^ 103 (77), 77 (43), 61 (38); anal. calcd for C_23_H_22_ClN_5_O_4_S: C, 55.25; H, 4.44; N, 14.01.

#### (*E*)-5-Amino-*N*'-(1-(4-chlorophenyl)ethylidene)-7-(3,4-dimethoxyphenyl)-8-nitro-3,7-dihydro-2*H*-thiazolo[3,2-*a*]pyridine-6-carbohydrazide (6e)

Yellow solid; yield: 0.397 g (75%); mp: 217–219 °C; ^1^H NMR (300 MHz, DMSO): *δ* 2.10 (3H, s, CH_3_), 3.67 (3H, s, OCH_3_), 3.68 (3H, s, OCH_3_), 4.24–4.36 (4H, m, 2CH_2_), 5.51 (1H, s, CH), 6.85 (2H, s, ArH), 7.00 (1H, s, ArH), 7.43 (2H, d, *J* = 8.4 Hz, ArH), 7.75 (2H, d, *J* = 8.4 Hz, ArH), 8.13 (2H, s, NH_2_), 9.14 (1H, s, NH); anal. calcd for C_24_H_24_ClN_5_O_5_S: C, 54.39; H, 4.56; N, 13.21.

#### (*E*)-5-Amino-*N*'-(1-(4-chlorophenyl)ethylidene)-8-nitro-7-phenyl-3,7-dihydro-2*H*-thiazolo[3,2-*a*]pyridine-6-carbohydrazide (6f)

Dark yellow solid; yield: 0.393 g (84%); mp: 248–250 °C; ^1^H NMR (300 MHz, DMSO): *δ* 2.10 (3H, s, CH_3_), 4.21–4.31 (2H, m, CH_2_), 4.36–4.44 (2H, m, CH_2_), 5.59 (1H, s, CH), 7.17–7.41 (5H, m, ArH), 7.42 (2H, d, *J* = 8.7 Hz, ArH), 7.74 (2H, d, *J* = 8.7 Hz, ArH), 8.13 (2H, s, NH_2_), 9.25 (1H, s, NH); anal. calcd for C_22_H_20_ClN_5_O_3_S: C, 56.23; H, 4.29; N, 14.90.

#### (*E*)-5-Amino-7-(3-chlorophenyl)-*N*'-(1-(4-chlorophenyl)ethylidene)-8-nitro-3,7-dihydro-2*H*-thiazolo[3,2-*a*]pyridine-6-carbohydrazide (6g)

Orange solid; yield: 0.413 g (82%); mp: 237–240 °C; ^1^H NMR (300 MHz, DMSO): *δ* 2.11 (3H, s, CH_3_), 4.20–4.30 (2H, m, CH_2_), 4.36–4.42 (2H, m, CH_2_), 5.71 (1H, s, CH), 7.22–7.77 (8H, m, ArH), 8.16 (2H, s, NH_2_), 9.34 (1H, s, NH); anal. calcd for C_22_H_19_Cl_2_N_5_O_3_S: C, 52.39; H, 3.80; N, 13.88.

#### (*E*)-5-Amino-*N*'-(1-(4-chlorophenyl)ethylidene)-7-(3-fluorophenyl)-8-nitro-3,7-dihydro-2*H*-thiazolo[3,2-*a*]pyridine-6-carbohydrazide (6h)

Yellow solid; yield: 0.379 g (78%); mp: 225–227 °C; ^1^H NMR (300 MHz, DMSO): *δ* 2.11 (3H, s, CH_3_), 4.20–4.30 (2H, m, CH_2_), 4.36–4.42 (2H, m, CH_2_), 5.72 (1H, s, CH), 6.95–7.74 (8H, m, ArH), 8.15 (2H, s, NH_2_), 9.34 (1H, s, NH); anal. calcd for C_22_H_19_ClFN_5_O_3_S: C, 54.15; H, 3.92; N, 14.35.

#### (*E*)-5-Amino-7-(3,4-dimethoxyphenyl)-8-nitro-*N*'-(1-(4-nitrophenyl)ethylidene)-3,7-dihydro-2*H*-thiazolo[3,2-*a*]pyridine-6-carbohydrazide (6i)

Light yellow solid; yield: 0.432 g (80%); mp: 213–215 °C; ^1^H NMR (300 MHz, DMSO): *δ* 2.17 (3H, s, CH_3_), 3.68 (3H, s, OCH_3_), 3.70 (3H, s, OCH_3_), 4.22–4.35 (2H, m, CH_2_), 4.36–4.45 (2H, m, CH_2_), 5.55 (1H, s, CH), 6.86 (2H, s, ArH), 7.00 (1H, s, ArH), 7.98 (2H, d, *J* = 9 Hz, ArH), 8.22 (2H, d, *J* = 9 Hz, ArH), 8.24 (2H, s, NH_2_), 9.30 (1H, s, NH); ^13^C{^1^H} NMR (125.6 MHz, DMSO): *δ* 14.1 (CH_3_), 28.1 (CH_2_S), 31.1 (CH), 51.2 (CH_2_N), 55.9 (OCH_3_), 55.9 (OCH_3_), 82.5 (C=**C**–CO), 112.5, 112.6, 120.4 (Ar), 123.9 (C–NO_2_), 124.0, 127.4, 137.2 (Ar), 144.9 (C=**C**–S), 147.6 (C=N), 148.3 (C_Ar_–OMe), 148.6 (C_Ar_–OMe), 150.8 (C–NH_2_), 156.7 (C=O); anal. calcd for C_24_H_24_N_6_O_7_S: C, 53.33; H, 4.48; N, 15.55.

#### (*E*)-5-Amino-7-(3-methoxyphenyl)-8-nitro-*N*'-(1-(4-nitrophenyl)ethylidene)-3,7-dihydro-2*H*-thiazolo[3,2-*a*]pyridine-6-carbohydrazide (6j)

Yellowish orange solid; yield: 0.397 g (78%); mp: 234–236 °C; ^1^H NMR (300 MHz, DMSO): *δ* 2.19 (3H, s, CH_3_), 3.70 (3H, s, OCH_3_), 4.22–4.28 (2H, m, CH_2_), 4.37–4.42 (2H, m, CH_2_), 5.61 (1H, s, CH), 6.77 (1H, d, *J* = 7.8 Hz, ArH), 6.92 (2H, m, ArH), 7.21 (1H, t, *J* = 7.8 Hz, ArH), 7.98 (2H, d, *J* = 9 Hz, ArH), 8.22 (2H, d, *J* = 9 Hz, ArH), 8.24 (2H, s, NH_2_), 9.39 (1H, s, NH); ^13^C{^1^H} NMR (125.6 MHz, DMSO): *δ* 13.6 (CH_3_), 27.6 (CH_2_S), 38.1 (CH), 50.7 (CH_2_N), 54.9 (OCH_3_), 81.9 (C=**C**–CO), 111.5, 114.3, 120.0 (Ar), 123.4 (C–NO_2_), 127.0, 129.4, 131.6, 144.4 (Ar), 145.8 (C=**C**–S), 147.1 (C=N), 150.3 (C–NH_2_), 156.6 (C_Ar_–OMe), 159.0 (C=O); anal. calcd for C_23_H_22_N_6_O_6_S: C, 54.11; H, 4.34; N, 16.46.

#### (*E*)-5-Amino-7-(3-chlorophenyl)-8-nitro-*N*'-(1-(4-nitrophenyl)ethylidene)-3,7-dihydro-2*H*-thiazolo[3,2-*a*]pyridine-6-carbohydrazide (6k)

Orange solid; yield: 0.462 g (90%); mp: 218–220 °C; IR (KBr) (*ν*_max_/cm^−1^): 3408, 3297, 1639, 1573, 1508, 1447, 1387, 1241, 1134, 853, 772; ^1^H NMR (300 MHz, DMSO): *δ* 2.21 (3H, s, CH_3_), 4.22–4.31 (2H, m, CH_2_), 4.35–4.45 (2H, m, CH_2_), 5.71 (1H, s, CH), 7.25–7.42 (4H, m, ArH), 7.99 (2H, d, *J* = 9 Hz, ArH), 8.22 (2H, d, *J* = 9 Hz, ArH), 8.28 (2H, s, NH_2_), 9.49 (1H, s, NH); ^13^C{^1^H} NMR (125.6 MHz, DMSO): *δ* 13.7 (CH_3_), 27.6 (CH_2_S), 37.9 (CH), 50.8 (CH_2_N), 81.4 (C=**C**–CO), 123.0 (C–NO_2_), 123.4, 126.6, 126.8, 127.0, 127.7, 130.2, 132.6 (Ar), 144.4 (C=**C**–S), 146.7, 147.2 (Ar), 148.1 (C=N), 150.3 (C_Ar_–NO_2_), 157.0 (C–NH_2_), 165.7 (C=O); *m*/*z* (%) = 509 (0.02), 471 (0.5), 432 (0.2), 387 (0.1), 326 (48), 311 (100), 292 (78), 261 (59), 222,^25^ 179 (46), 149 (42), 117 (95), 103 (41), 77 (70), 61 (78); anal. calcd for C_22_H_19_ClN_6_O_5_S: C, 51.31; H, 3.72; N, 16.32.

#### (*E*)-5-Amino-7-(4-chlorophenyl)-8-nitro-*N*'-(1-(4-nitrophenyl)ethylidene)-3,7-dihydro-2*H*-thiazolo[3,2-*a*]pyridine-6-carbohydrazide (6l)

Light orange solid; yield: 0.488 g (95%); mp: 244–246 °C; IR (KBr) (*ν*_max_/cm^−1^): 3278, 3147, 1632, 1585, 1516, 1454, 1306, 1238, 1134, 851, 749; ^1^H NMR (300 MHz, DMSO): *δ* 2.21 (3H, s, CH_3_), 4.25–4.32 (2H, m, CH_2_), 4.36–4.42 (2H, m, CH_2_), 5.70 (1H, s, CH), 7.33 (2H, d, *J* = 8.4 Hz, ArH), 7.38 (2H, d, *J* = 8.7 Hz, ArH), 7.98 (2H, d, *J* = 9 Hz, ArH), 8.20 (2H, d, *J* = 9 Hz, ArH), 8.24 (2H, s, NH_2_), 9.50 (1H, s, NH); ^13^C{^1^H} NMR (75.4 MHz, DMSO): *δ* 14.2 (CH_3_), 28.1 (CH_2_S), 38.0 (CH), 50.9 (CH_2_N), 81.8 (C=**C**–CO), 123.9 (C–NO_2_), 127.5, 128.6, 130.1, 139.4, 143.8, 144.9 (C=**C**–S), 147.6, 147.9 (Ar), 148.5 (C=N), 151.0 (C_Ar_–NO_2_), 157.7 (C–NH_2_), 166.5 (C=O); *m*/*z* (%) = 509 (0.01), 453 (0.4), 410 (0.2), 368 (0.8), 326,^18^ 311 (46), 292 (100), 261 (69), 222,^20^ 205,^10^ 179 (40), 149 (55), 117 (38), 103 (53), 77 (90), 61 (70); anal. calcd for C_22_H_19_ClN_6_O_5_S: C, 51.31; H, 3.72; N, 16.32.

#### (*E*)-5-Amino-7-(3-fluorophenyl)-8-nitro-*N*'-(1-(4-nitrophenyl)ethylidene)-3,7-dihydro-2*H*-thiazolo[3,2-*a*]pyridine-6-carbohydrazide (6m)

Yellow solid; yield: 0.423 g (85%); mp: 242–245 °C; IR (KBr) (*ν*_max_/cm^−1^): 3279, 3145, 1630, 1581, 1516, 1441, 1301, 1235, 1184, 1009, 851, 750; ^1^H NMR (300 MHz, DMSO): *δ* 2.21 (3H, s, CH_3_), 4.22–4.31 (2H, m, CH_2_), 4.35–4.45 (2H, m, CH_2_), 5.71 (1H, s, CH), 7.25–7.42 (4H, m, ArH), 7.99 (2H, d, *J* = 9 Hz, ArH), 8.22 (2H, d, *J* = 9 Hz, ArH), 8.28 (2H, s, NH_2_), 9.49 (1H, s, NH); ^13^C{^1^H} NMR (125.6 MHz, DMSO): *δ* 13.7 (CH_3_), 27.7 (CH_2_S), 37.9 (CH), 50.8 (CH_2_N), 81.6 (C=C–CO), 113.7 (d, ^2^*J*_CF_ = 21 Hz, CH of Ar), 114.6 (d, ^2^*J*_CF_ = 21 Hz, CH of Ar), 123.2 (C–NO_2_), 123.5, 124.0, 127.0 (Ar), 130.1 (d, ^3^*J*_CF_ = 8 Hz, CH of Ar), 144.4 (C=**C**–S), 147.2, 147.2 (Ar), 148.1 (C=N), 150.4 (C_Ar_–NO_2_), 157.0 (C–NH_2_), 162.0 (d, ^1^*J*_CF_ = 242 Hz, C–F), 165.8 (C=O); *m*/*z* (%) = 495 (0.02), 455 (0.4), 438 (0.2), 394 (0.1), 351 (0.5), 326,^7^ 311,^12^ 293,^19^ 276 (34), 245 (36), 222,^3^ 206,^15^ 179,^17^ 149 (38), 117 (40), 103 (47), 77 (100), 61 (97); anal. calcd for C_22_H_19_FN_6_O_5_S: C, 53.01; H, 3.84; N, 16.86.

#### (*E*)-5-Amino-8-nitro-*N*'-(1-(4-nitrophenyl)ethylidene)-7-phenyl-3,7-dihydro-2*H*-thiazolo[3,2-*a*]pyridine-6-carbohydrazide (6n)

Yellow solid; yield: 0.417 g (87%); mp: 244–246 °C; ^1^H NMR (300 MHz, DMSO): *δ* 2.19 (3H, s, CH_3_), 4.22–4.32 (2H, m, CH_2_), 4.37–4.43 (2H, m, CH_2_), 5.63 (1H, s, CH), 7.15–7.39 (5H, m, ArH), 7.98 (2H, d, *J* = 9 Hz, ArH), 8.22 (2H, d, *J* = 9 Hz, ArH), 8.24 (2H, s, NH_2_), 9.42 (1H, s, NH); ^13^C{^1^H} NMR (125.6 MHz, DMSO): *δ* 13.6 (CH_3_), 27.6 (CH_2_S), 38.2 (CH), 50.7 (CH_2_N), 82.1 (C=**C**–CO), 123.4 (C–NO_2_), 123.8, 126.8, 126.9, 127.8, 128.2, 144.3 (Ar), 144.4 (C=**C**–S), 147.1 (Ar), 147.4 (C=N), 150.3 (C_Ar_–NO_2_), 156.5 (C–NH_2_), 165.6 (C=O); *m*/*z* (%) = 480 (0.03) [M]^+^, 437,^1^ 392 (0.2), 345,^1^ 326,^16^ 311,^27^ 275 (54), 258 (100), 227 (80), 179,^22^ 149 (41), 117 (30), 103 (48), 77 (71), 61 (45); anal. calcd for C_22_H_20_N_6_O_5_S: C, 54.99; H, 4.20; N, 17.49.

#### (*E*)-5-Amino-*N*'-(1-(4-bromophenyl)ethylidene)-7-(4-methoxyphenyl)-8-nitro-3,7-dihydro-2*H*-thiazolo[3,2-*a*]pyridine-6-carbohydrazide (6o)

Yellow solid; yield: 0.408 g (75%); mp: 253–255 °C; ^1^H NMR (300 MHz, DMSO): *δ* 2.09 (3H, s, CH_3_), 3.68 (3H, s, OCH_3_), 4.23–4.28 (2H, m, CH_2_), 4.35–4.39 (2H, m, CH_2_), 5.50 (1H, s, CH), 6.82 (2H, d, *J* = 8.1 Hz, ArH), 7.27 (2H, d, *J* = 8.1 Hz, ArH), 7.56 (2H, d, *J* = 8.1 Hz, ArH), 7.67 (2H, d, *J* = 8.4 Hz, ArH), 8.11 (2H, s, NH_2_), 9.16 (1H, s, NH); anal. calcd for C_23_H_22_BrN_5_O_4_S: C, 50.74; H, 4.07; N, 12.86.

#### (*E*)-5-Amino-*N*'-(1-(4-bromophenyl)ethylidene)-7-(4-chlorophenyl)-8-nitro-3,7-dihydro-2*H*-thiazolo[3,2-*a*]pyridine-6-carbohydrazide (6p)

Yellow solid; yield: 0.383 g (70%); mp: 262–264 °C; ^1^H NMR (300 MHz, DMSO): *δ* 2.10 (3H, s, CH_3_), 4.24–4.29 (2H, m, CH_2_), 4.32–4.38 (2H, m, CH_2_), 5.65 (1H, s, CH), 7.34–7.37 (4H, m, ArH), 7.56 (2H, d, *J* = 8.1 Hz, ArH), 7.66 (2H, d, *J* = 9 Hz, ArH), 8.14 (2H, s, NH_2_), 9.34 (1H, s, NH); anal. calcd for C_22_H_19_BrClN_5_O_3_S: C, 48.14; H, 3.49; N, 12.76. Found: C, 48.5; H, 3.2; N, 12.6.

## Conflicts of interest

The authors declare no competing financial interest.

## Supplementary Material

RA-010-D0RA03910A-s001
