# Supplementary material for: Synthesis of highly functionalized thiazolo[3,2-a]pyridine derivatives via a five-component cascade reaction based on nitroketene N,S-acetal
Source: RSC Adv. 2020 Aug 21;10(52):31039–48. doi: 10.1039/d0ra03910a (PMC9056361; doi:10.1039/d0ra03910a)
Supplement: RA-010-D0RA03910A-s001 [file RA-010-D0RA03910A-s001.pdf]

Electronic Supplementary Material (ESI) for RSC Advances.
This journal is © The Royal Society of Chemistry 2020

Supporting Information

Synthesis of highly functionalized thiazolo[3,2-*a*]pyridine derivatives *via* a five-component cascade reaction based on nitroketene *N,S*-acetal

Zohreh Sahhaf Razavi, Mohammad Bayat*, Hajar Hosseini

The Table of Contents

Title	Page
Title, author's name, address and table of contents	1
Experimental Section; General remarks	2
**Figure 1.** Structure of all products **6a-p**	3
^{1}H and ^{13}C NMR and IR spectrums of **6a**	5-7
^{1}H and ^{13}C NMR spectrums of **6b**	8-9
^{1}H and ^{13}C NMR spectrums of **6c**	10-11
^{1}H and ^{13}C NMR and IR and Mass spectrums of **6d**	12-15
^{1}H spectrum of **6e**	16
^{1}H spectrum of **6f**	17
^{1}H spectrum of **6g**	18
^{1}H spectrum of **6h**	19
^{1}H and ^{13}C NMR spectrums of **6i**	20-21
^{1}H and ^{13}C NMR spectrums of **6j**	22-23
^{1}H and ^{13}C NMR and IR and Mass spectrums of **6k**	24-27
^{1}H and ^{13}C NMR and IR and Mass spectrums of **6l**	28-31
^{1}H and ^{13}C NMR and IR and Mass spectrums of **6m**	32-35
^{1}H and ^{13}C NMR and Mass spectrums of **6n**	36-38
^{1}H spectrum of **6o**	39
^{1}H spectrum of **6p**	40

Experimental Section

General remarks:

Melting points were measured on an Electrothermal 9100 apparatus. Mass spectra were recorded with an Agilent 5975C VL MSD with Triple-Axis Detector operating at an ionization potential of 70 eV. ^{1}H and ^{13}C NMR spectra were measured (DMSO) with a Bruker DRX-300 AVANCE spectrometer at 300 and 75 MHz, respectively. IR spectra were recorded on a Bruker Tensor 27, ῡ in cm^{-1}. All NMR spectra at room temperature were determined in DMSO-d_6. Chemical shifts are reported in parts per million (δ) downfield from an internal tetramethylsilane reference. Coupling constants (J values) are reported in hertz (Hz), and spin multiplicities are indicated by the following symbols: s (singlet), d (doublet), t (triplet), q (quartet), m (multiplet). All chemicals were purchased from Merck or Aldrich and were used without further purification.

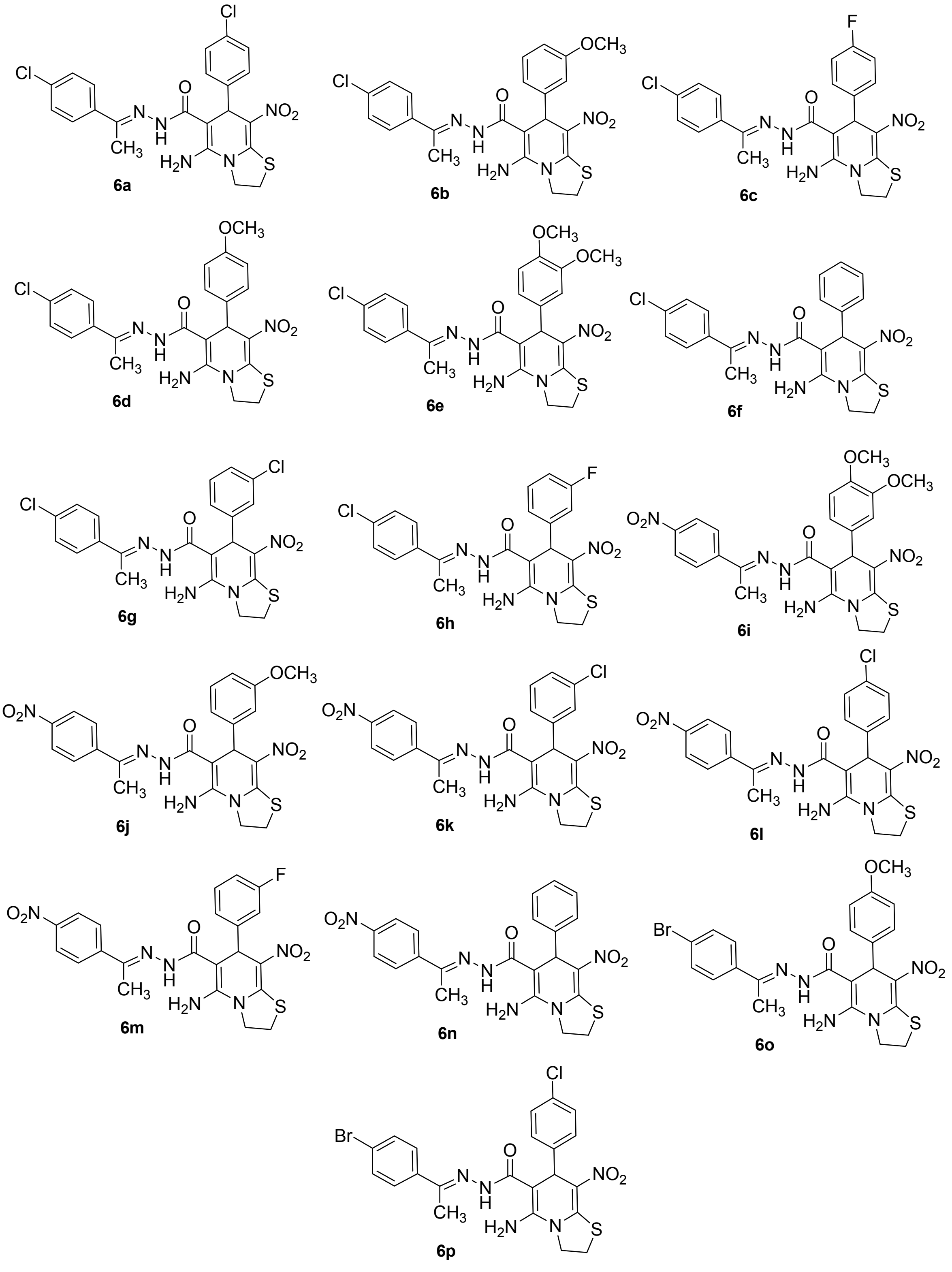

Figure 1. Structure of all products **6a-p.**

The structures of the products **6a-p** were deduced from their IR, mass, ^{1}H NMR, and ^{13}C NMR spectra (see the following images).

The ^{1}H and ^{13}C NMR spectra are taken in DMSO-d_6, but some of the products are slightly soluble in the solvent therefore have no clear carbon spectra such as **6d, 6e, 6f, 6g, 6h, 6l, 6m, 6n.**

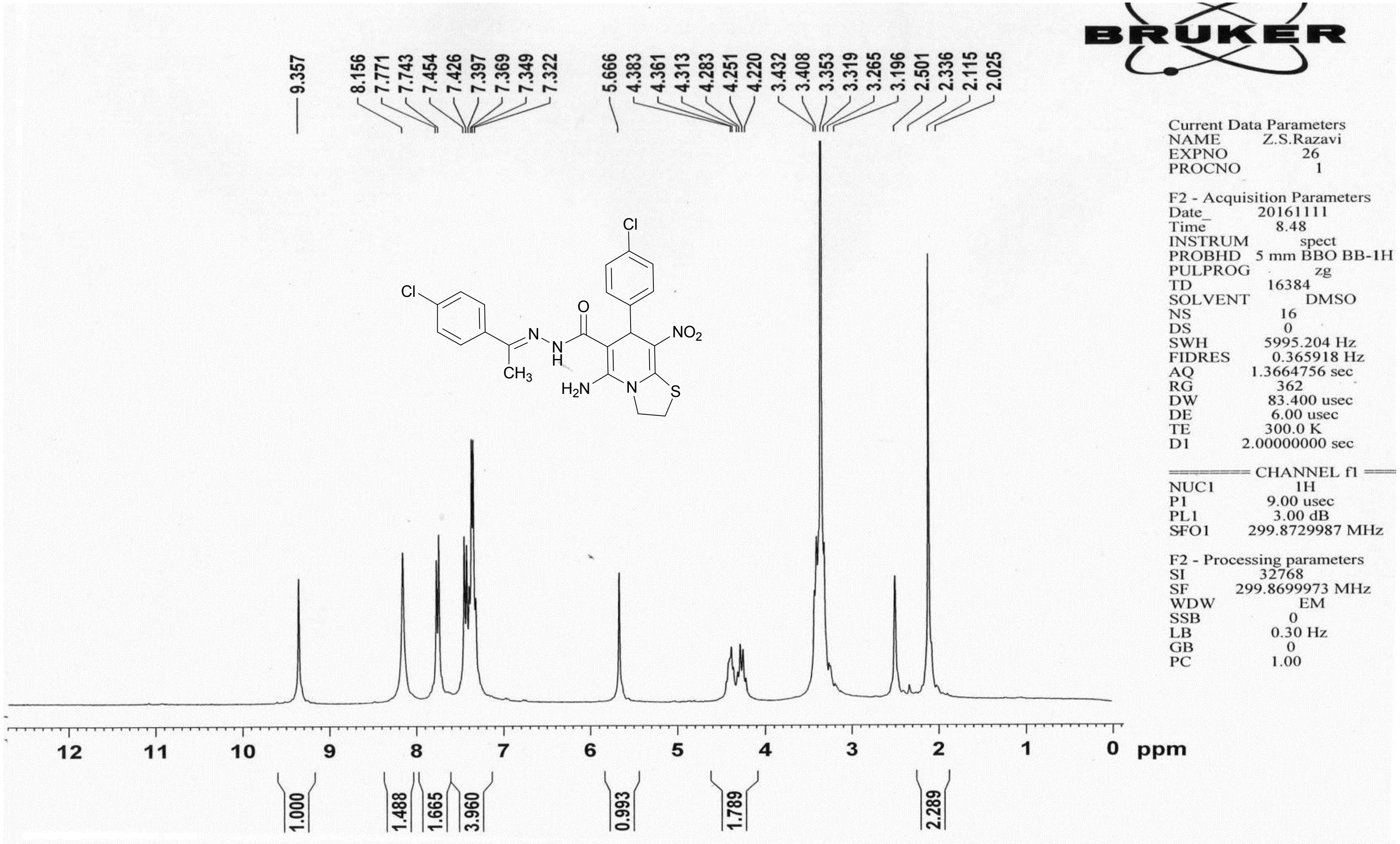
9.357
8.156
7.771
7.743
7.454
7.426
7.397
7.369
7.349
7.322
5.666
4.383
4.361
4.313
4.283
4.251
4.220
3.432
3.408
3.353
3.319
3.265
3.196
2.501
2.336
2.115
2.025
BRUKER
Current Data Parameters
NAME Z.S.Razavi
EXPNO 26
PROCNO 1
F2 - Acquisition Parameters
Date_ 20161111
Time 8.48
INSTRUM spect
PROBHD 5 mm BBO BB-1H
PULPROG zg
TD 16384
SOLVENT DMSO
NS 16
DS 0
SWH 5995.204 Hz
FIDRES 0.365918 Hz
AQ 1.3664756 sec
RG 362
DW 83.400 usec
DE 6.00 usec
TE 300.0 K
D1 2.00000000 sec
CHANNEL f1
NUC1 1H
P1 9.00 usec
PL1 3.00 dB
SFO1 299.8729987 MHz
F2 - Processing parameters
SI 32768
SF 299.8699973 MHz
WDW EM
SSB 0
LB 0.30 Hz
GB 0
PC 1.00
Cl
Cl
O
NO2
N
N
H
CH3
H2N
N
S
12 11 10 9 8 7 6 5 4 3 2 1 0 ppm
1.000
1.488
1.665
3.960
0.993
1.789
2.289

^{1}H NMR of 6a

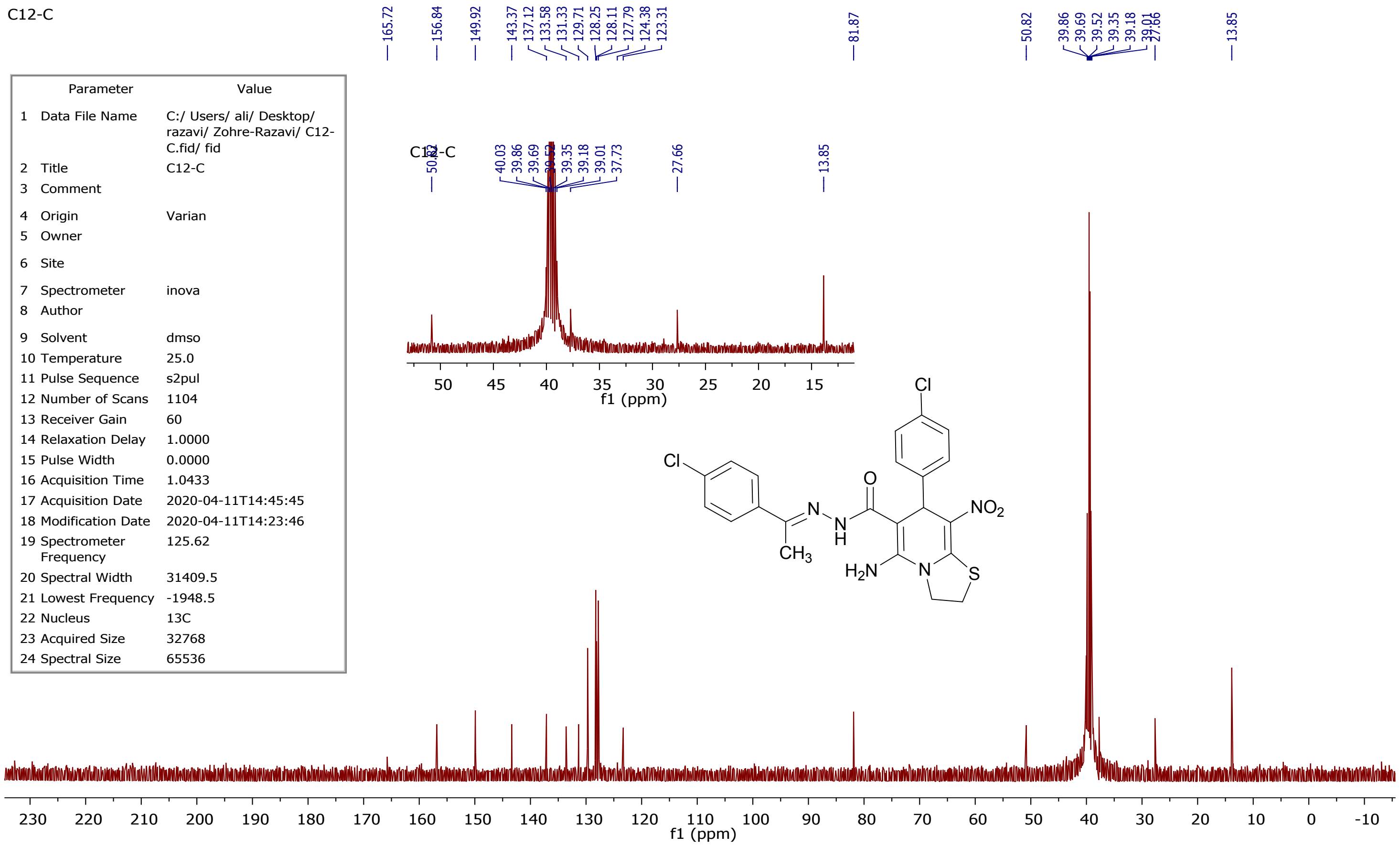

	Parameter	Value
1	Data File Name	C:/ Users/ ali/ Desktop/ razavi/ Zohre-Razavi/ C12-C.fid/ fid
2	Title	C12-C
3	Comment	
4	Origin	Varian
5	Owner	
6	Site	
7	Spectrometer	inova
8	Author	
9	Solvent	dmso
10	Temperature	25.0
11	Pulse Sequence	s2pul
12	Number of Scans	1104
13	Receiver Gain	60
14	Relaxation Delay	1.0000
15	Pulse Width	0.0000
16	Acquisition Time	1.0433
17	Acquisition Date	2020-04-11T14:45:45
18	Modification Date	2020-04-11T14:23:46
19	Spectrometer Frequency	125.62
20	Spectral Width	31409.5
21	Lowest Frequency	-1948.5
22	Nucleus	13C
23	Acquired Size	32768
24	Spectral Size	65536

^{13}C NMR of 6a

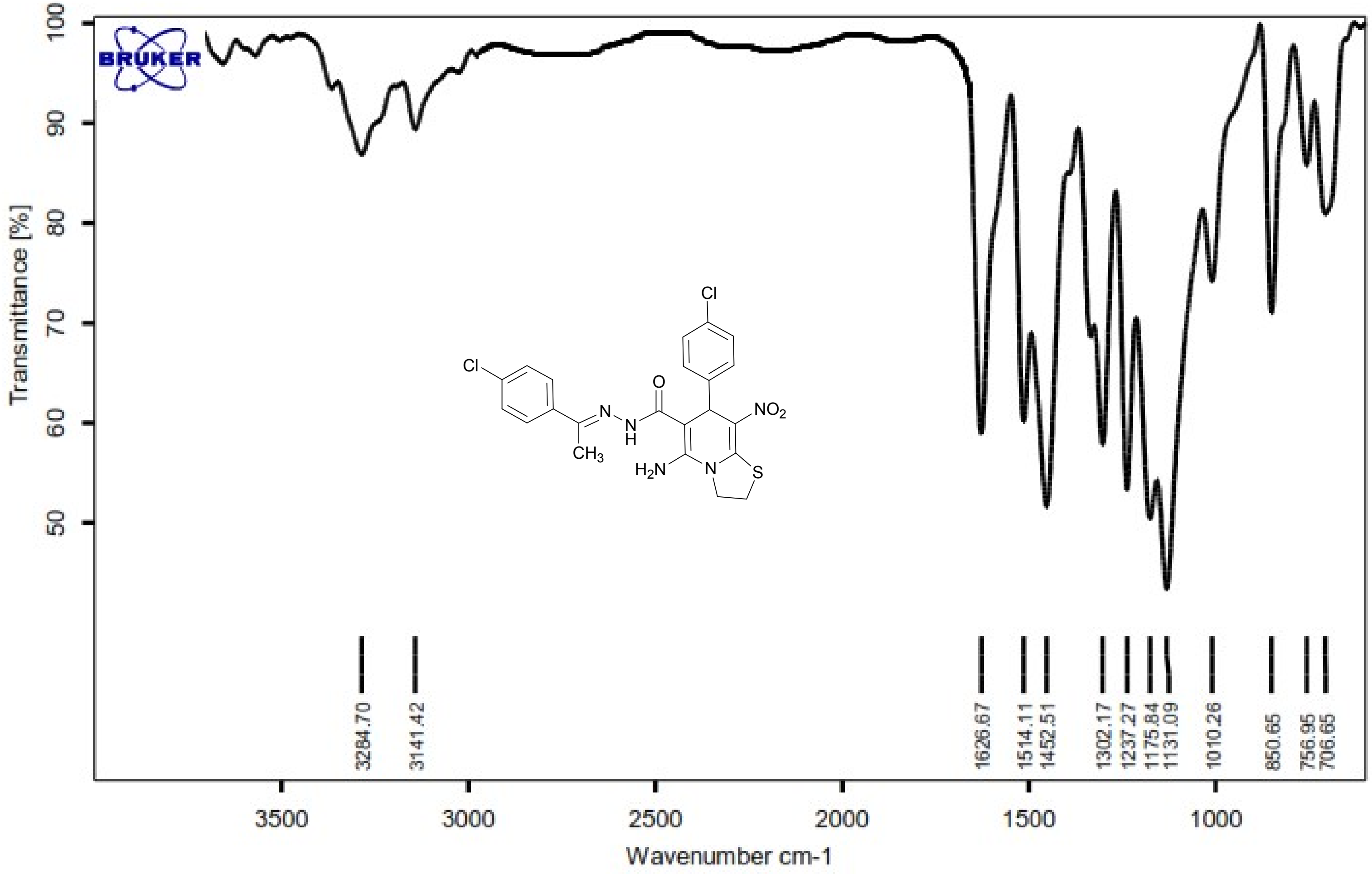

Transmittance [%]
100
90
80
70
60
50
3284.70
3141.42
1626.67
1514.11
1452.51
1302.17
1237.27
1175.84
1131.09
1010.26
850.65
756.95
706.65
3500
3000
2500
2000
1500
1000
Wavenumber cm-1

IR of 6a

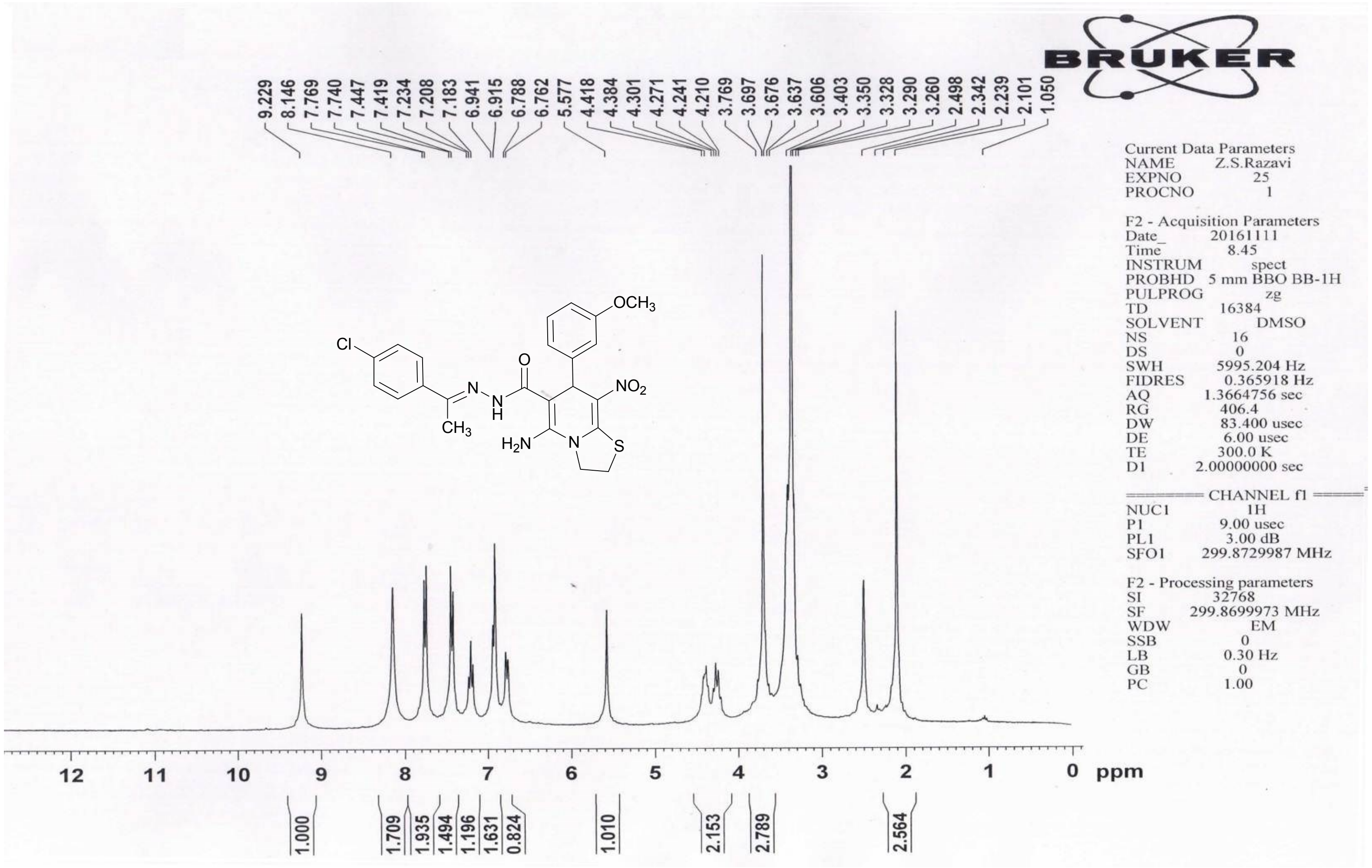
9.229
8.146
7.769
7.740
7.447
7.419
7.234
7.208
7.183
6.941
6.915
6.788
6.762
5.577
4.418
4.384
4.301
4.271
4.241
4.210
3.769
3.697
3.676
3.637
3.606
3.403
3.350
3.328
3.290
3.260
2.498
2.342
2.239
2.101
1.050
BRUKER
Current Data Parameters
NAME Z.S.Razavi
EXPNO 25
PROCNO 1
F2 - Acquisition Parameters
Date_ 20161111
Time 8.45
INSTRUM spect
PROBHD 5 mm BBO BB-1H
PULPROG zg
TD 16384
SOLVENT DMSO
NS 16
DS 0
SWH 5995.204 Hz
FIDRES 0.365918 Hz
AQ 1.3664756 sec
RG 406.4
DW 83.400 usec
DE 6.00 usec
TE 300.0 K
D1 2.00000000 sec
CHANNEL f1
NUC1 1H
P1 9.00 usec
PL1 3.00 dB
SFO1 299.8729987 MHz
F2 - Processing parameters
SI 32768
SF 299.8699973 MHz
WDW EM
SSB 0
LB 0.30 Hz
GB 0
PC 1.00
OCH3
Cl
O
NO2
N
N
H
CH3
H2N
N
S
12
11
10
9
8
7
6
5
4
3
2
1
0
ppm
1.000
1.709
1.935
1.494
1.196
1.631
0.824
1.010
2.153
2.789
2.564

^{1}H NMR of 6b

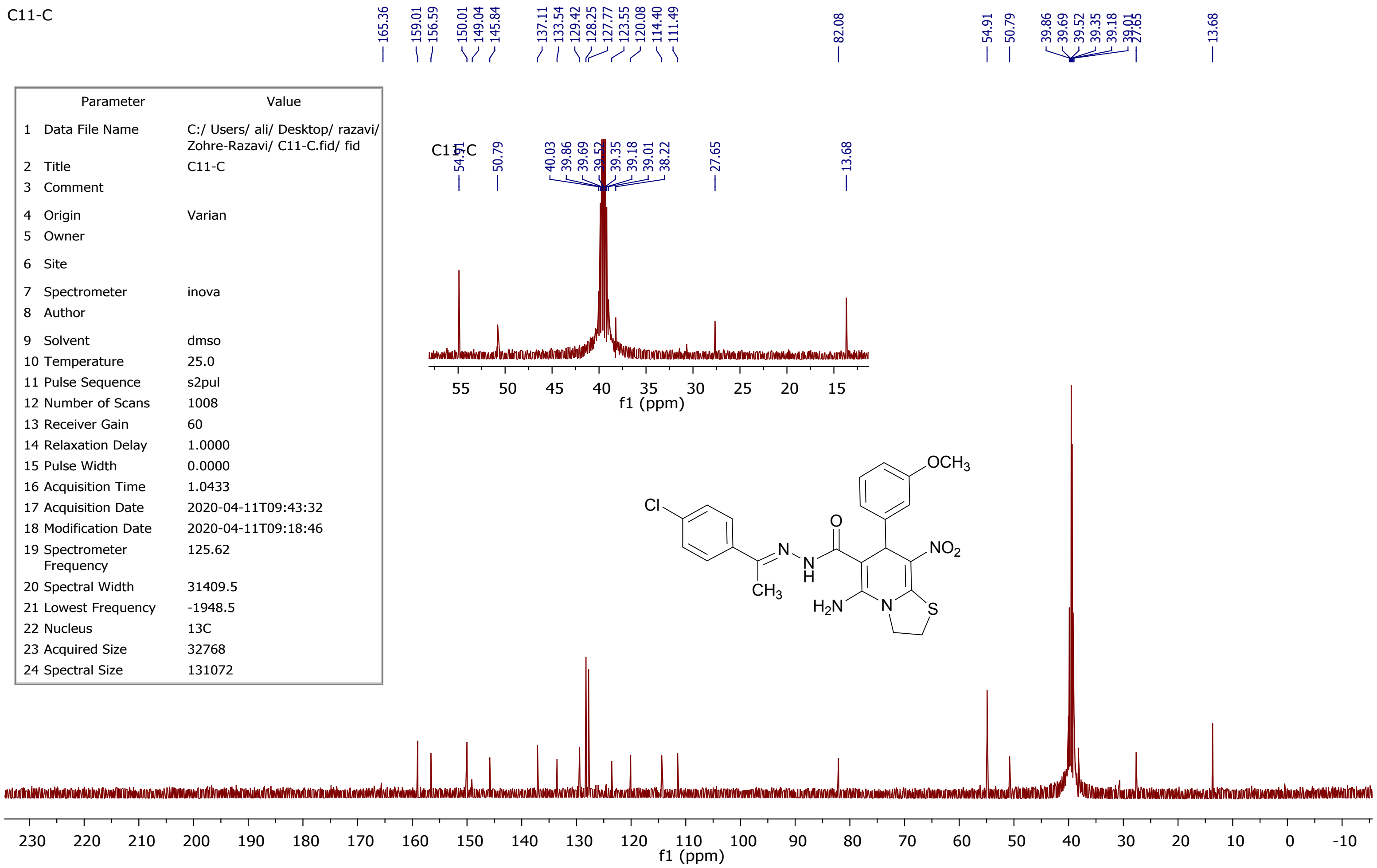
C11-C
165.36
159.01
156.59
150.01
149.04
145.84
137.11
133.54
129.42
128.25
127.77
123.55
120.08
114.40
111.49
82.08
54.91
50.79
39.86
39.69
39.52
39.35
39.18
39.01
27.65
13.68
Parameter Value
1 Data File Name C:/ Users/ ali/ Desktop/ razavi/ Zohre-Razavi/ C11-C.fid/ fid
2 Title C11-C
3 Comment
4 Origin Varian
5 Owner
6 Site
7 Spectrometer inova
8 Author
9 Solvent dmso
10 Temperature 25.0
11 Pulse Sequence s2pul
12 Number of Scans 1008
13 Receiver Gain 60
14 Relaxation Delay 1.0000
15 Pulse Width 0.0000
16 Acquisition Time 1.0433
17 Acquisition Date 2020-04-11T09:43:32
18 Modification Date 2020-04-11T09:18:46
19 Spectrometer Frequency 125.62
20 Spectral Width 31409.5
21 Lowest Frequency -1948.5
22 Nucleus 13C
23 Acquired Size 32768
24 Spectral Size 131072
C11-C
54.91
50.79
40.03
39.86
39.69
39.52
39.35
39.18
39.01
38.22
27.65
13.68
55 50 45 40 35 30 25 20 15
f1 (ppm)
OCH3
Cl
O
N
N
H
NO2
CH3
H2N
N
S
230 220 210 200 190 180 170 160 150 140 130 120 110 100 90 80 70 60 50 40 30 20 10 0 -10
f1 (ppm)

^{13}C NMR of 6b

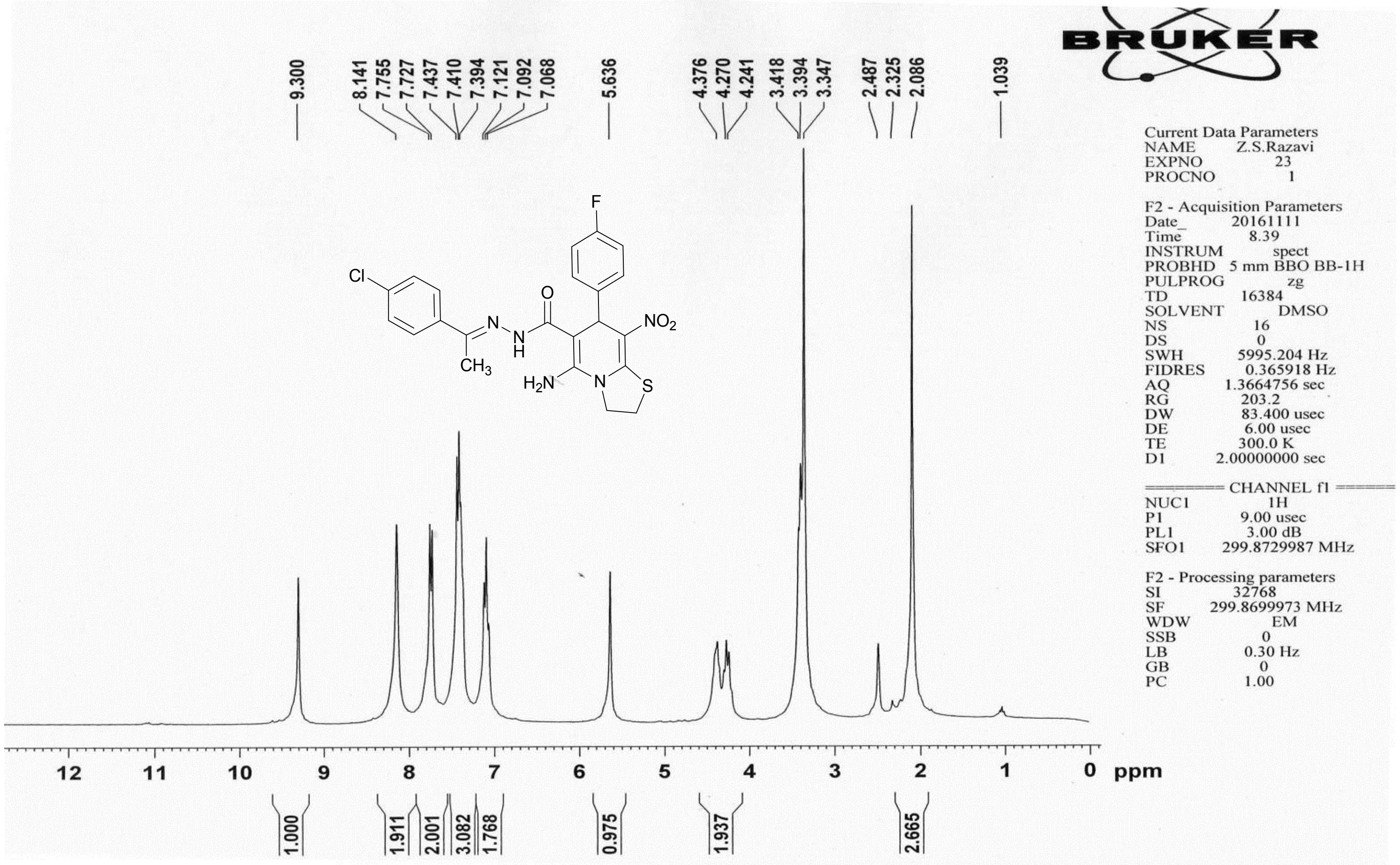

9.300
8.141
7.755
7.727
7.437
7.410
7.394
7.121
7.092
7.068
5.636
4.376
4.270
4.241
3.418
3.394
3.347
2.487
2.325
2.086
1.039
BRUKER
Current Data Parameters
NAME Z.S.Razavi
EXPNO 23
PROCNO 1
F2 - Acquisition Parameters
Date_ 20161111
Time 8.39
INSTRUM spect
PROBHD 5 mm BBO BB-1H
PULPROG zg
TD 16384
SOLVENT DMSO
NS 16
DS 0
SWH 5995.204 Hz
FIDRES 0.365918 Hz
AQ 1.3664756 sec
RG 203.2
DW 83.400 usec
DE 6.00 usec
TE 300.0 K
D1 2.00000000 sec
CHANNEL f1
NUC1 1H
P1 9.00 usec
PL1 3.00 dB
SFO1 299.8729987 MHz
F2 - Processing parameters
SI 32768
SF 299.8699973 MHz
WDW EM
SSB 0
LB 0.30 Hz
GB 0
PC 1.00
ppm
1.000
1.911
2.001
3.082
1.768
0.975
1.937
2.665

^{1}H NMR of 6c

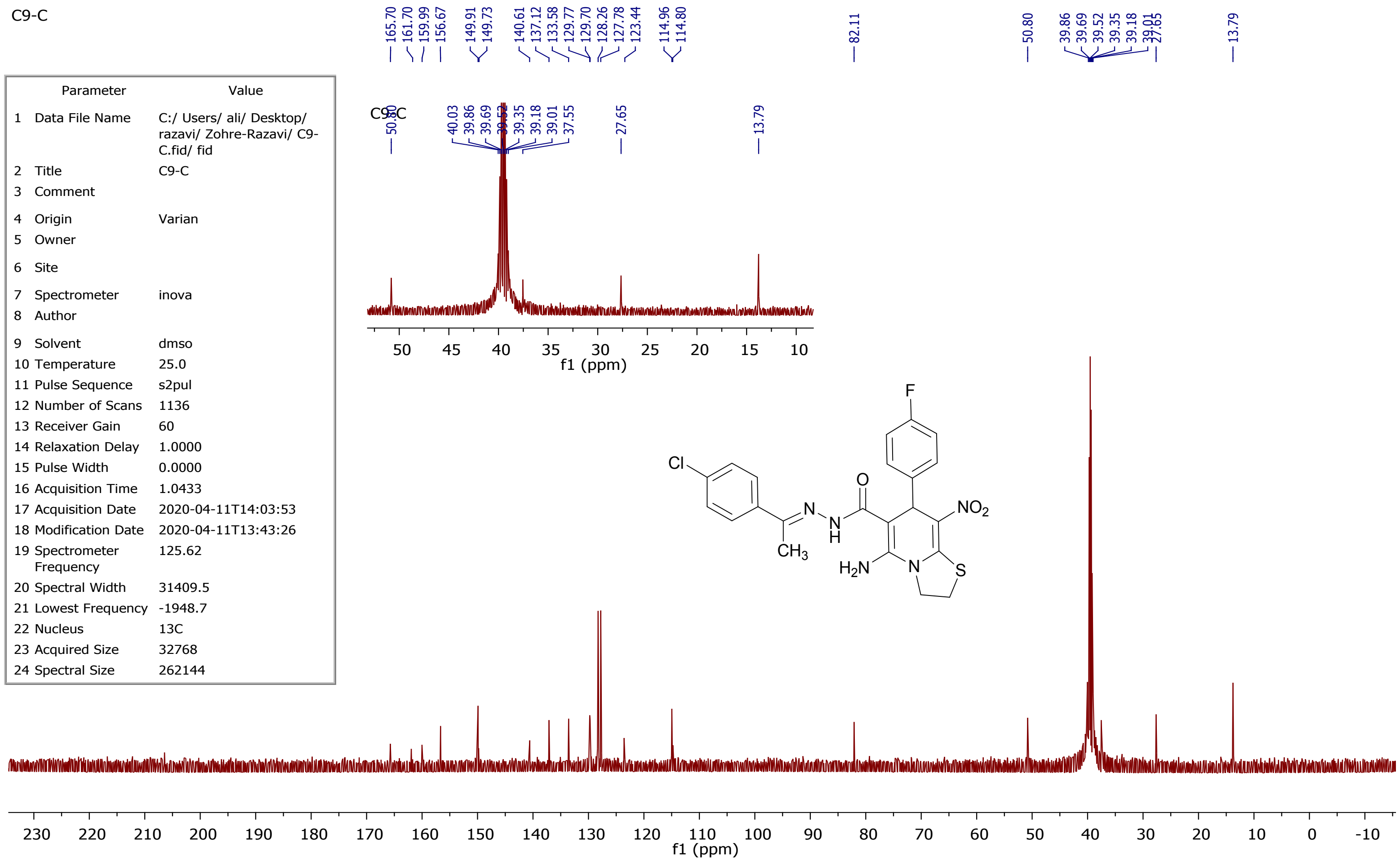

	Parameter	Value
1	Data File Name	C:/ Users/ ali/ Desktop/ razavi/ Zohre-Razavi/ C9-C.fid/ fid
2	Title	C9-C
3	Comment	
4	Origin	Varian
5	Owner	
6	Site	
7	Spectrometer	inova
8	Author	
9	Solvent	dmso
10	Temperature	25.0
11	Pulse Sequence	s2pul
12	Number of Scans	1136
13	Receiver Gain	60
14	Relaxation Delay	1.0000
15	Pulse Width	0.0000
16	Acquisition Time	1.0433
17	Acquisition Date	2020-04-11T14:03:53
18	Modification Date	2020-04-11T13:43:26
19	Spectrometer Frequency	125.62
20	Spectral Width	31409.5
21	Lowest Frequency	-1948.7
22	Nucleus	13C
23	Acquired Size	32768
24	Spectral Size	262144

^{13}C NMR of 6c

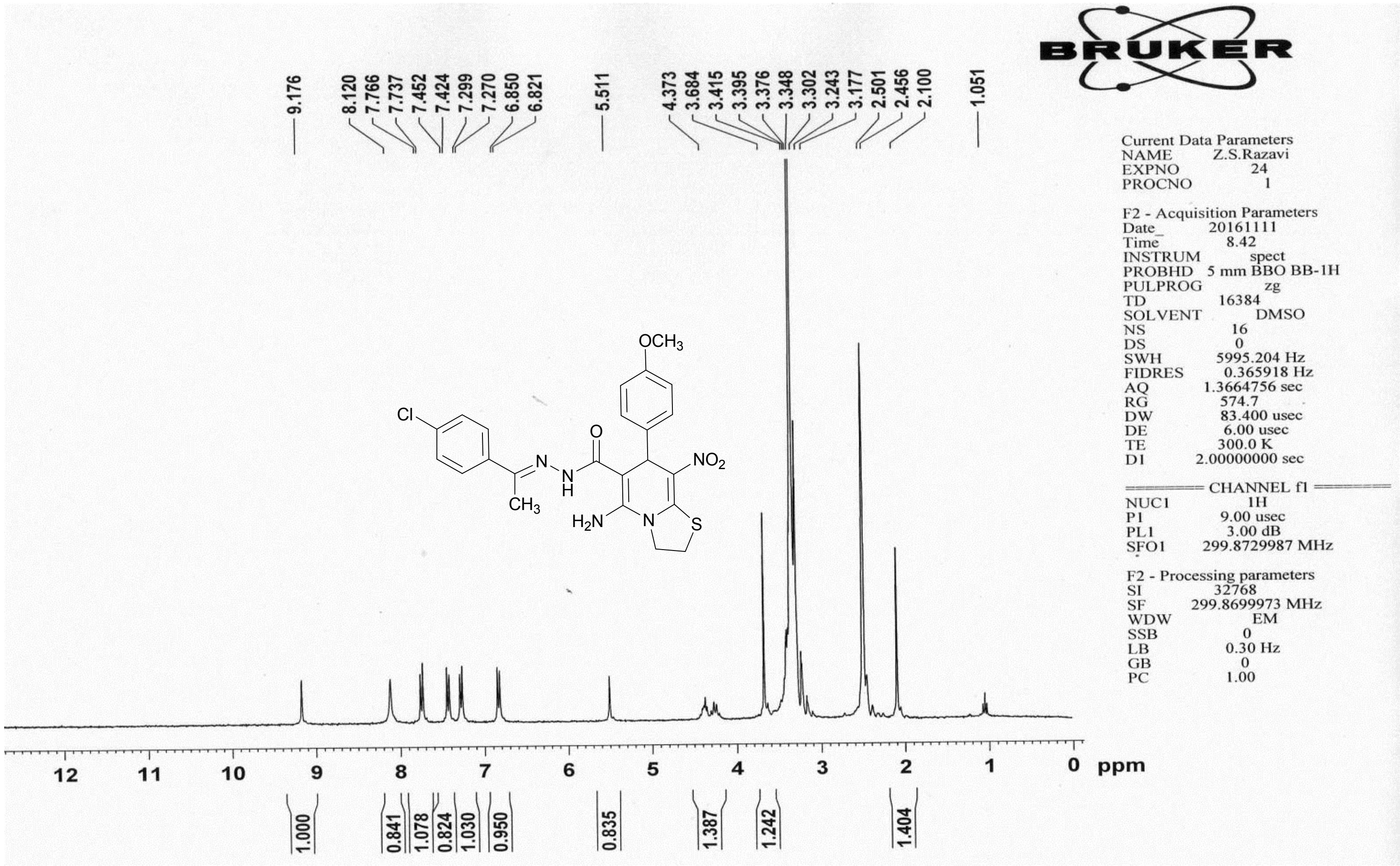
9.176
8.120
7.766
7.737
7.452
7.424
7.299
7.270
6.850
6.821
5.511
4.373
3.684
3.415
3.395
3.376
3.348
3.302
3.243
3.177
2.501
2.456
2.100
1.051
BRUKER
Current Data Parameters
NAME Z.S.Razavi
EXPNO 24
PROCNO 1
F2 - Acquisition Parameters
Date_ 20161111
Time 8.42
INSTRUM spect
PROBHD 5 mm BBO BB-1H
PULPROG zg
TD 16384
SOLVENT DMSO
NS 16
DS 0
SWH 5995.204 Hz
FIDRES 0.365918 Hz
AQ 1.3664756 sec
RG 574.7
DW 83.400 usec
DE 6.00 usec
TE 300.0 K
D1 2.00000000 sec
CHANNEL f1
NUC1 1H
P1 9.00 usec
PL1 3.00 dB
SFO1 299.8729987 MHz
F2 - Processing parameters
SI 32768
SF 299.8699973 MHz
WDW EM
SSB 0
LB 0.30 Hz
GB 0
PC 1.00
OCH3
Cl
O
N
N
H
NO2
CH3
H2N
N
S
12 11 10 9 8 7 6 5 4 3 2 1 0 ppm
1.000
0.841
1.078
0.824
1.030
0.950
0.835
1.387
1.242
1.404

^{1}H NMR of 6d

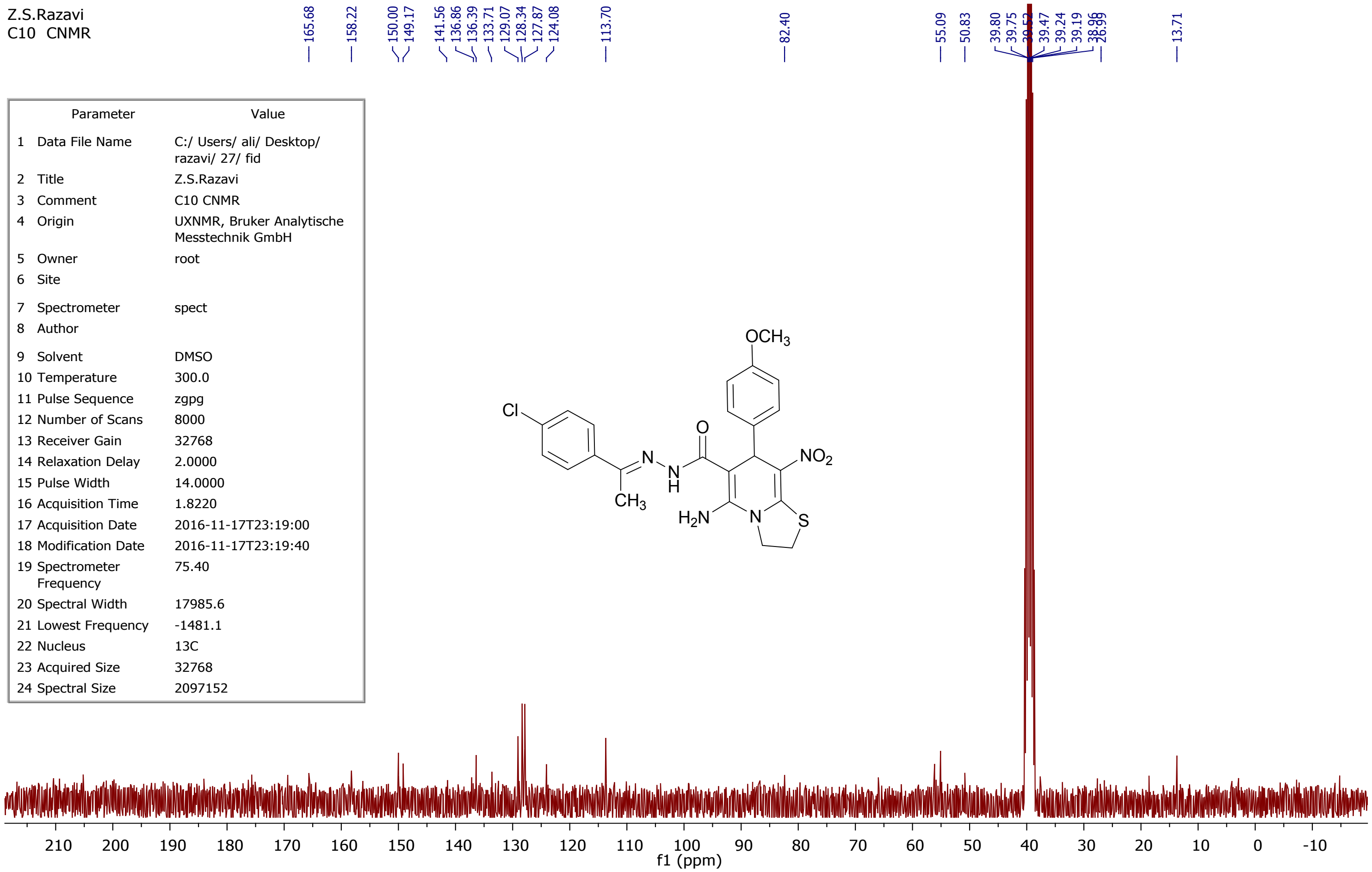
Z.S.Razavi
C10 CNMR
165.68
158.22
150.00
149.17
141.56
136.86
136.39
133.71
129.07
128.34
127.87
124.08
113.70
82.40
55.09
50.83
39.80
39.75
39.47
39.24
39.19
13.71
Parameter Value
1 Data File Name C:/ Users/ ali/ Desktop/ razavi/ 27/ fid
2 Title Z.S.Razavi
3 Comment C10 CNMR
4 Origin UXNMR, Bruker Analytische Messtechnik GmbH
5 Owner root
6 Site
7 Spectrometer spect
8 Author
9 Solvent DMSO
10 Temperature 300.0
11 Pulse Sequence zgpg
12 Number of Scans 8000
13 Receiver Gain 32768
14 Relaxation Delay 2.0000
15 Pulse Width 14.0000
16 Acquisition Time 1.8220
17 Acquisition Date 2016-11-17T23:19:00
18 Modification Date 2016-11-17T23:19:40
19 Spectrometer Frequency 75.40
20 Spectral Width 17985.6
21 Lowest Frequency -1481.1
22 Nucleus 13C
23 Acquired Size 32768
24 Spectral Size 2097152
OCH3
Cl
O
NO2
N
N
H
CH3
H2N
N
S
210 200 190 180 170 160 150 140 130 120 110 100 90 80 70 60 50 40 30 20 10 0 -10
f1 (ppm)

^{13}C NMR of 6d

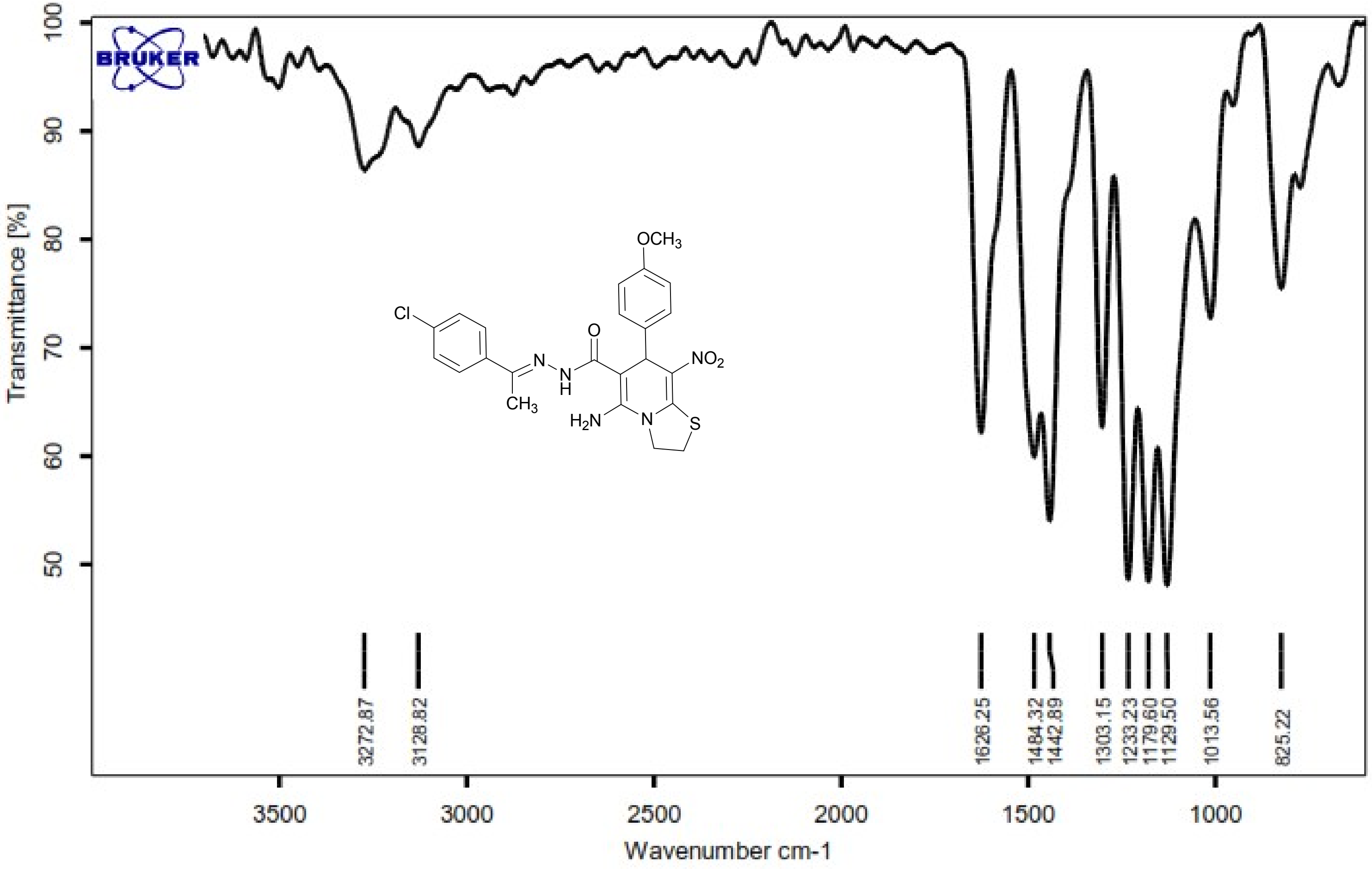

Transmittance [%]
100
90
80
70
60
50
BRUKER
OCH3
Cl
O
N
N
H
NO2
CH3
H2N
N
S
3272.87
3128.82
1626.25
1484.32
1442.89
1303.15
1233.23
1179.60
1129.50
1013.56
825.22
3500
3000
2500
2000
1500
1000
Wavenumber cm-1

IR of 6d

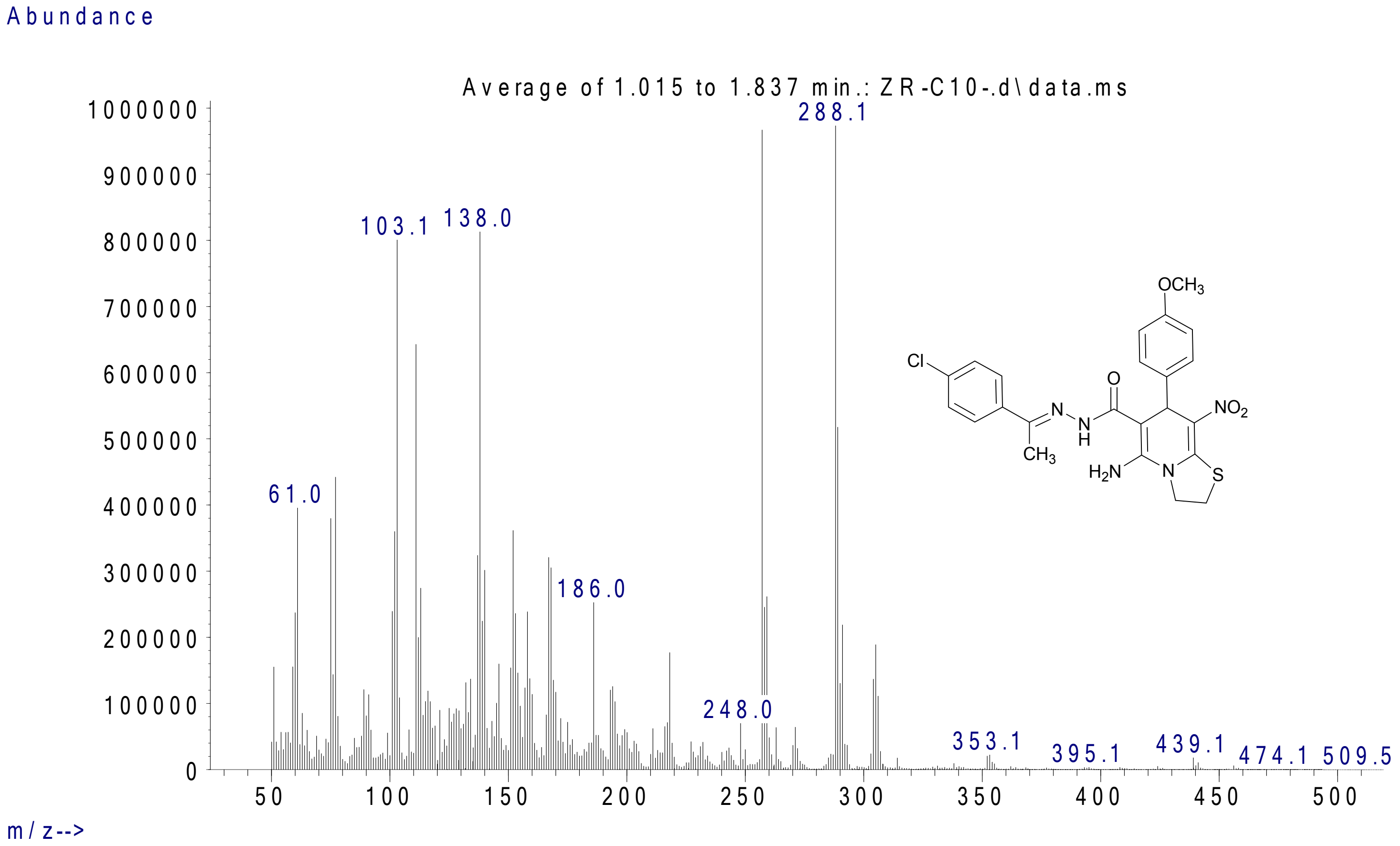
Abundance
Average of 1.015 to 1.837 min.: ZR-C10-.d\data.ms
1000000
900000
800000
700000
600000
500000
400000
300000
200000
100000
0
61.0
103.1
138.0
186.0
248.0
288.1
353.1
395.1
439.1
474.1
509.5
50
100
150
200
250
300
350
400
450
500
m/z-->
OCH3
Cl
O
N
NO2
N
H
CH3
H2N
N
S

MS of 6d

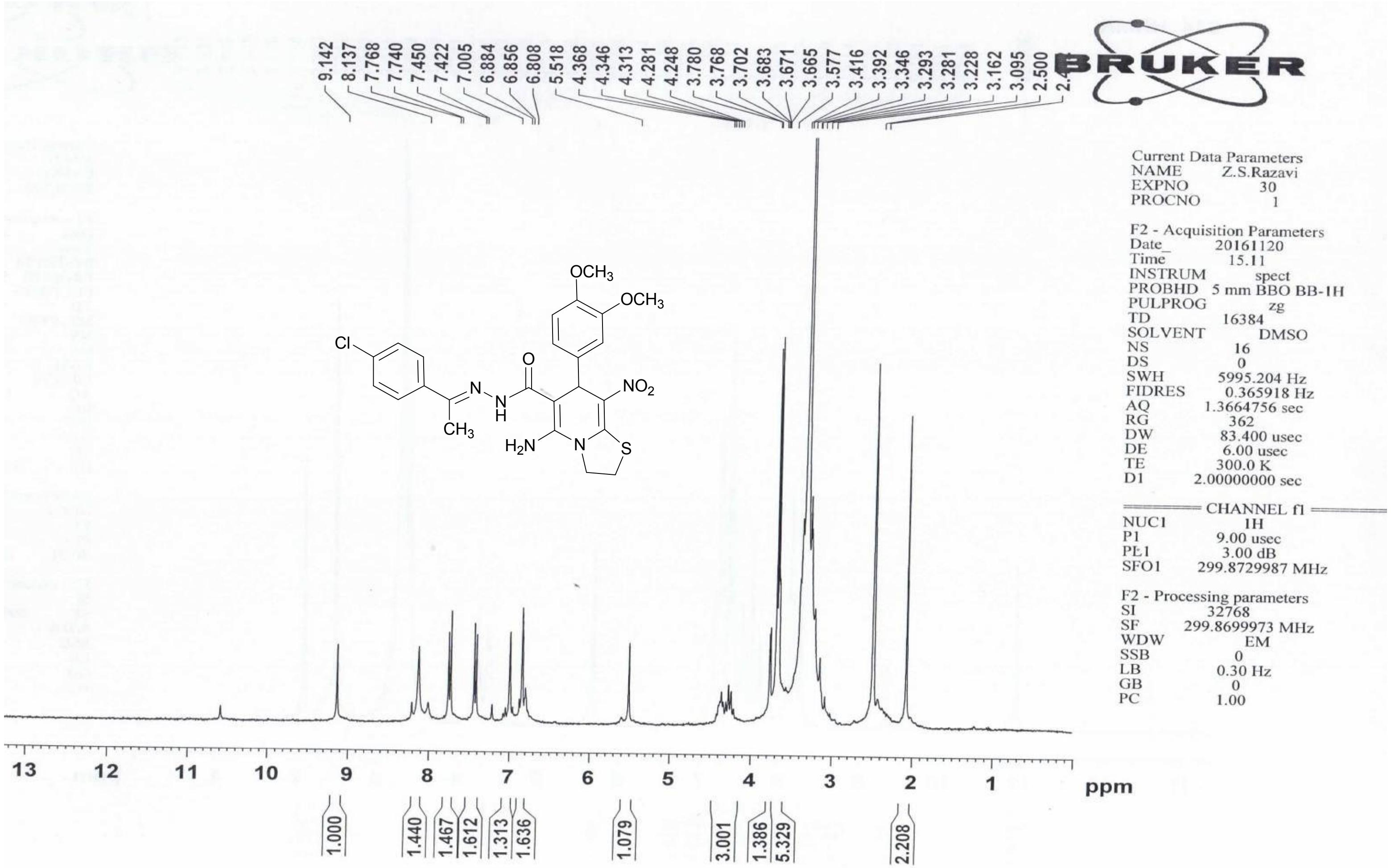
9.142
8.137
7.768
7.740
7.450
7.422
7.005
6.884
6.856
6.808
5.518
4.368
4.346
4.313
4.281
4.248
3.780
3.768
3.702
3.683
3.671
3.665
3.577
3.416
3.392
3.346
3.293
3.281
3.228
3.162
3.095
2.500
BRUKER
Current Data Parameters
NAME Z.S.Razavi
EXPNO 30
PROCNO 1
F2 - Acquisition Parameters
Date_ 20161120
Time 15.11
INSTRUM spect
PROBHD 5 mm BBO BB-1H
PULPROG zg
TD 16384
SOLVENT DMSO
NS 16
DS 0
SWH 5995.204 Hz
FIDRES 0.365918 Hz
AQ 1.3664756 sec
RG 362
DW 83.400 usec
DE 6.00 usec
TE 300.0 K
D1 2.00000000 sec
CHANNEL f1
NUC1 1H
P1 9.00 usec
PL1 3.00 dB
SFO1 299.8729987 MHz
F2 - Processing parameters
SI 32768
SF 299.8699973 MHz
WDW EM
SSB 0
LB 0.30 Hz
GB 0
PC 1.00
OCH3
OCH3
Cl
O
N
N
H
NO2
CH3
H2N
N
S
13 12 11 10 9 8 7 6 5 4 3 2 1 ppm
1.000
1.440
1.467
1.612
1.313
1.636
1.079
3.001
1.386
5.329
2.208

^{1}H NMR of 6e

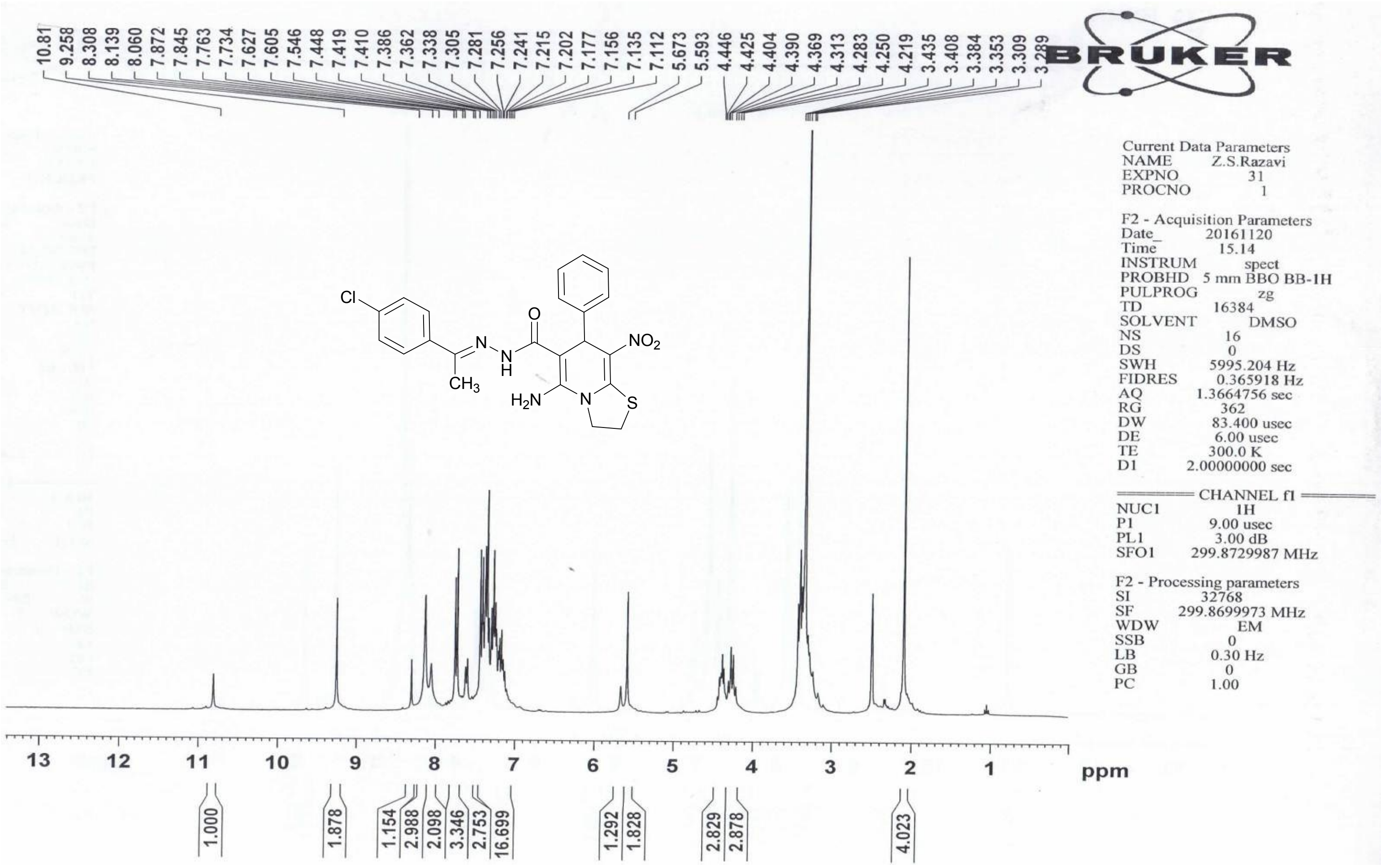

10.81 9.258 8.308 8.139 8.060 7.872 7.845 7.763 7.734 7.627 7.605 7.546 7.448 7.419 7.410 7.386 7.362 7.338 7.305 7.281 7.256 7.241 7.215 7.202 7.177 7.156 7.135 7.112 5.673 5.593 4.446 4.425 4.404 4.390 4.369 4.313 4.283 4.250 4.219 3.435 3.408 3.384 3.353 3.309 3.289
BRUKER
Cl
O
N
N
H
NO2
CH3
H2N
N
S
Current Data Parameters
NAME Z.S.Razavi
EXPNO 31
PROCNO 1
F2 - Acquisition Parameters
Date_ 20161120
Time 15.14
INSTRUM spect
PROBHD 5 mm BBO BB-1H
PULPROG zg
TD 16384
SOLVENT DMSO
NS 16
DS 0
SWH 5995.204 Hz
FIDRES 0.365918 Hz
AQ 1.3664756 sec
RG 362
DW 83.400 usec
DE 6.00 usec
TE 300.0 K
D1 2.00000000 sec
CHANNEL f1
NUC1 1H
P1 9.00 usec
PL1 3.00 dB
SFO1 299.8729987 MHz
F2 - Processing parameters
SI 32768
SF 299.8699973 MHz
WDW EM
SSB 0
LB 0.30 Hz
GB 0
PC 1.00
13 12 11 10 9 8 7 6 5 4 3 2 1 ppm
1.000 1.878 1.154 2.988 2.098 3.346 2.753 16.699 1.292 1.828 2.829 2.878 4.023

^{1}H NMR of 6f

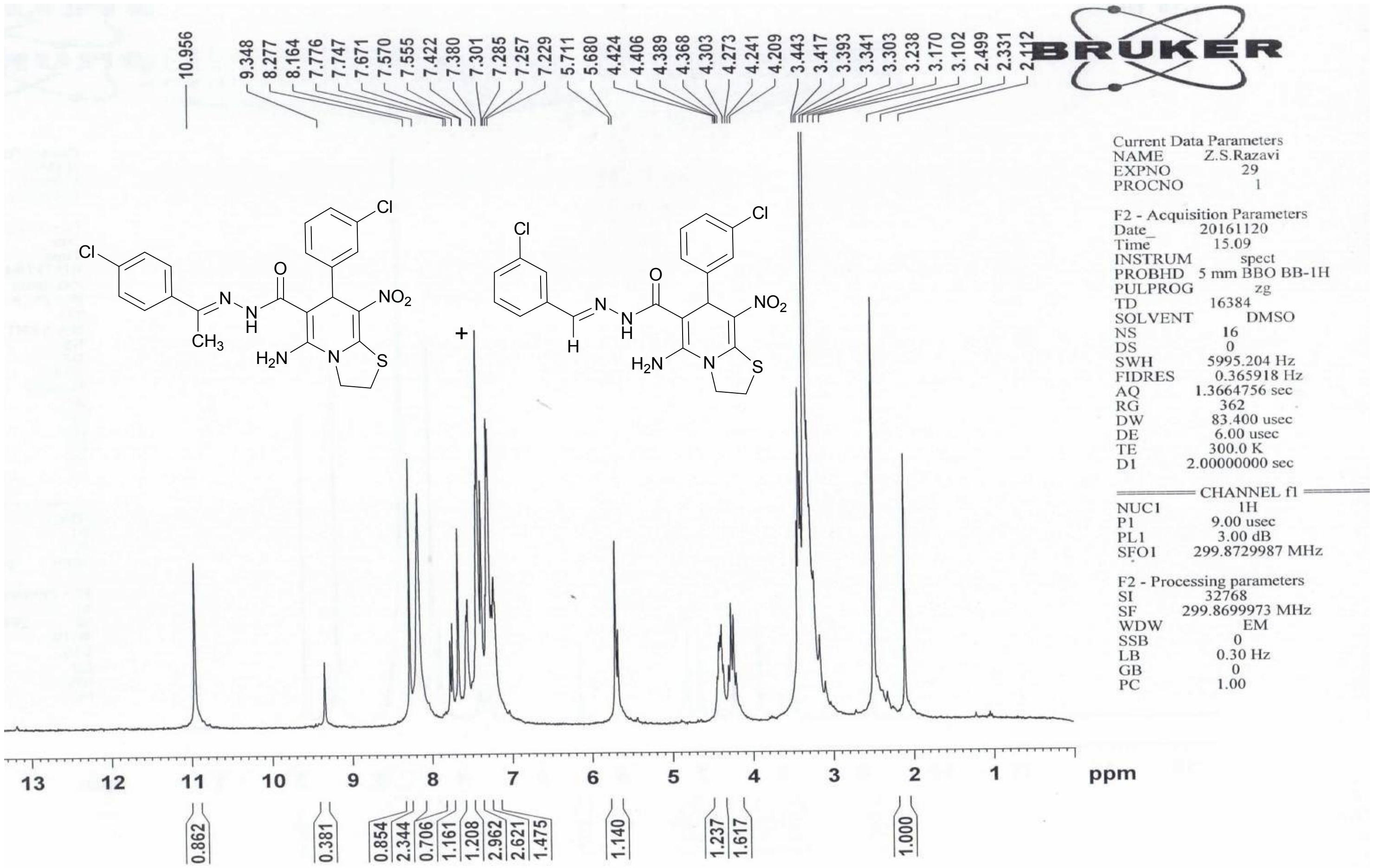
10.956
9.348
8.277
8.164
7.776
7.747
7.671
7.570
7.555
7.422
7.380
7.301
7.285
7.257
7.229
5.711
5.680
4.424
4.406
4.389
4.368
4.303
4.273
4.241
4.209
3.443
3.417
3.393
3.341
3.303
3.238
3.170
3.102
2.499
2.331
2.112
BRUKER
Cl
Cl
O
N
N
H
NO2
CH3
H2N
N
S
+
Cl
Cl
O
N
N
H
NO2
H
H2N
N
S
Current Data Parameters
NAME Z.S.Razavi
EXPNO 29
PROCNO 1
F2 - Acquisition Parameters
Date_ 20161120
Time 15.09
INSTRUM spect
PROBHD 5 mm BBO BB-1H
PULPROG zg
TD 16384
SOLVENT DMSO
NS 16
DS 0
SWH 5995.204 Hz
FIDRES 0.365918 Hz
AQ 1.3664756 sec
RG 362
DW 83.400 usec
DE 6.00 usec
TE 300.0 K
D1 2.00000000 sec
CHANNEL f1
NUC1 1H
P1 9.00 usec
PL1 3.00 dB
SFO1 299.8729987 MHz
F2 - Processing parameters
SI 32768
SF 299.8699973 MHz
WDW EM
SSB 0
LB 0.30 Hz
GB 0
PC 1.00
13 12 11 10 9 8 7 6 5 4 3 2 1 ppm
0.862
0.381
0.854
2.344
0.706
1.161
1.208
2.962
2.621
1.475
1.140
1.237
1.617
1.000

^{1}H NMR of 6g

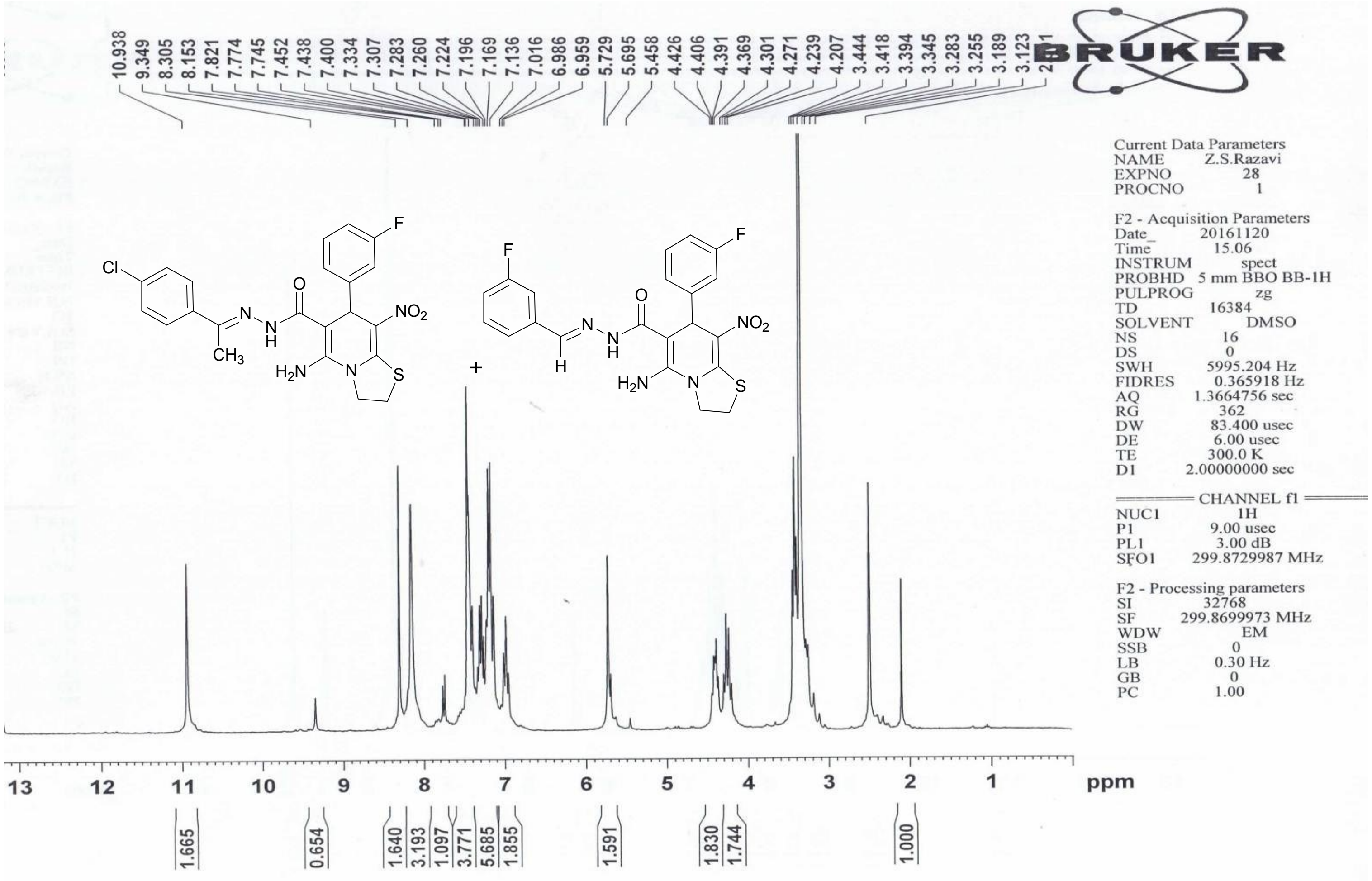

10.938
9.349
8.305
8.153
7.821
7.774
7.745
7.452
7.438
7.400
7.334
7.307
7.283
7.260
7.224
7.196
7.169
7.136
7.016
6.986
6.959
5.729
5.695
5.458
4.426
4.406
4.391
4.369
4.301
4.271
4.239
4.207
3.444
3.418
3.394
3.345
3.283
3.255
3.189
3.123
BRUKER
Cl
F
O
N
H
NO2
CH3
H2N
S
+
Current Data Parameters
NAME Z.S.Razavi
EXPNO 28
PROCNO 1
F2 - Acquisition Parameters
Date_ 20161120
Time 15.06
INSTRUM spect
PROBHD 5 mm BBO BB-1H
PULPROG zg
TD 16384
SOLVENT DMSO
NS 16
DS 0
SWH 5995.204 Hz
FIDRES 0.365918 Hz
AQ 1.3664756 sec
RG 362
DW 83.400 usec
DE 6.00 usec
TE 300.0 K
D1 2.00000000 sec
CHANNEL f1
NUC1 1H
P1 9.00 usec
PL1 3.00 dB
SFO1 299.8729987 MHz
F2 - Processing parameters
SI 32768
SF 299.8699973 MHz
WDW EM
SSB 0
LB 0.30 Hz
GB 0
PC 1.00
13 12 11 10 9 8 7 6 5 4 3 2 1 ppm
1.665
0.654
1.640
3.193
1.097
3.771
5.685
1.855
1.591
1.830
1.744
1.000

^{1}H NMR of 6h

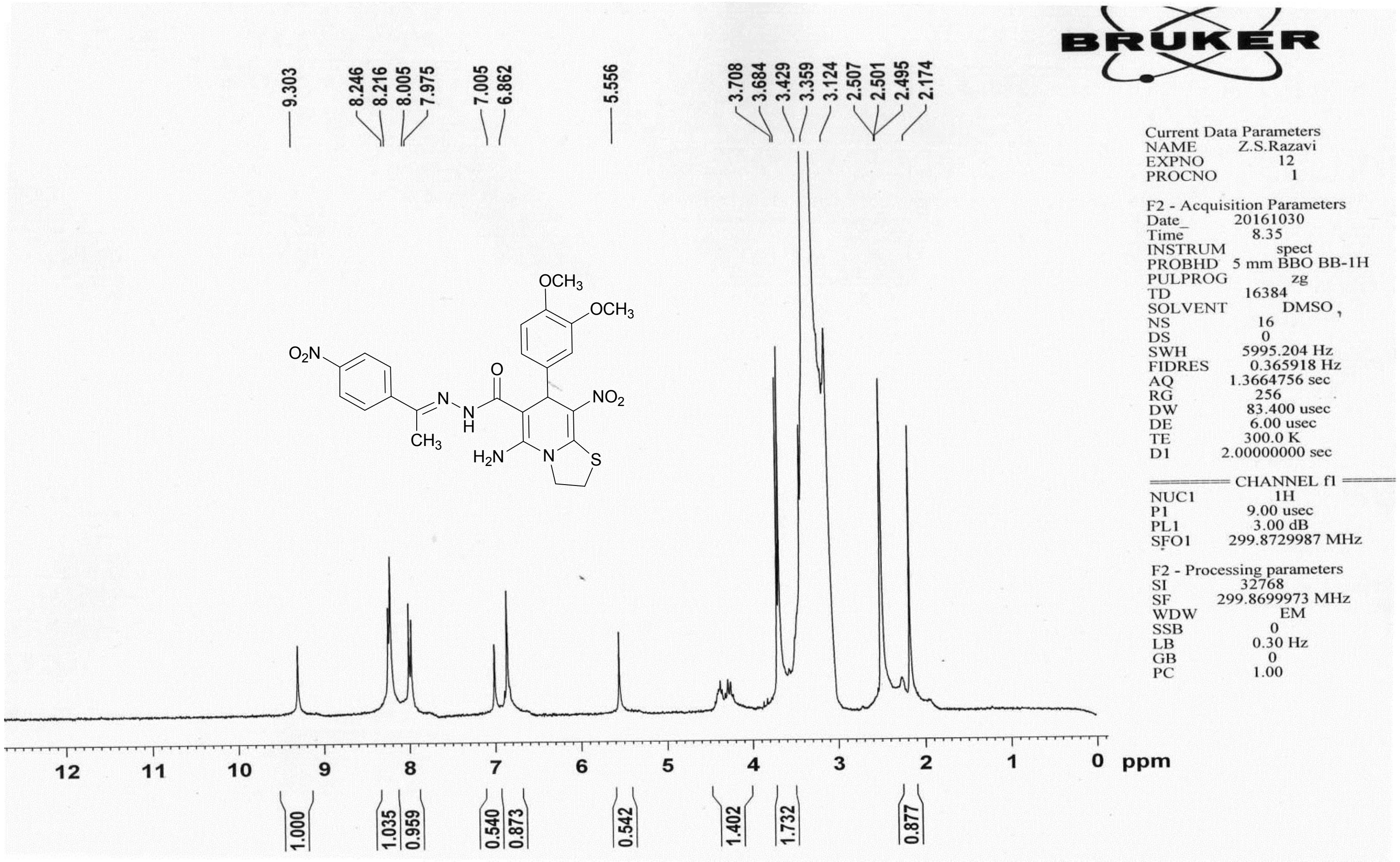
9.303
8.246
8.216
8.005
7.975
7.005
6.862
5.556
3.708
3.684
3.429
3.359
3.124
2.507
2.501
2.495
2.174
Current Data Parameters
NAME Z.S.Razavi
EXPNO 12
PROCNO 1
F2 - Acquisition Parameters
Date_ 20161030
Time 8.35
INSTRUM spect
PROBHD 5 mm BBO BB-1H
PULPROG zg
TD 16384
SOLVENT DMSO
NS 16
DS 0
SWH 5995.204 Hz
FIDRES 0.365918 Hz
AQ 1.3664756 sec
RG 256
DW 83.400 usec
DE 6.00 usec
TE 300.0 K
D1 2.00000000 sec
CHANNEL f1
NUC1 1H
P1 9.00 usec
PL1 3.00 dB
SFO1 299.8729987 MHz
F2 - Processing parameters
SI 32768
SF 299.8699973 MHz
WDW EM
SSB 0
LB 0.30 Hz
GB 0
PC 1.00
ppm
1.000
1.035
0.959
0.540
0.873
0.542
1.402
1.732
0.877

^{1}H NMR of 6i

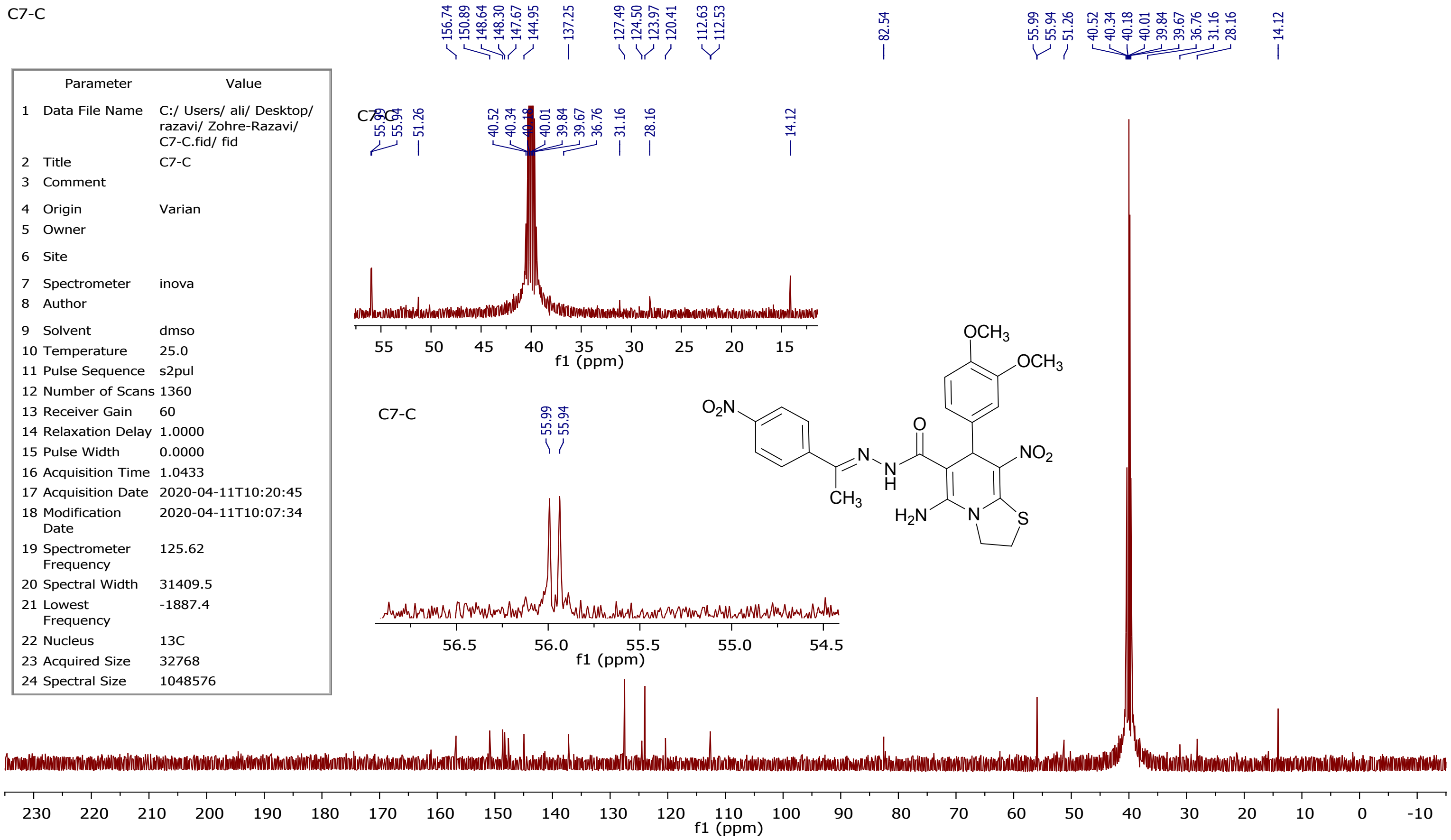
C7-C
Parameter Value
1 Data File Name C:/ Users/ ali/ Desktop/ razavi/ Zohre-Razavi/ C7-C.fid/ fid
2 Title C7-C
3 Comment
4 Origin Varian
5 Owner
6 Site
7 Spectrometer inova
8 Author
9 Solvent dmso
10 Temperature 25.0
11 Pulse Sequence s2pul
12 Number of Scans 1360
13 Receiver Gain 60
14 Relaxation Delay 1.0000
15 Pulse Width 0.0000
16 Acquisition Time 1.0433
17 Acquisition Date 2020-04-11T10:20:45
18 Modification Date 2020-04-11T10:07:34
19 Spectrometer Frequency 125.62
20 Spectral Width 31409.5
21 Lowest Frequency -1887.4
22 Nucleus 13C
23 Acquired Size 32768
24 Spectral Size 1048576
156.74 150.89 148.64 148.30 147.67 144.95 137.25 127.49 124.50 123.97 120.41 112.63 112.53 82.54 55.99 55.94 51.26 40.52 40.34 40.18 40.01 39.84 39.67 36.76 31.16 28.16 14.12
f1 (ppm)
C7-C
55.99 55.94
f1 (ppm)
OCH3
OCH3
O2N
O
N
N
H
NO2
CH3
H2N
N
S

^{13}**C NMR of 6i**

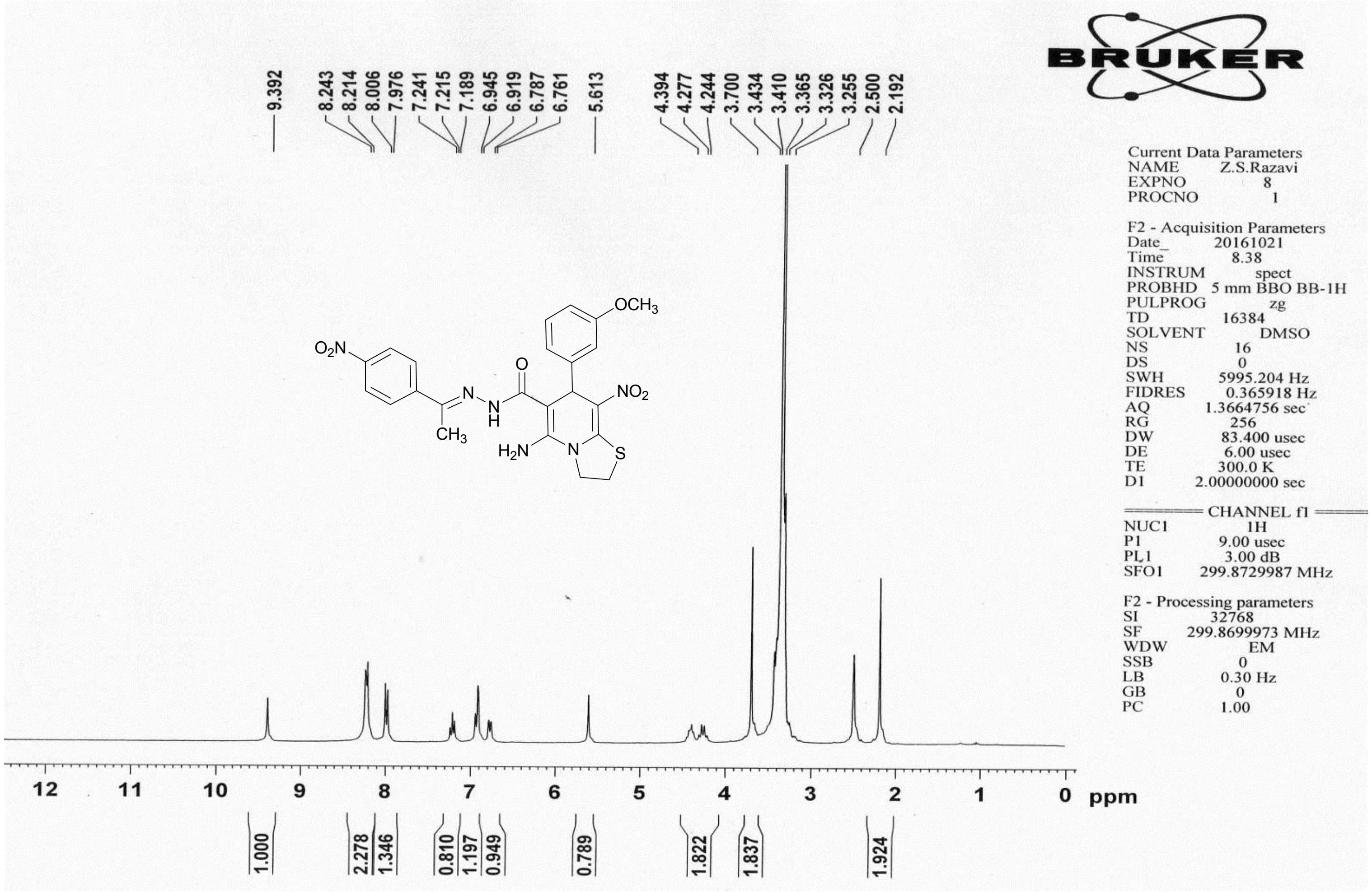
9.392
8.243
8.214
8.006
7.976
7.241
7.215
7.189
6.945
6.919
6.787
6.761
5.613
4.394
4.277
4.244
3.700
3.434
3.410
3.365
3.326
3.255
2.500
2.192
BRUKER
OCH3
O2N
O
NO2
N
N
H
CH3
H2N
N
S
Current Data Parameters
NAME Z.S.Razavi
EXPNO 8
PROCNO 1
F2 - Acquisition Parameters
Date_ 20161021
Time 8.38
INSTRUM spect
PROBHD 5 mm BBO BB-1H
PULPROG zg
TD 16384
SOLVENT DMSO
NS 16
DS 0
SWH 5995.204 Hz
FIDRES 0.365918 Hz
AQ 1.3664756 sec
RG 256
DW 83.400 usec
DE 6.00 usec
TE 300.0 K
D1 2.00000000 sec
CHANNEL f1
NUC1 1H
P1 9.00 usec
PL1 3.00 dB
SFO1 299.8729987 MHz
F2 - Processing parameters
SI 32768
SF 299.8699973 MHz
WDW EM
SSB 0
LB 0.30 Hz
GB 0
PC 1.00
12 11 10 9 8 7 6 5 4 3 2 1 0 ppm
1.000
2.278
1.346
0.810
1.197
0.949
0.789
1.822
1.837
1.924

^{1}H NMR of 6j

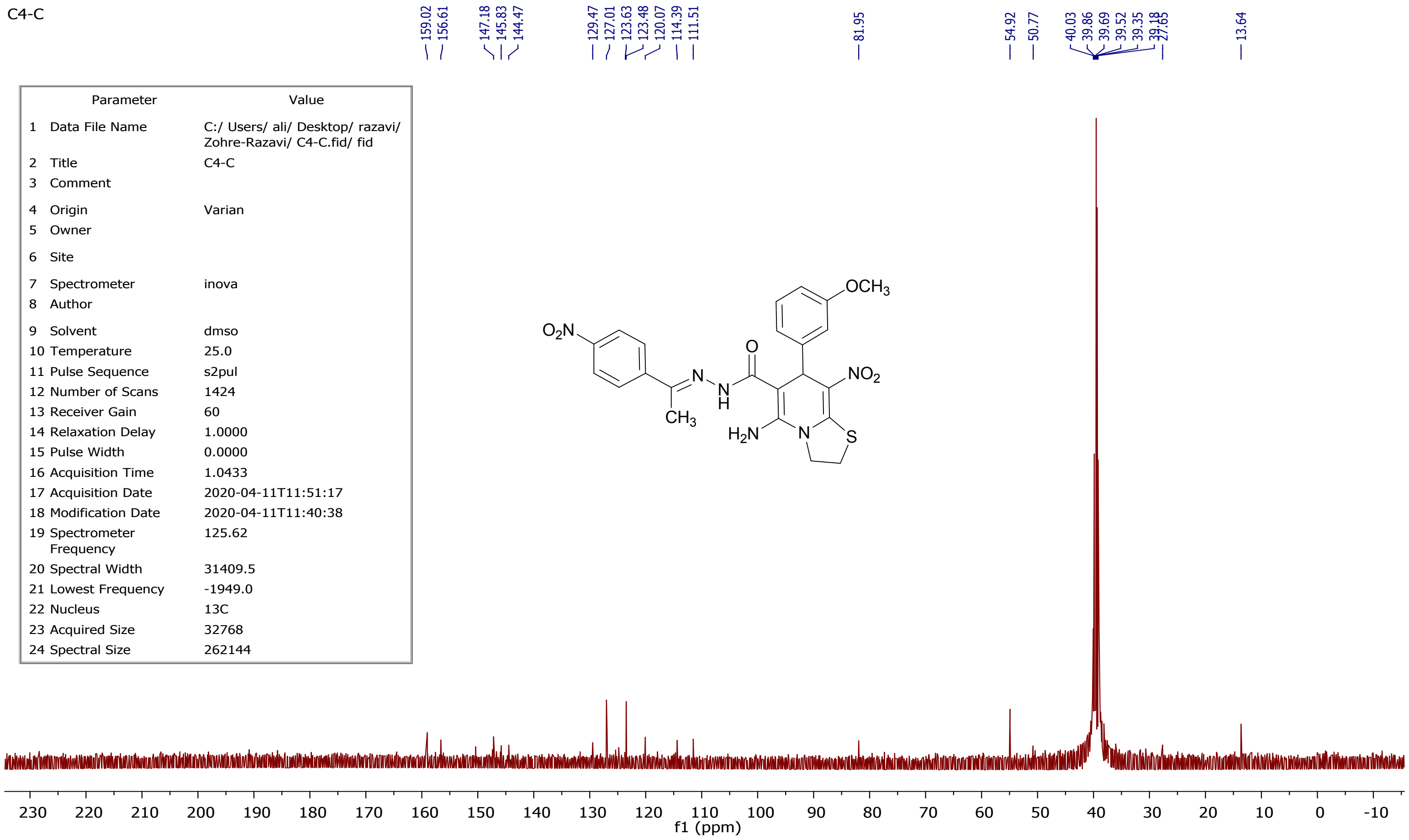

	Parameter	Value
1	Data File Name	C:/ Users/ ali/ Desktop/ razavi/ Zohre-Razavi/ C4-C.fid/ fid
2	Title	C4-C
3	Comment	
4	Origin	Varian
5	Owner	
6	Site	
7	Spectrometer	inova
8	Author	
9	Solvent	dmso
10	Temperature	25.0
11	Pulse Sequence	s2pul
12	Number of Scans	1424
13	Receiver Gain	60
14	Relaxation Delay	1.0000
15	Pulse Width	0.0000
16	Acquisition Time	1.0433
17	Acquisition Date	2020-04-11T11:51:17
18	Modification Date	2020-04-11T11:40:38
19	Spectrometer Frequency	125.62
20	Spectral Width	31409.5
21	Lowest Frequency	-1949.0
22	Nucleus	13C
23	Acquired Size	32768
24	Spectral Size	262144

^{13}C NMR of 6j

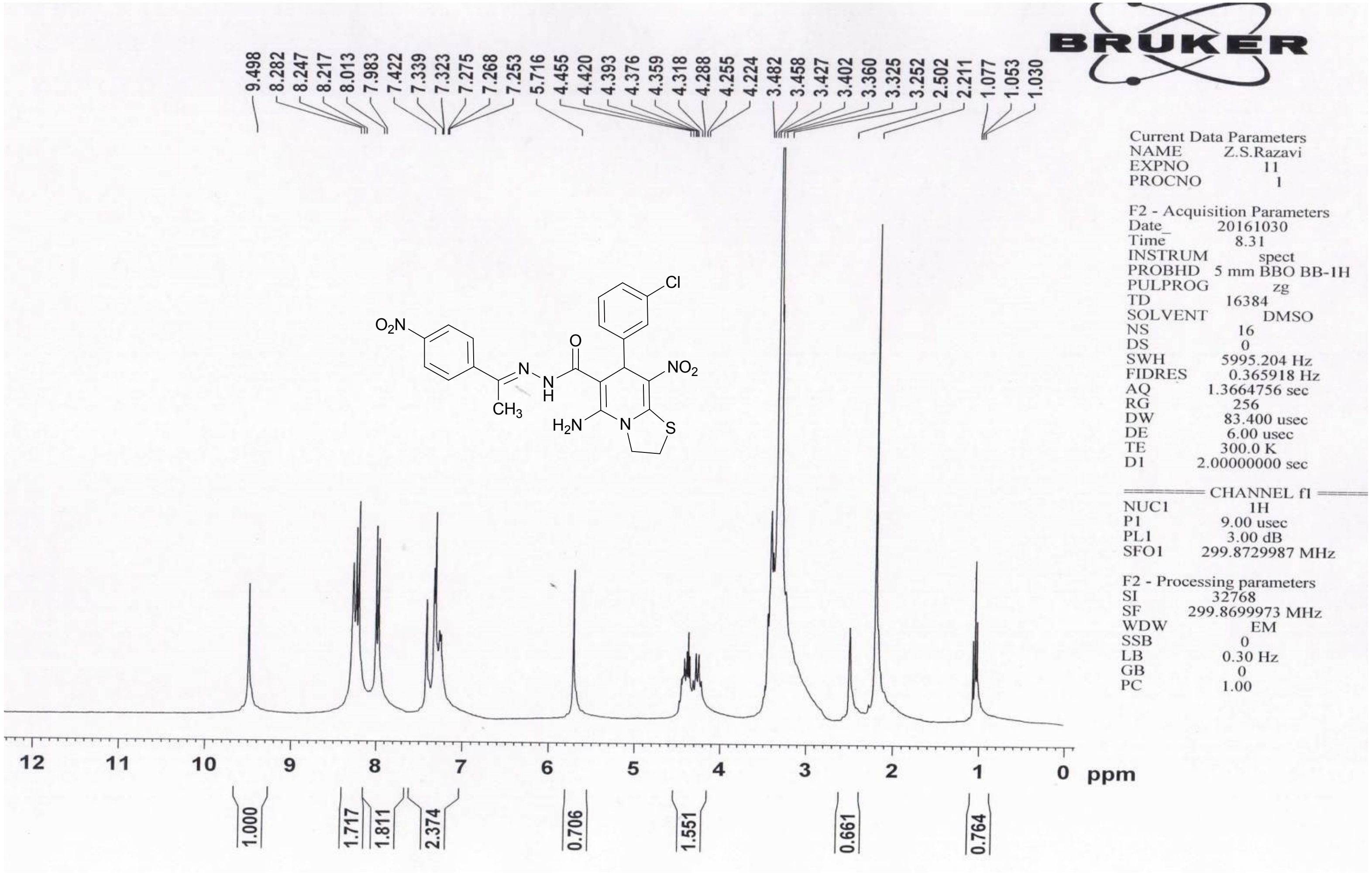
9.498
8.282
8.247
8.217
8.013
7.983
7.422
7.339
7.323
7.275
7.268
7.253
5.716
4.455
4.420
4.393
4.376
4.359
4.318
4.288
4.255
4.224
3.482
3.458
3.427
3.402
3.360
3.325
3.252
2.502
2.211
1.077
1.053
1.030
BRUKER
Current Data Parameters
NAME Z.S.Razavi
EXPNO 11
PROCNO 1
F2 - Acquisition Parameters
Date_ 20161030
Time 8.31
INSTRUM spect
PROBHD 5 mm BBO BB-1H
PULPROG zg
TD 16384
SOLVENT DMSO
NS 16
DS 0
SWH 5995.204 Hz
FIDRES 0.365918 Hz
AQ 1.3664756 sec
RG 256
DW 83.400 usec
DE 6.00 usec
TE 300.0 K
D1 2.00000000 sec
CHANNEL f1
NUC1 1H
P1 9.00 usec
PL1 3.00 dB
SFO1 299.8729987 MHz
F2 - Processing parameters
SI 32768
SF 299.8699973 MHz
WDW EM
SSB 0
LB 0.30 Hz
GB 0
PC 1.00
Cl
O
O2N
N
NO2
N
H
CH3
H2N
N
S
12
11
10
9
8
7
6
5
4
3
2
1
0
ppm
1.000
1.717
1.811
2.374
0.706
1.551
0.661
0.764

^{1}H NMR of 6k

C6-C

	Parameter	Value
1	Data File Name	C:/ Users/ ali/ Desktop/ NMR MN/ Masoud-Mohammadivala/ C6-C.fid/ fid
2	Title	C6-C
3	Comment	
4	Origin	Varian
5	Owner	
6	Site	
7	Spectrometer	inova
8	Author	
9	Solvent	dmso
10	Temperature	25.0
11	Pulse Sequence	s2pul
12	Number of Scans	2048
13	Receiver Gain	60
14	Relaxation Delay	1.0000
15	Pulse Width	0.0000
16	Acquisition Time	1.0433
17	Acquisition Date	2020-07-20T15:27:39
18	Modification Date	2020-07-20T14:32:24
19	Spectrometer Frequency	125.62
20	Spectral Width	31409.5
21	Lowest Frequency	-1945.6
22	Nucleus	13C
23	Acquired Size	32768
24	Spectral Size	524288

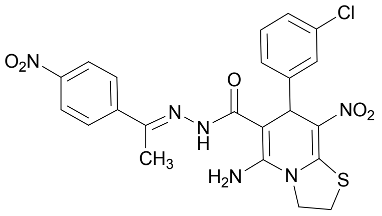

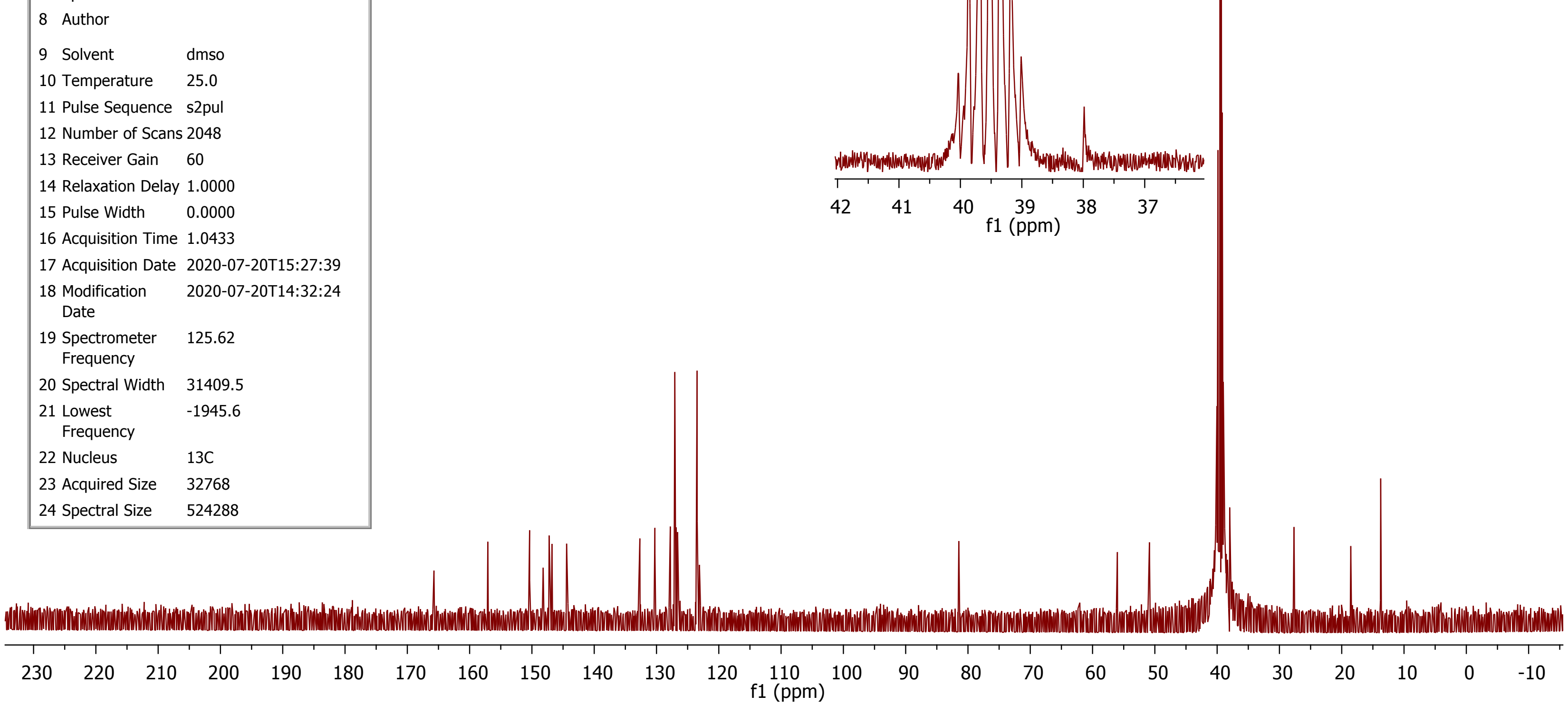

^{13}C NMR of 6k

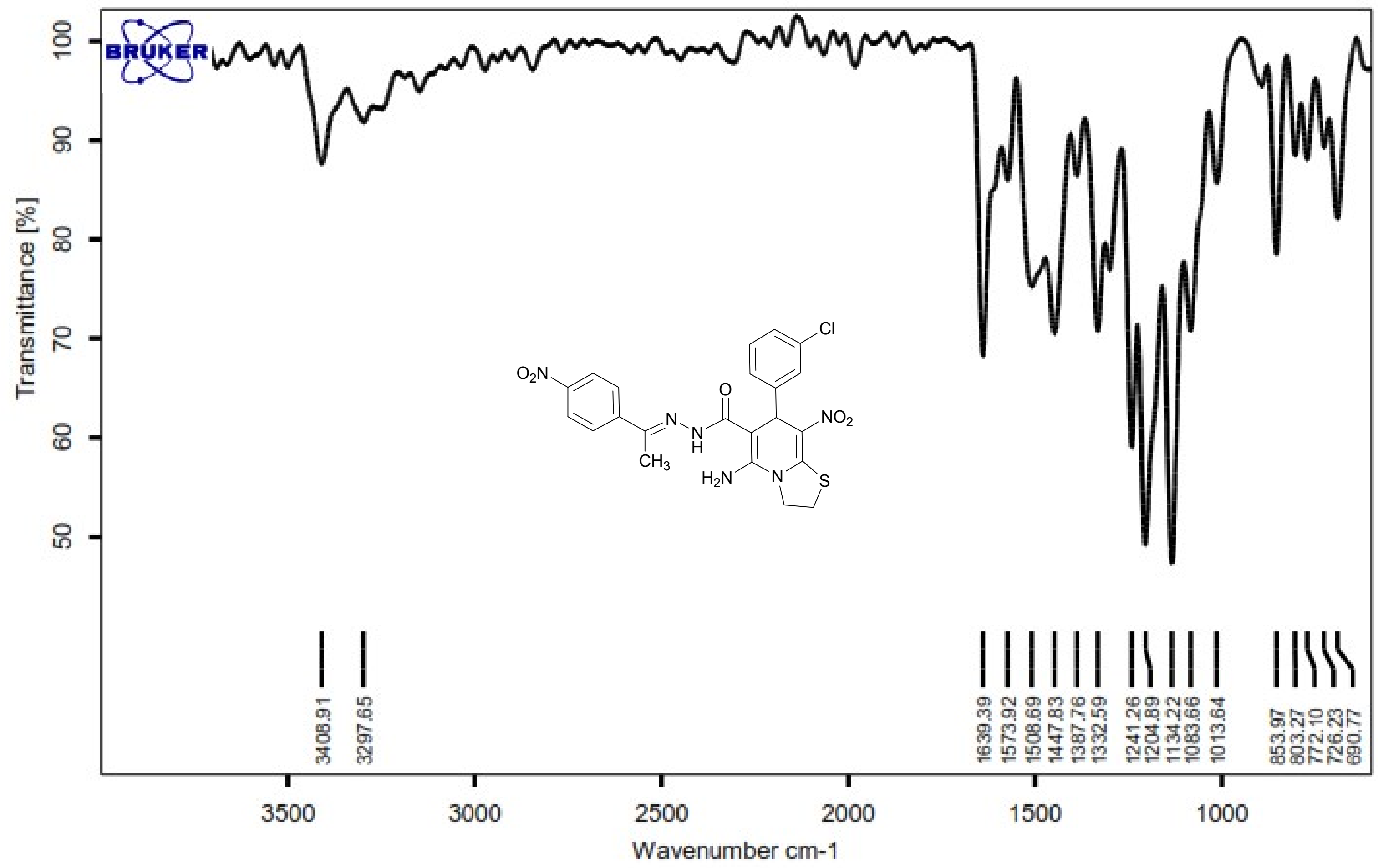

BRUKER
Transmittance [%]
100
90
80
70
60
50
Wavenumber cm-1
3500
3000
2500
2000
1500
1000
3408.91
3297.65
1639.39
1573.92
1508.69
1447.83
1387.76
1332.59
1241.26
1204.89
1134.22
1083.66
1013.64
853.97
803.27
772.10
726.23
690.77
O2N
O
NO2
Cl
N
N
H
CH3
H2N
N
S

IR of 6k

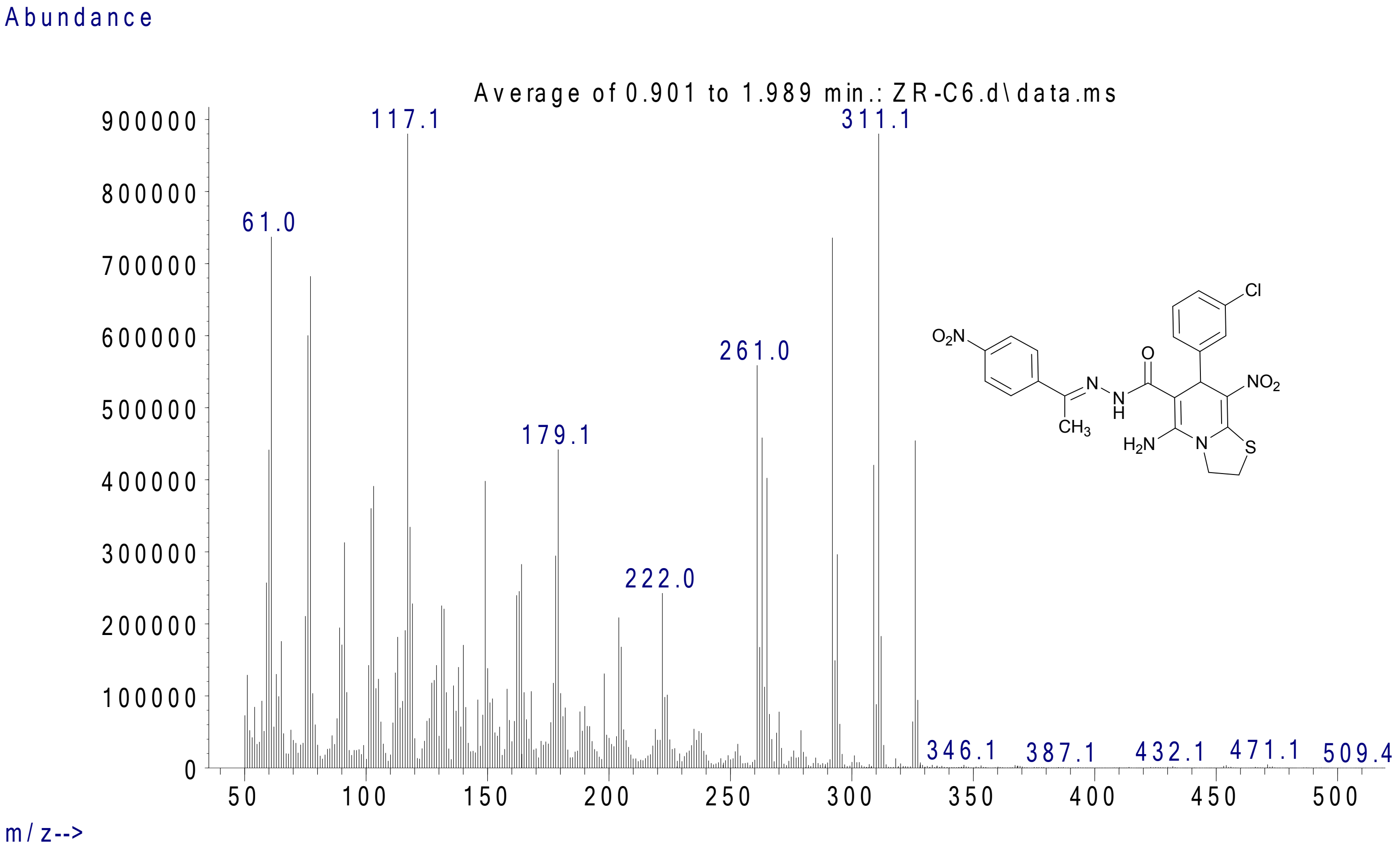
Abundance
Average of 0.901 to 1.989 min.: ZR-C6.d\data.ms
900000
800000
700000
600000
500000
400000
300000
200000
100000
0
117.1
311.1
61.0
261.0
179.1
222.0
346.1
387.1
432.1
471.1
509.4
50
100
150
200
250
300
350
400
450
500
m/z-->
Cl
O2N
O
NO2
N
N
H
CH3
H2N
N
S

MS of 6k

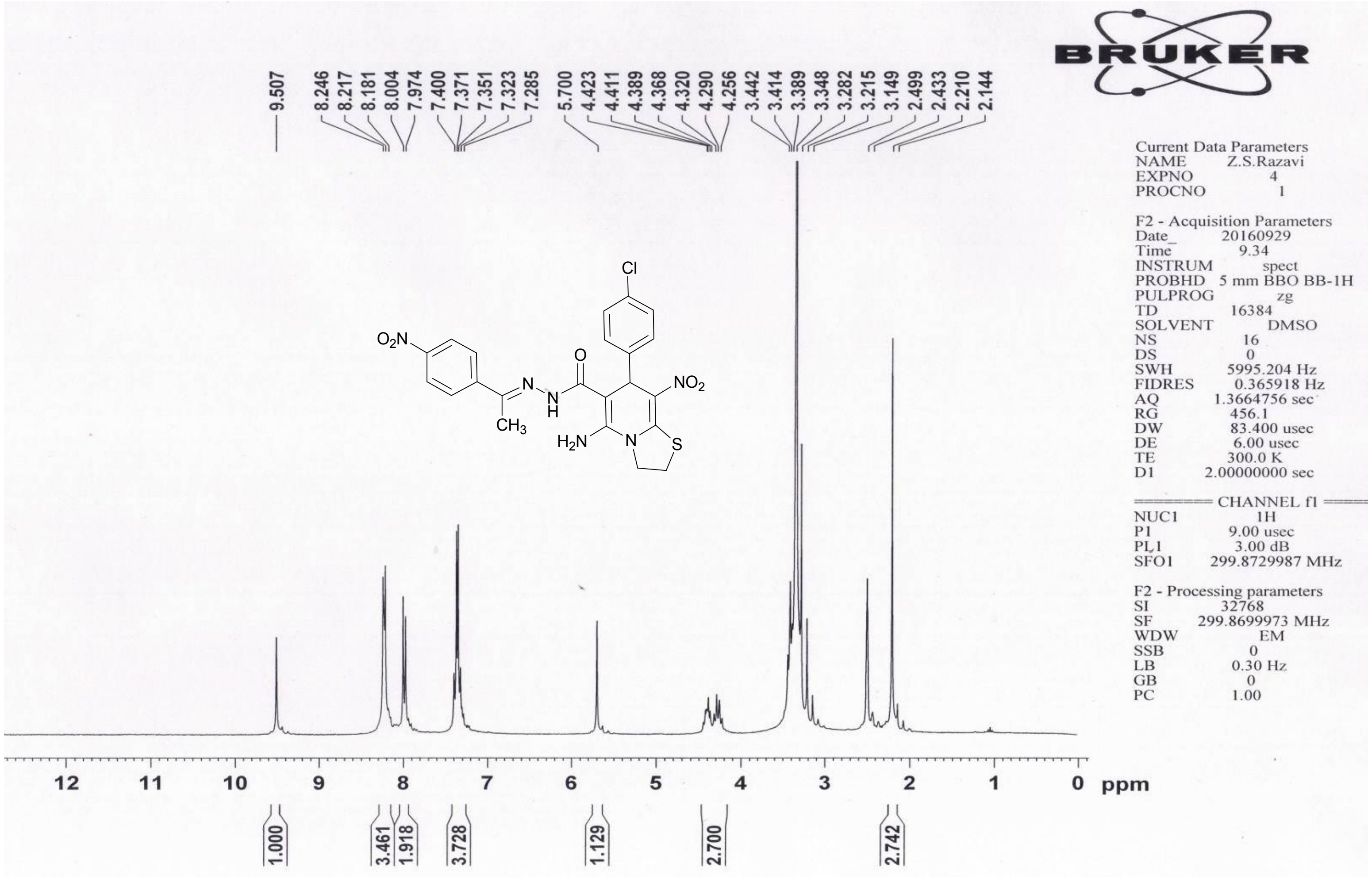
9.507
8.246
8.217
8.181
8.004
7.974
7.400
7.371
7.351
7.323
7.285
5.700
4.423
4.411
4.389
4.368
4.320
4.290
4.256
3.442
3.414
3.389
3.348
3.282
3.215
3.149
2.499
2.433
2.210
2.144
BRUKER
Current Data Parameters
NAME Z.S.Razavi
EXPNO 4
PROCNO 1
F2 - Acquisition Parameters
Date_ 20160929
Time 9.34
INSTRUM spect
PROBHD 5 mm BBO BB-1H
PULPROG zg
TD 16384
SOLVENT DMSO
NS 16
DS 0
SWH 5995.204 Hz
FIDRES 0.365918 Hz
AQ 1.3664756 sec
RG 456.1
DW 83.400 usec
DE 6.00 usec
TE 300.0 K
D1 2.00000000 sec
CHANNEL f1
NUC1 1H
P1 9.00 usec
PL1 3.00 dB
SFO1 299.8729987 MHz
F2 - Processing parameters
SI 32768
SF 299.8699973 MHz
WDW EM
SSB 0
LB 0.30 Hz
GB 0
PC 1.00
Cl
O2N
O
NO2
N
N
H
CH3
H2N
N
S
12 11 10 9 8 7 6 5 4 3 2 1 0 ppm
1.000
3.461
1.918
3.728
1.129
2.700
2.742

^{1}H NMR of 6l

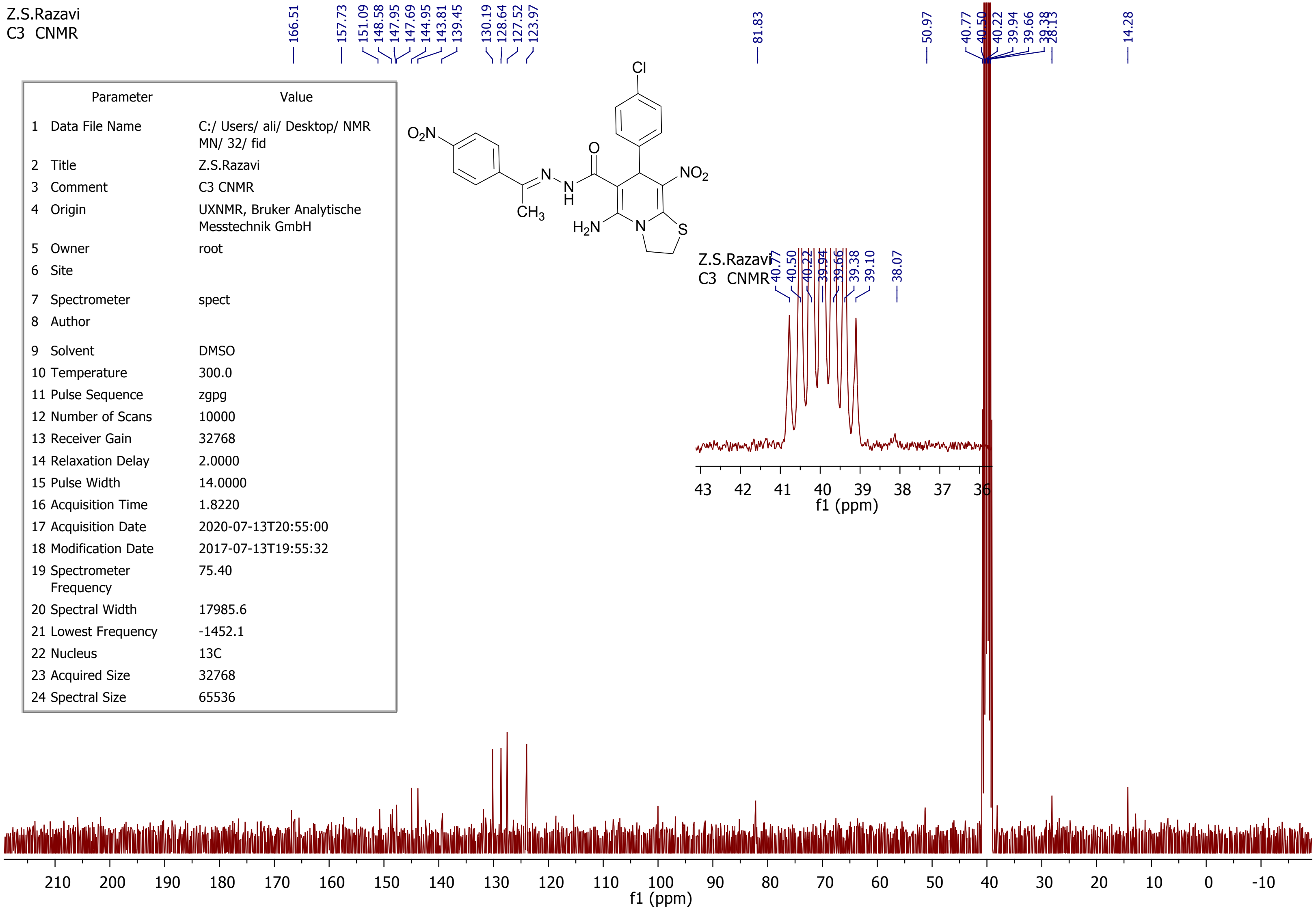

	Parameter	Value
1	Data File Name	C:/ Users/ ali/ Desktop/ NMR MN/ 32/ fid
2	Title	Z.S.Razavi
3	Comment	C3 CNMR
4	Origin	UXNMR, Bruker Analytische Messtechnik GmbH
5	Owner	root
6	Site	
7	Spectrometer	spect
8	Author	
9	Solvent	DMSO
10	Temperature	300.0
11	Pulse Sequence	zgpg
12	Number of Scans	10000
13	Receiver Gain	32768
14	Relaxation Delay	2.0000
15	Pulse Width	14.0000
16	Acquisition Time	1.8220
17	Acquisition Date	2020-07-13T20:55:00
18	Modification Date	2017-07-13T19:55:32
19	Spectrometer Frequency	75.40
20	Spectral Width	17985.6
21	Lowest Frequency	-1452.1
22	Nucleus	13C
23	Acquired Size	32768
24	Spectral Size	65536

^{13}C NMR of 6l

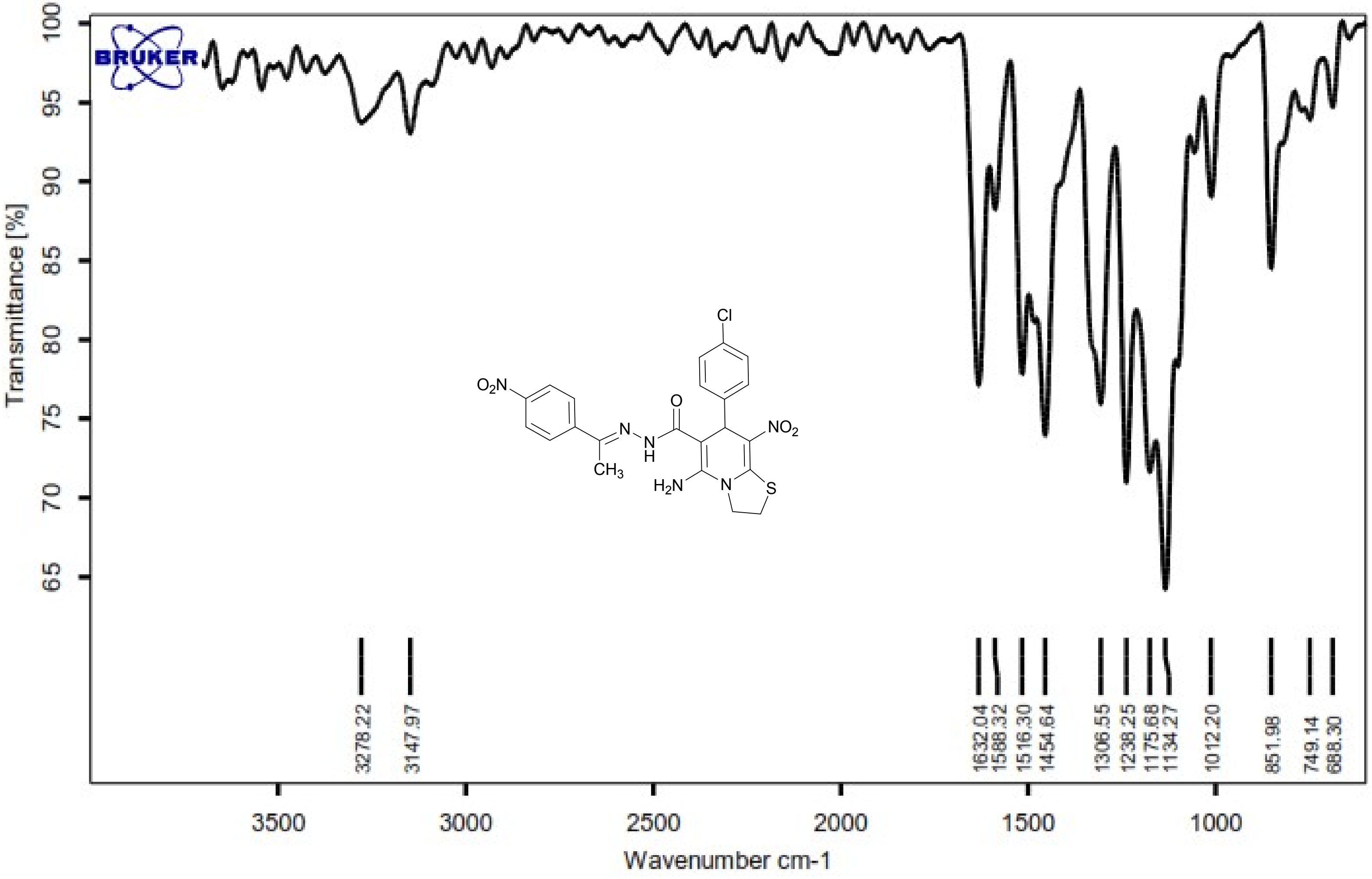
BRUKER
Transmittance [%]
100
95
90
85
80
75
70
65
Wavenumber cm-1
3500
3000
2500
2000
1500
1000
3278.22
3147.97
1632.04
1588.32
1516.30
1454.64
1306.55
1238.25
1175.68
1134.27
1012.20
851.98
749.14
688.30
Cl
O_2N
NO_2
O
N
N
H
CH_3
H_2N
N
S

IR of 6l

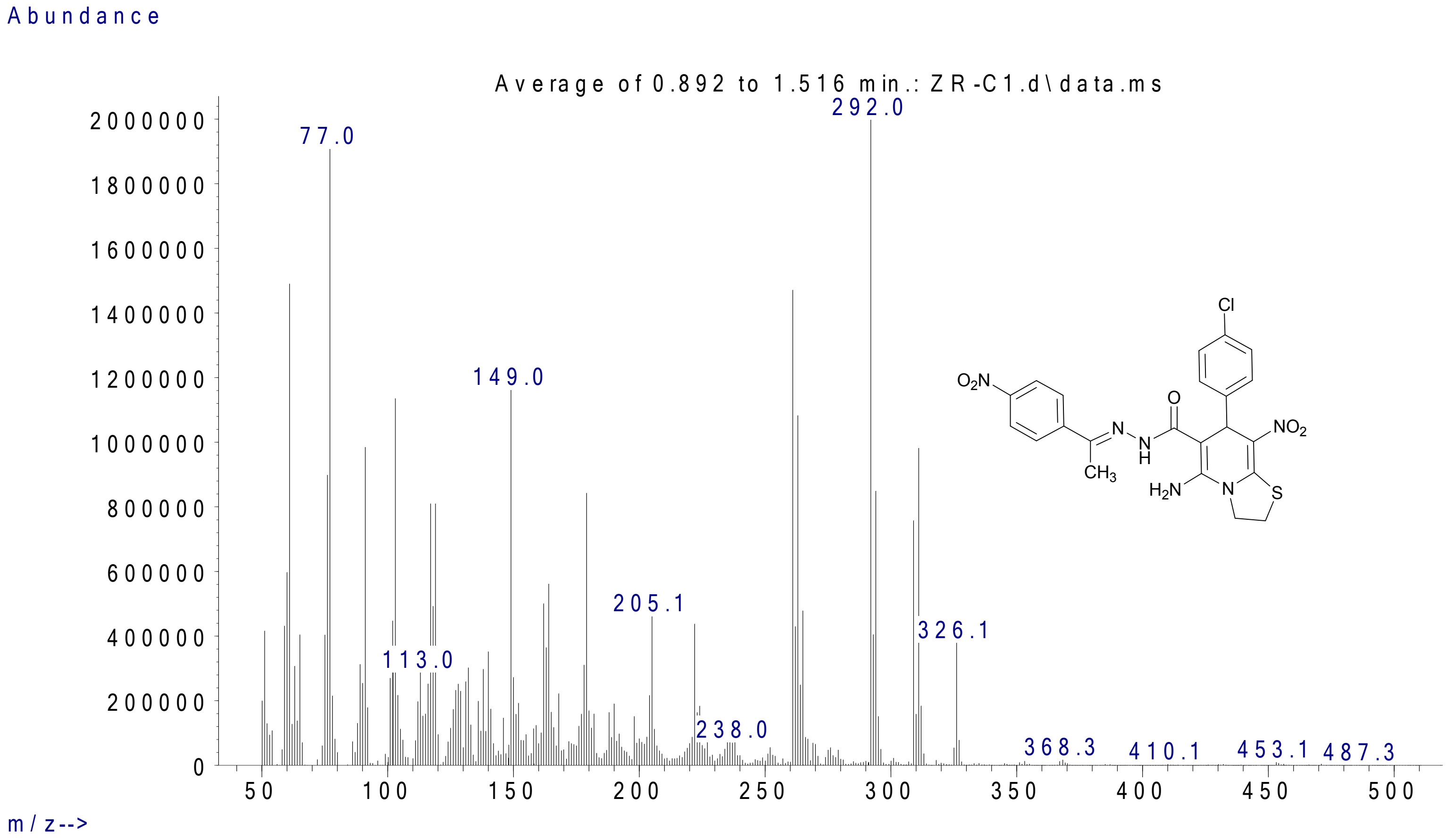
Abundance
Average of 0.892 to 1.516 min.: ZR-C1.d\data.ms
2000000
1800000
1600000
1400000
1200000
1000000
800000
600000
400000
200000
0
77.0
292.0
149.0
113.0
205.1
238.0
326.1
368.3
410.1
453.1
487.3
50
100
150
200
250
300
350
400
450
500
m/z-->
Cl
O2N
O
N
N
H
NO2
CH3
H2N
N
S

MS of 6l

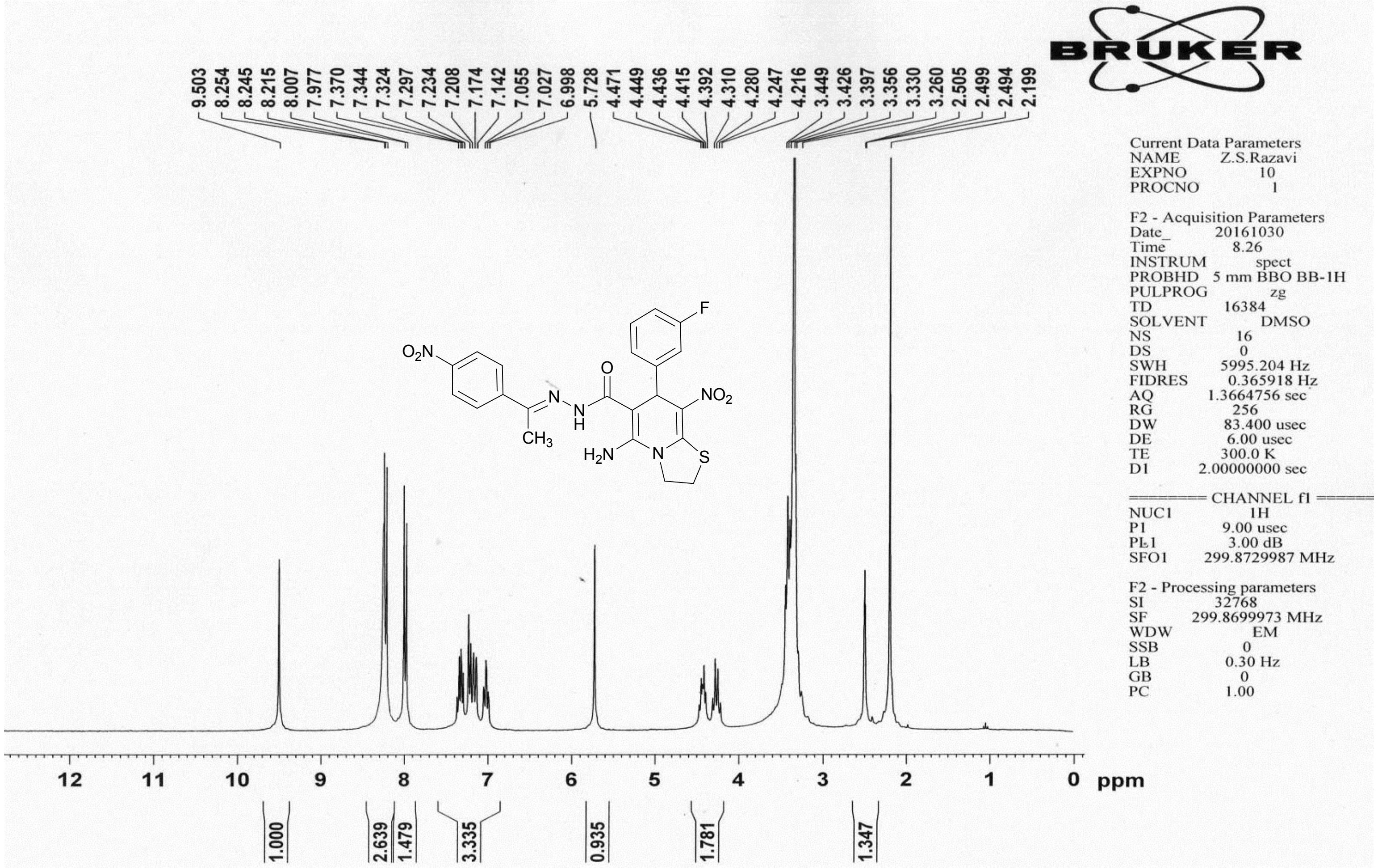

^{1}H NMR of 6m

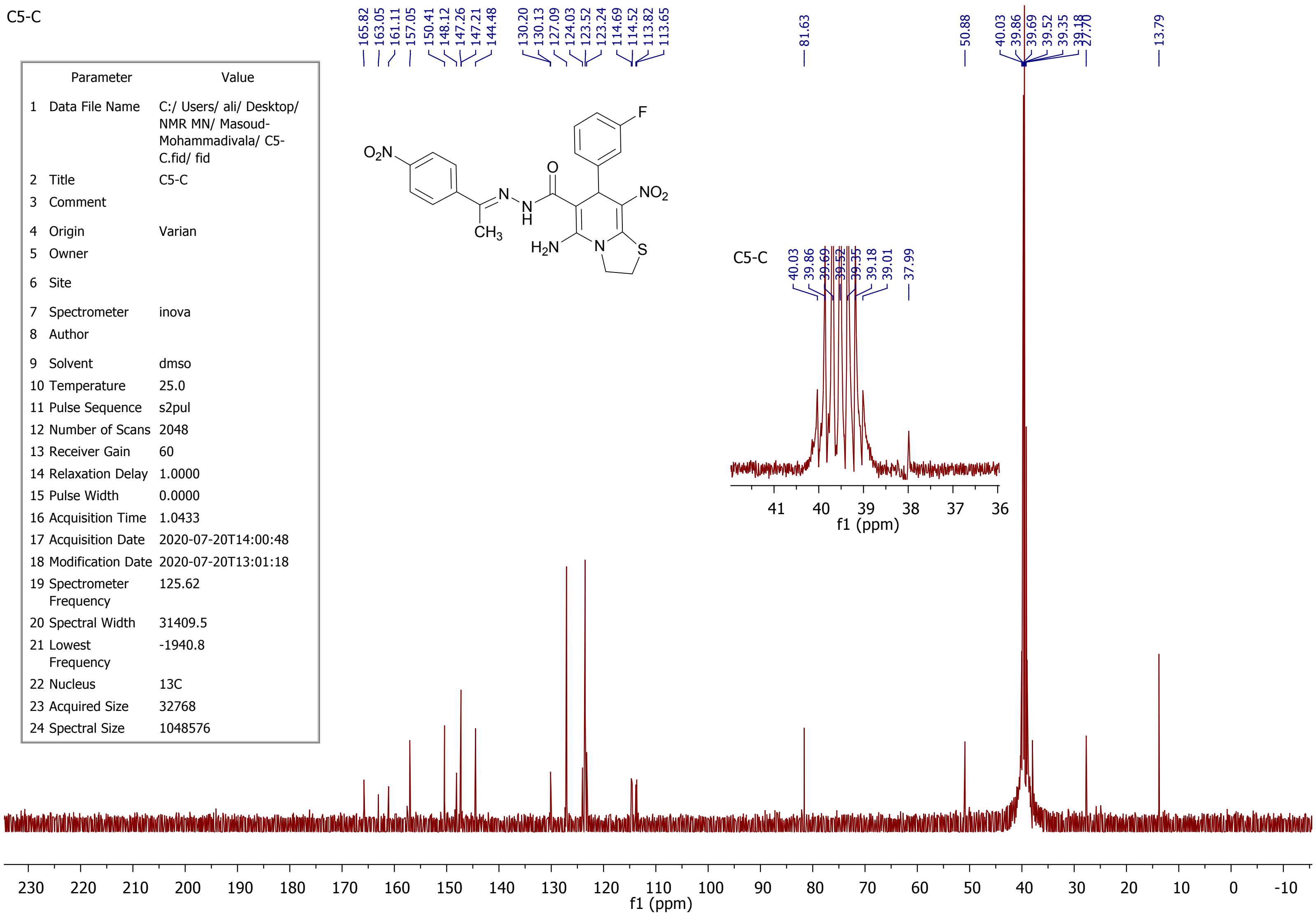

	Parameter	Value
1	Data File Name	C:/ Users/ ali/ Desktop/ NMR MN/ Masoud-Mohammadivala/ C5-C.fid/ fid
2	Title	C5-C
3	Comment	
4	Origin	Varian
5	Owner	
6	Site	
7	Spectrometer	inova
8	Author	
9	Solvent	dmso
10	Temperature	25.0
11	Pulse Sequence	s2pul
12	Number of Scans	2048
13	Receiver Gain	60
14	Relaxation Delay	1.0000
15	Pulse Width	0.0000
16	Acquisition Time	1.0433
17	Acquisition Date	2020-07-20T14:00:48
18	Modification Date	2020-07-20T13:01:18
19	Spectrometer Frequency	125.62
20	Spectral Width	31409.5
21	Lowest Frequency	-1940.8
22	Nucleus	13C
23	Acquired Size	32768
24	Spectral Size	1048576

^{13}C NMR of 6m

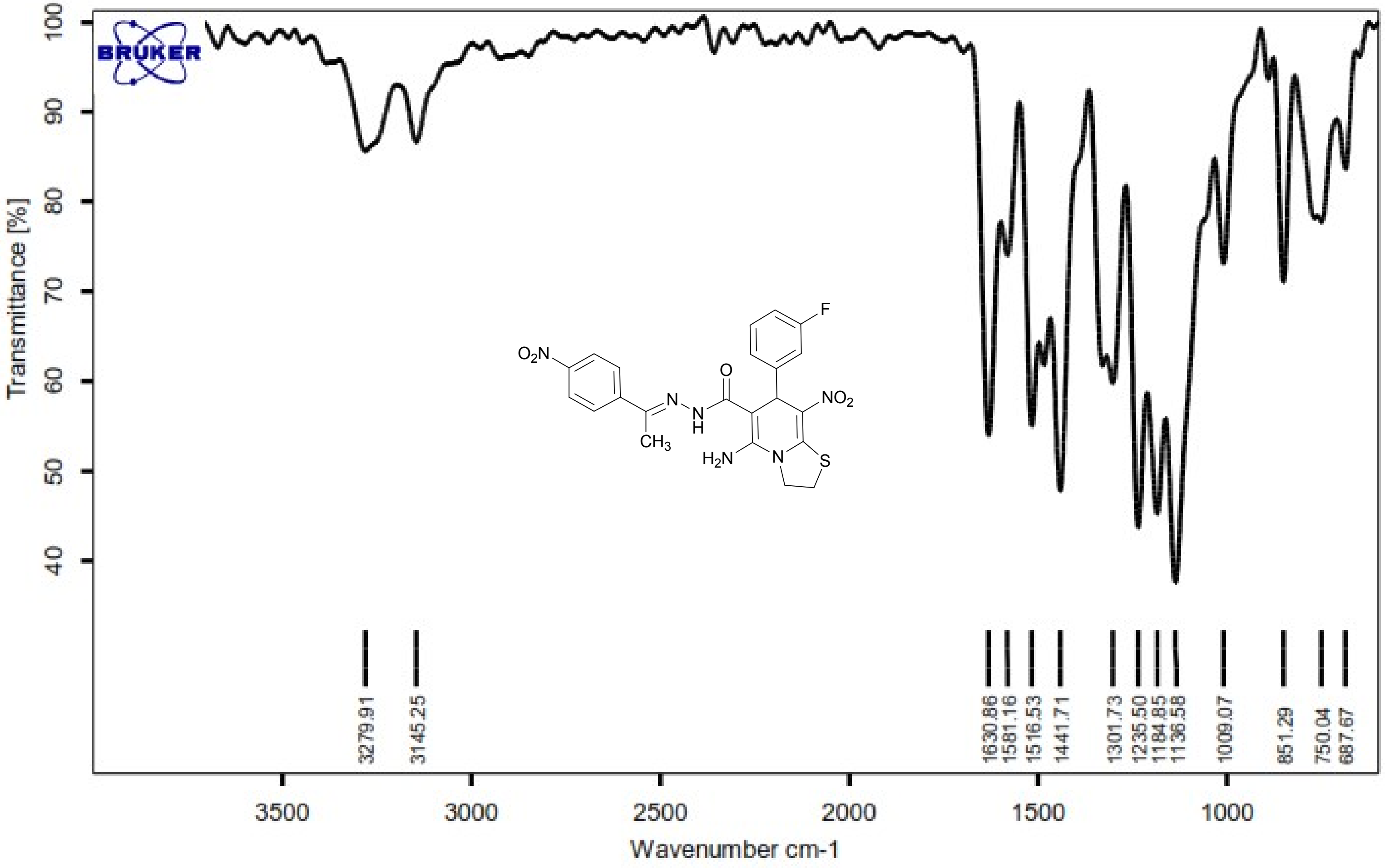
BRUKER
Transmittance [%]
100
90
80
70
60
50
40
3279.91
3145.25
1630.86
1581.16
1516.53
1441.71
1301.73
1235.50
1184.85
1136.58
1009.07
851.29
750.04
687.67
3500
3000
2500
2000
1500
1000
Wavenumber cm-1
F
O_2N
O
NO_2
N
N
H
CH_3
H_2N
N
S

IR of 6m

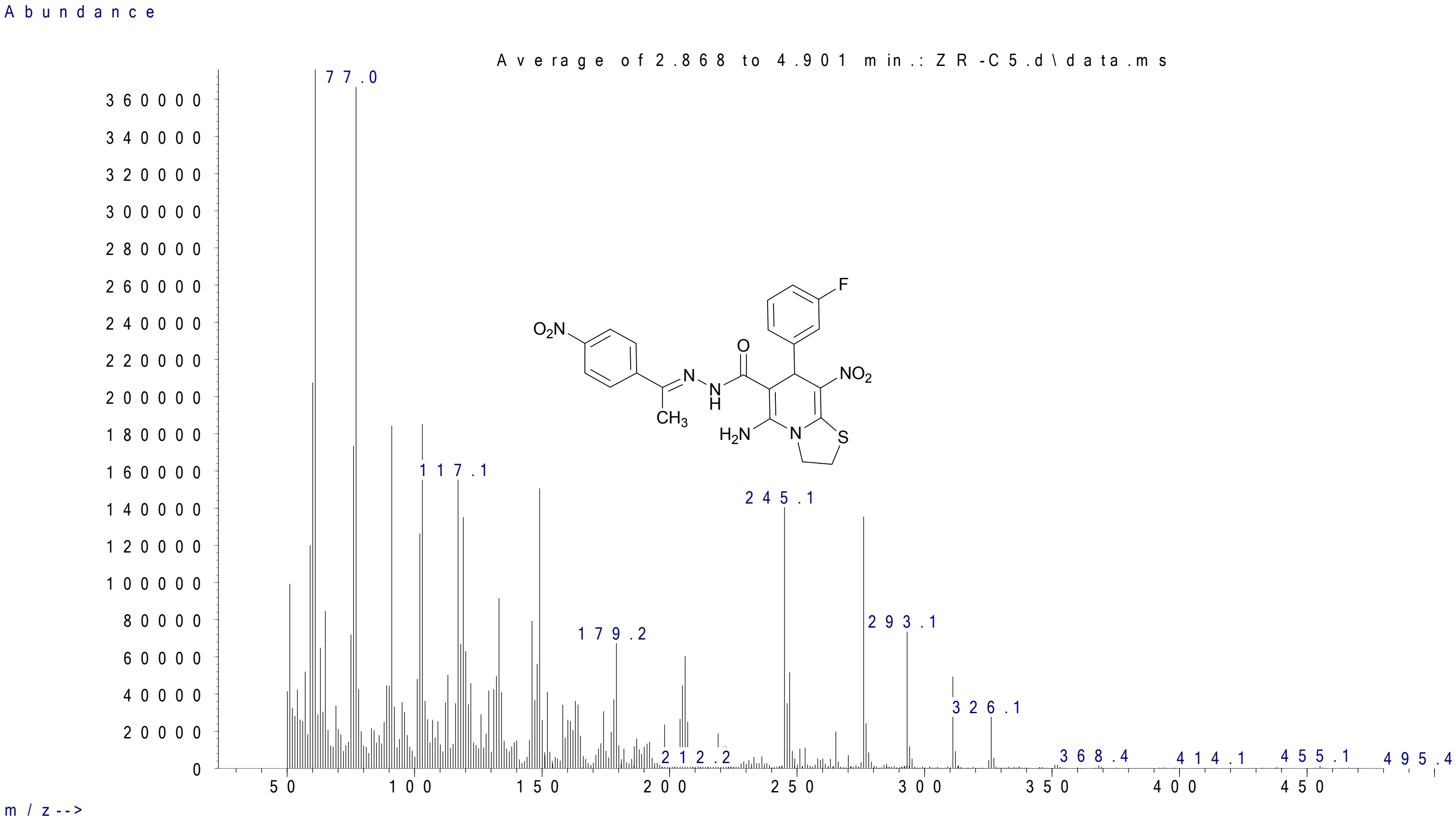
Abundance
Average of 2.868 to 4.901 min.: ZR-C5.d\data.ms
77.0
117.1
179.2
212.2
245.1
293.1
326.1
368.4
414.1
455.1
495.4
m/z-->
O_2N
O
F
NO_2
N
N
H
CH_3
H_2N
S

MS of 6m

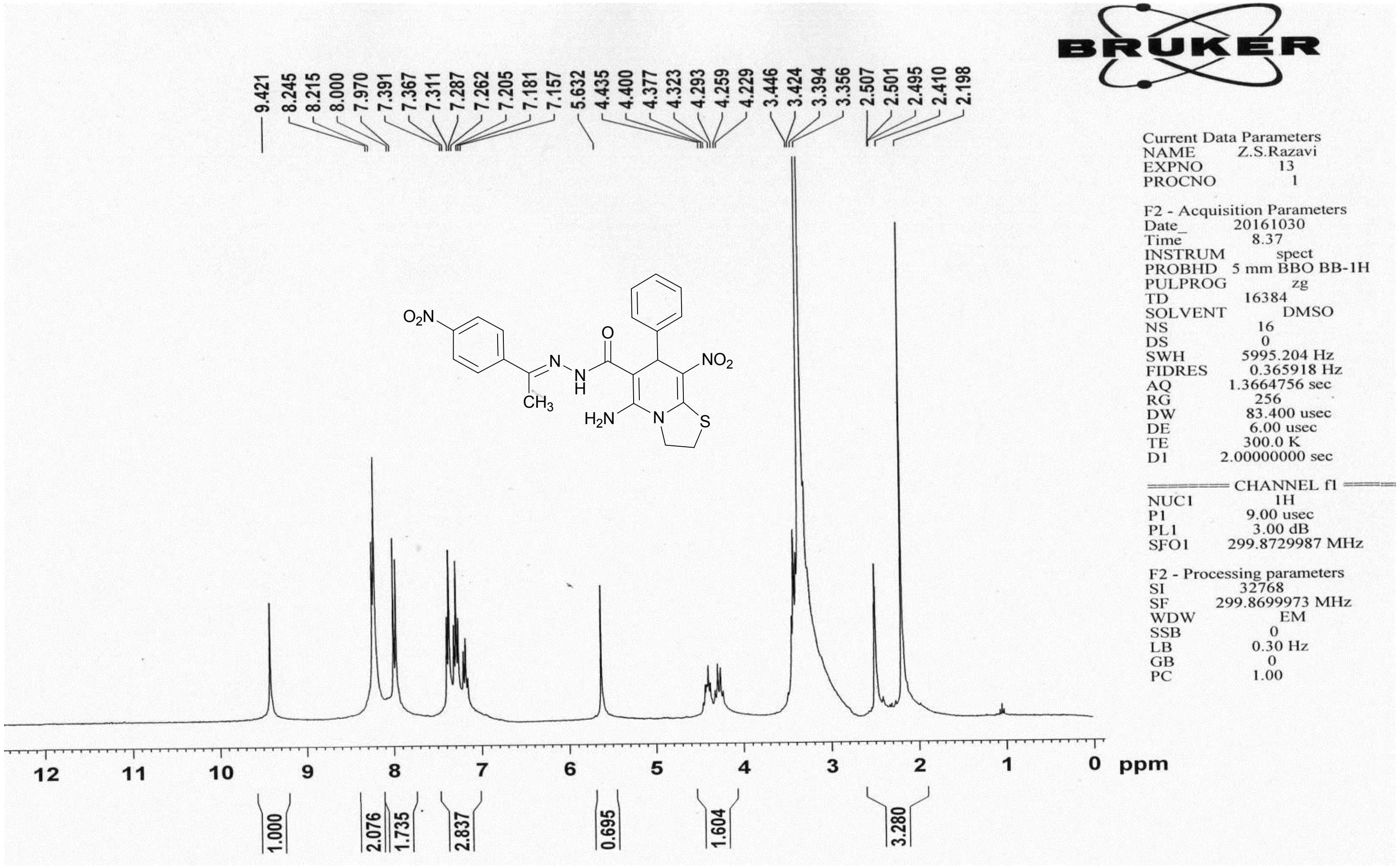
9.421
8.245
8.215
8.000
7.970
7.391
7.367
7.311
7.287
7.262
7.205
7.181
7.157
5.632
4.435
4.400
4.377
4.323
4.293
4.259
4.229
3.446
3.424
3.394
3.356
2.507
2.501
2.495
2.410
2.198
BRUKER
Current Data Parameters
NAME Z.S.Razavi
EXPNO 13
PROCNO 1
F2 - Acquisition Parameters
Date_ 20161030
Time 8.37
INSTRUM spect
PROBHD 5 mm BBO BB-1H
PULPROG zg
TD 16384
SOLVENT DMSO
NS 16
DS 0
SWH 5995.204 Hz
FIDRES 0.365918 Hz
AQ 1.3664756 sec
RG 256
DW 83.400 usec
DE 6.00 usec
TE 300.0 K
D1 2.00000000 sec
CHANNEL f1
NUC1 1H
P1 9.00 usec
PL1 3.00 dB
SFO1 299.8729987 MHz
F2 - Processing parameters
SI 32768
SF 299.8699973 MHz
WDW EM
SSB 0
LB 0.30 Hz
GB 0
PC 1.00
O2N
NO2
O
N
N
H
CH3
H2N
N
S
12 11 10 9 8 7 6 5 4 3 2 1 0 ppm
1.000
2.076
1.735
2.837
0.695
1.604
3.280

^{1}H NMR of 6n

C8-C

Parameter		Value
1	Data File Name	C:/ Users/ ali/ Desktop/ NMR MN/ Masoud-Mohammadivala/ C8-C.fid/ fid
2	Title	C8-C
3	Comment	
4	Origin	Varian
5	Owner	
6	Site	
7	Spectrometer	inova
8	Author	
9	Solvent	dmso
10	Temperature	25.0
11	Pulse Sequence	s2pul
12	Number of Scans	2048
13	Receiver Gain	60
14	Relaxation Delay	1.0000
15	Pulse Width	0.0000
16	Acquisition Time	1.0433
17	Acquisition Date	2020-07-20T16:44:14
18	Modification Date	2020-07-20T15:45:32
19	Spectrometer Frequency	125.62
20	Spectral Width	31409.5
21	Lowest Frequency	-1945.9
22	Nucleus	13C
23	Acquired Size	32768
24	Spectral Size	65536

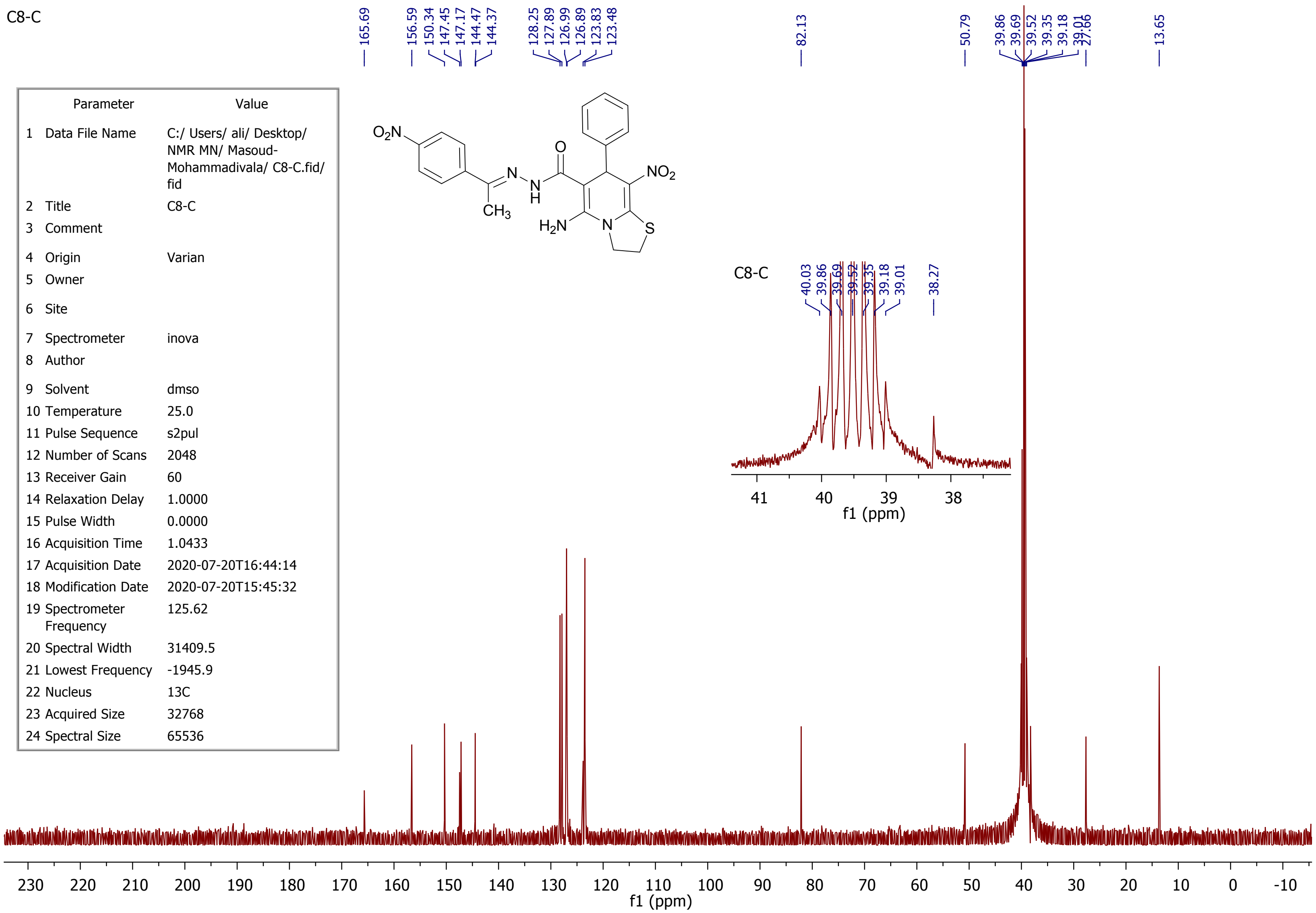

^{13}C NMR of 6n

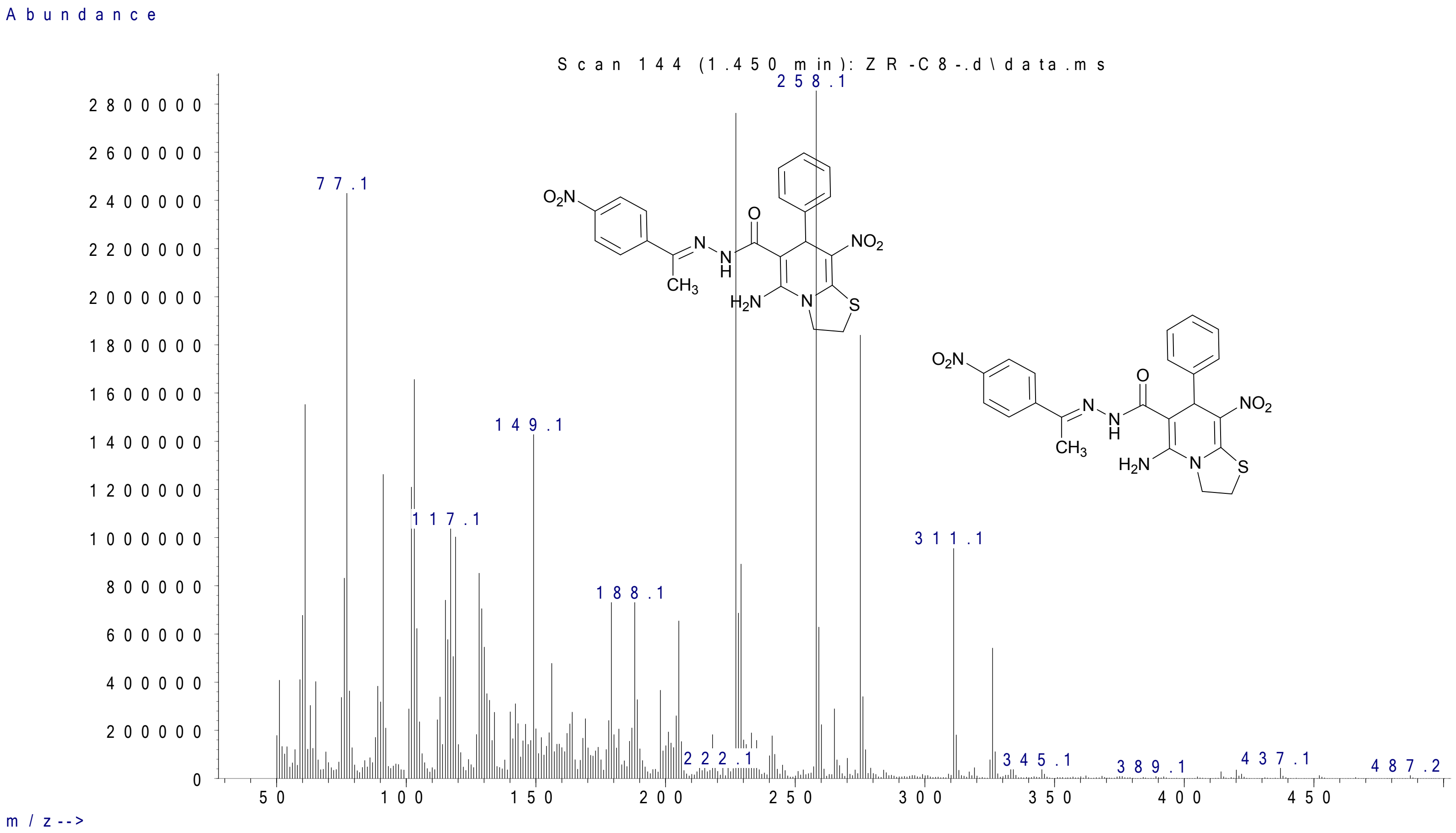
Abundance
Scan 144 (1.450 min): ZR-C8-.d\data.ms
2800000
2600000
2400000
2200000
2000000
1800000
1600000
1400000
1200000
1000000
800000
600000
400000
200000
0
77.1
117.1
149.1
188.1
222.1
258.1
311.1
345.1
389.1
437.1
487.2
50
100
150
200
250
300
350
400
450
m/z-->
O2N
NO2
O
N
N
H
CH3
H2N
S

MS of 6n

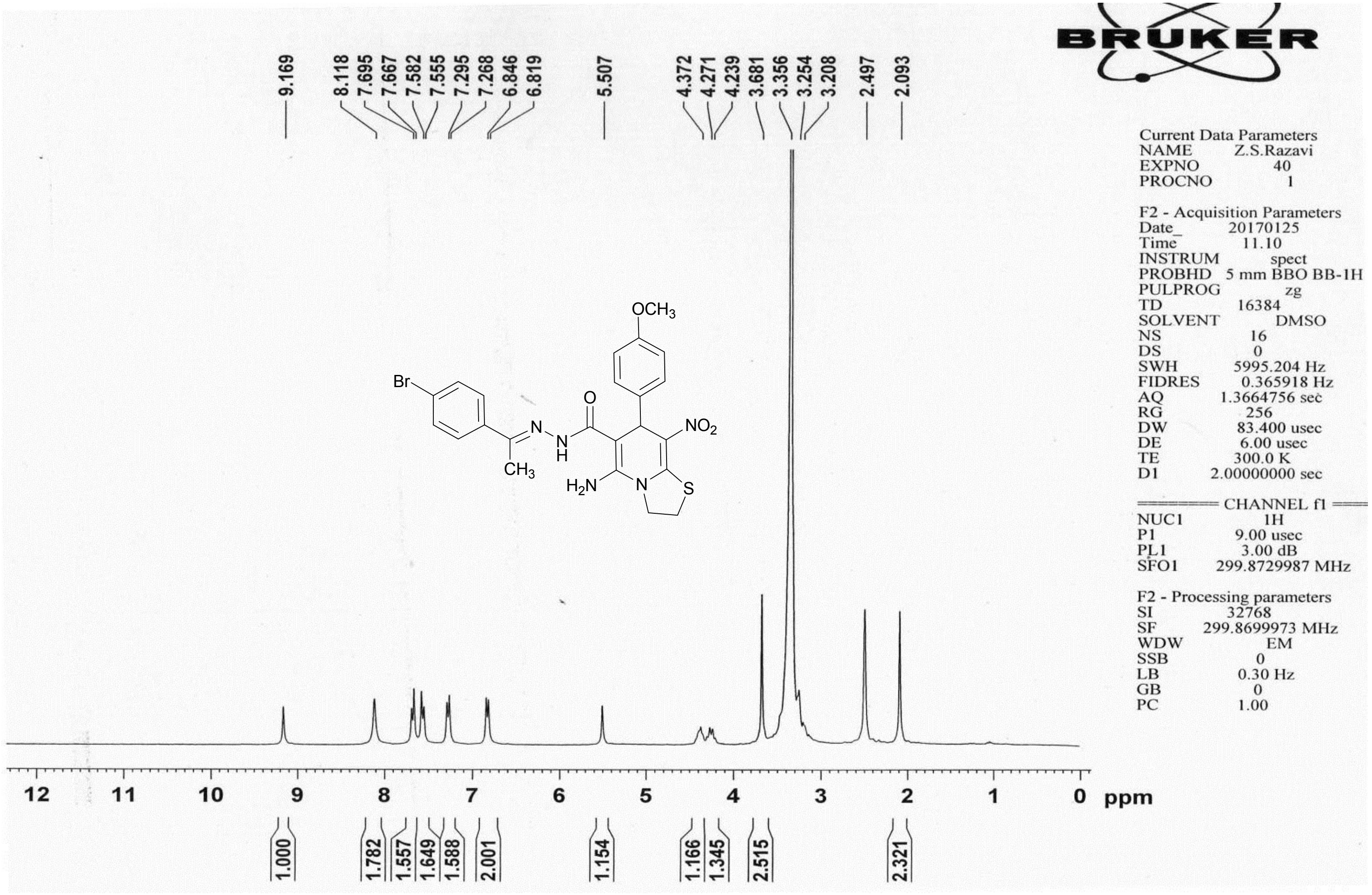
BRUKER
9.169
8.118
7.695
7.667
7.582
7.555
7.295
7.268
6.846
6.819
5.507
4.372
4.271
4.239
3.681
3.356
3.254
3.208
2.497
2.093
Current Data Parameters
NAME Z.S.Razavi
EXPNO 40
PROCNO 1
F2 - Acquisition Parameters
Date_ 20170125
Time 11.10
INSTRUM spect
PROBHD 5 mm BBO BB-1H
PULPROG zg
TD 16384
SOLVENT DMSO
NS 16
DS 0
SWH 5995.204 Hz
FIDRES 0.365918 Hz
AQ 1.3664756 sec
RG 256
DW 83.400 usec
DE 6.00 usec
TE 300.0 K
D1 2.00000000 sec
CHANNEL f1
NUC1 1H
P1 9.00 usec
PL1 3.00 dB
SFO1 299.8729987 MHz
F2 - Processing parameters
SI 32768
SF 299.8699973 MHz
WDW EM
SSB 0
LB 0.30 Hz
GB 0
PC 1.00
OCH3
Br
O
N
N
H
NO2
CH3
H2N
N
S
12 11 10 9 8 7 6 5 4 3 2 1 0 ppm
1.000
1.782
1.557
1.649
1.588
2.001
1.154
1.166
1.345
2.515
2.321

^{1}H NMR of 6o

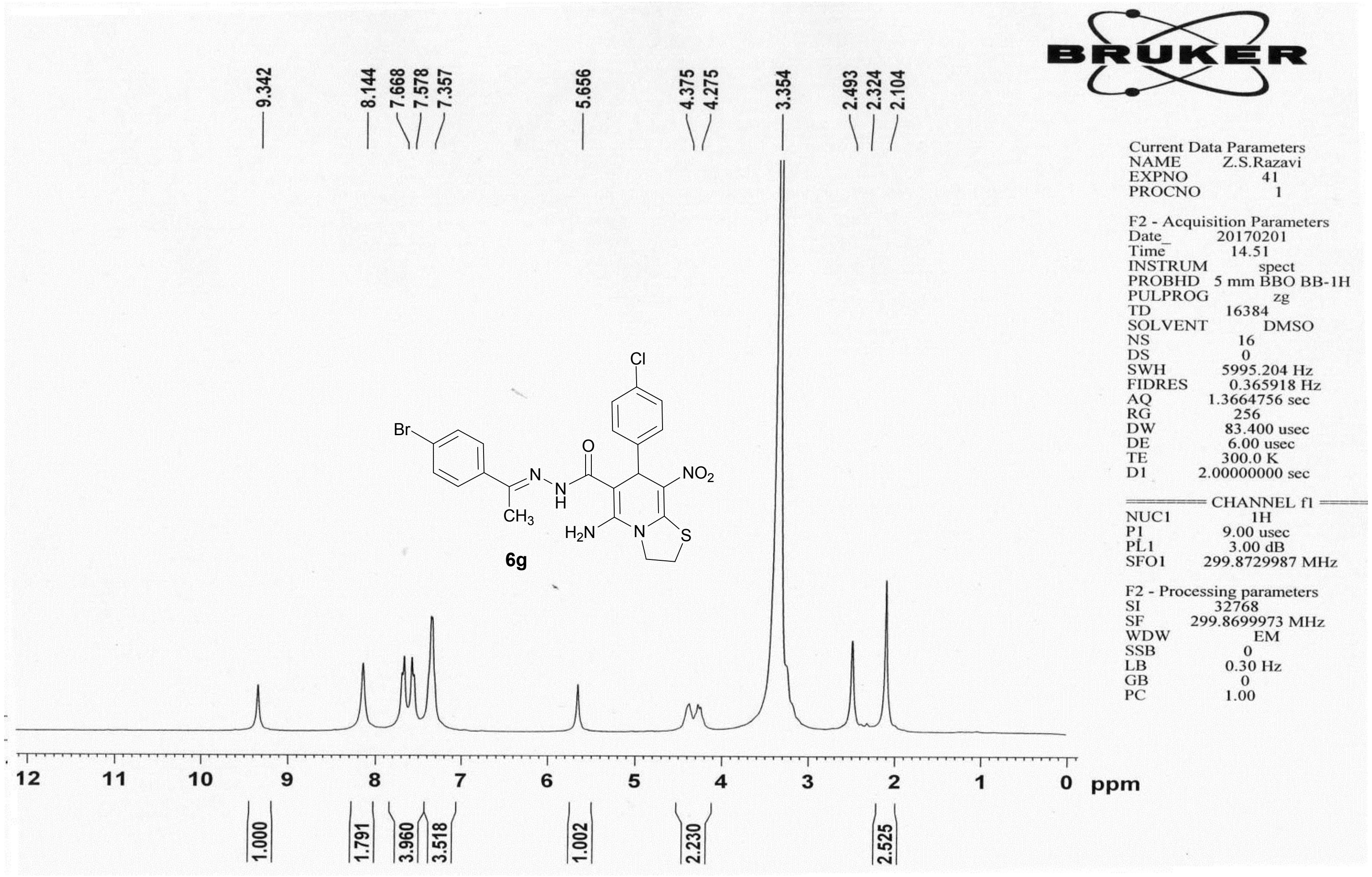
BRUKER
9.342
8.144
7.668
7.578
7.357
5.656
4.375
4.275
3.354
2.493
2.324
2.104
Current Data Parameters
NAME Z.S.Razavi
EXPNO 41
PROCNO 1
F2 - Acquisition Parameters
Date_ 20170201
Time 14.51
INSTRUM spect
PROBHD 5 mm BBO BB-1H
PULPROG zg
TD 16384
SOLVENT DMSO
NS 16
DS 0
SWH 5995.204 Hz
FIDRES 0.365918 Hz
AQ 1.3664756 sec
RG 256
DW 83.400 usec
DE 6.00 usec
TE 300.0 K
D1 2.00000000 sec
CHANNEL f1
NUC1 1H
P1 9.00 usec
PL1 3.00 dB
SFO1 299.8729987 MHz
F2 - Processing parameters
SI 32768
SF 299.8699973 MHz
WDW EM
SSB 0
LB 0.30 Hz
GB 0
PC 1.00
Cl
Br
O
N
N
H
NO2
CH3
H2N
N
S
6g
ppm
1.000
1.791
3.960
3.518
1.002
2.230
2.525

^{1}H NMR of 6p
